# Screening of electrode materials for ammonium ion batteries by high throughput calculation†

**DOI:** 10.1039/d3ra00284e

**Published:** 2023-02-24

**Authors:** Sheqiang Han, Xiaodong Zhang, Qi Song, Bo Zhou, Shangwu Fan

**Affiliations:** a Institute of Modern Physics, Shaanxi Key Laboratory for Theoretical Physics Frontiers, Northwest University Xi'an Shaanxi 710069 People's Republic of China zhoubo@nwu.edu.cn; b Science and Technology on Thermostructural Composite Materials Laboratory, Northwestern Polytechnical University Xi'an Shaanxi 710072 People's Republic of China

## Abstract

Ammonium-ion batteries (AIBs) have attracted intense interest lately as promising energy storage systems due to their light weight, safe, inexpensive, and widely available advantages. It is of great significance to find a fast ammonium ion conductor for the electrode of AIBs that directly affects the electrochemical performance of the battery. Using high-throughput bond-valence calculation, we screened the electrode materials of AIBs with a low diffusion barrier from more than 8000 compounds in the ICSD database. Twenty-seven candidate materials were finally identified by the bond-valence sum method and density functional theory. Their electrochemical properties were further analyzed. Our results, which give the relationship between the structure and electrochemical properties of various important electrode materials which are suitable for AIBs development, may pave the way for next-generation energy storage systems.

## Abstract 2

Twenty-seven candidate materials which are suitable for ammonium ion battery electrodes, are identified by the bond-valence sum method and density functional theory from the ICSD database.

## Graphical abstract

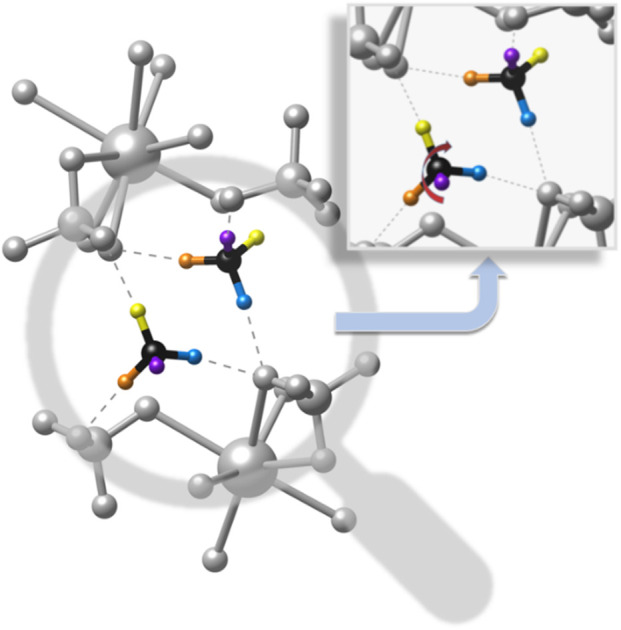

## 1. Introduction

With the worldwide emphasis on renewable energy sources such as solar and wind, battery energy storage has become an essential solution for grid stability and reliability. Traditional metal ion batteries (Li^+^,^1^ Na^+^,^2^ K^+^,^3^*etc.*) are relatively expensive in terms of abundance in Earth's crust and mining cost. Emerging non-metallic batteries (proton,^4^ NH_4_^+^,^5^ Cl^−^,^6^*etc.*) have developed rapidly in recent years. Their cost, capacity, operating voltage, rate performance, and cycle stability are better than metal batteries. Among them, ammonium-ion batteries (AIBs) are promising candidates for large-scale energy storage systems. Compared with metal batteries, ammonium ions have the merit of low molar mass (18 g mol^−1^), rich reserves and being renewable (only containing N and H essential elements), and having smaller hydrated ion sizes.^7,8^ Compared with acidic electrolytes, electrolytes containing NH_4_^+^ show weak acidity and are less corrosive, will not lead to the dissolution of electrode materials and have higher HER (hydrogen evolution reaction) overpotential than electrolytes containing H^+^.^9,10^

The progress of ammonium-ion batteries (AIBs)^5,9,11–15^ is still in its infancy. Ji's team systematically studied the insertion of NH_4_^+^ into layered V_2_O_5_. It was found that ammonium-ion forms strong hydrogen bonds with layered V_2_O_5_, and the diffusion mode of NH_4_^+^ is similar to monkey swing.^11^ Wang also developed NH_4_^+^ storage based on supercapacitor. The research indicates that the ammoniation/deamination mechanism is dominated by non-diffusion controlled pseudo capacitance behavior.^14^ So far, the reported ammonium storage materials are basically MXene,^16^ Prussian blue analogues,^17^ conductive polymers (PANI)^18^ and metal oxides (MoO_3_, V_2_O_5_, MnO_*x*_, Fe_5_V_15_O_39_(OH)_9_·9H_2_O).^12,19,20^ However, the performances achieved to date remain inferior to what lithium-ion batteries can do, showing limited capacity and cycling stability. Therefore, to achieve rechargeable AIBs with excellent performance, it is necessary to explore new electrodes for NH_4_^+^ storage.

At the current stage, the discovery of these energy materials relies predominantly on experimental serendipity and the try-and-error experimental process that are inefficient and time-consuming. Nevertheless, much evidence has shown that the discovery process of new energy materials could be significantly accelerated by the data mining process and high throughput calculation.^21,22^ Now, various experimental and computational repositories such as Inorganic Crystal Structure Database (ICSD),^23^ Crystallography Open Database (COD),^24^ the Materials Project,^25^ and others store thousands of experimental and calculated structures, which already serve as potential batteries electrodes or still wait for such an opportunity.

Meanwhile, A. O. Boev *et al.* extracted 2800 initial structures from the material database and identified 33 potential compounds for cathode materials of lithium-oxygen batteries with low normalized surface energy.^26^ Max Avdeev used high-throughput screening to conduct a detailed analysis of the 13 000 materials in ICSD, and identified the crystal structures featuring infinite networks of pathways suitable for Li^+^, Na^+^, K^+^, Ag^+^, and Cu^+^ ionic transport.^27^ However, there is no report on the high-throughput screening method to determine the candidate electrode materials for ammonium-ion batteries.

As inspired by the ion exchange experiments, the compounds from ICSD (inorganic crystal structures database)^23^ which contain K^+^ ion were chosen. We replaced the K^+^ ions in these compounds with NH_4_^+^ to work as the input of our following screening process, since the K^+^ and NH_4_^+^ ions have the same valence and almost the same ionic radii (149 pm for K^+^ and 148 pm for NH_4_^+^).^28^ Further, we used the bond valence energy landscape method (BVEL),^29–33^ density functional theory (DFT) method and high-throughput screening to find new electrode materials for AIBs. 27 candidate materials were identified. Our theoretical results may open new opportunities for the development of high-performance AIBs.

## 2. Methods

### 2.1 Screening algorithm

**Fig. 1:**
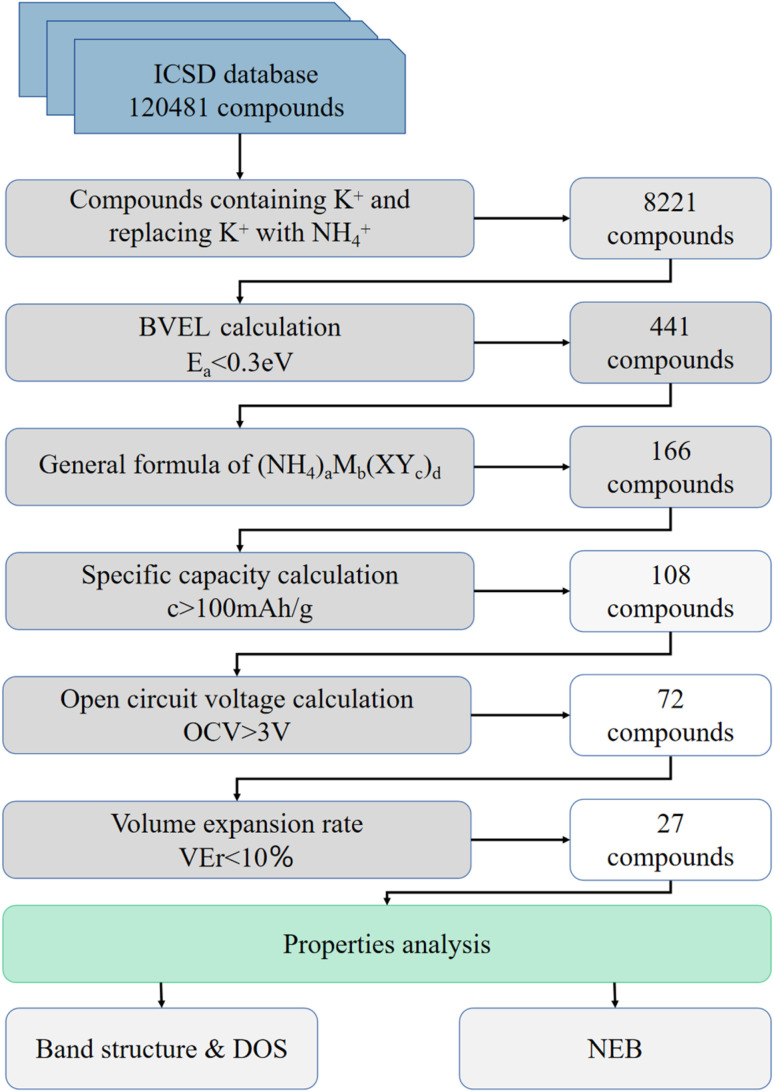
The schema diagram of the screening process. All data relating to screening process are included in the ESI.

The approach can be considered as multi-level screening, where at each higher level the complexity of computational methods increases, while the number of candidates reduces. A total of 120 481 database entries were screened. We selected the world's largest database ICSD for completely identified inorganic crystal structures as the input to the screening framework. The main steps of the filtering algorithm are as follows: (I) Screening compounds containing K^+^ in the database and replacing K^+^ with NH_4_^+^, about 8221 compounds were obtained. (II) From bond valence energy landscape (BVEL) calculation, 441 compounds with diffusion barriers less than 0.3 eV were screened. (III) Considering that structures which are suitable for chemical ion exchange route should have robust framework. 166 compounds with general formula of (NH_4_)_*a*_M_*b*_(XY_*c*_)_*d*_ (M – metal. in which inactive metals such as Zn, Zr, Ga, Ge, Mg, Al, *etc.*, for electrolyte and active transition metal such as Fe, Mn, Ti, Cr, V, Co, Ni, Nb, Mo, *etc.* for electrode material; XY_*c*_ – polyanion groups, X – metalloids such as B, Si, Ge, As and Mo, Nb, *etc.*, Y – O, F, S, *etc.*) were screened.^34^ (IV) 108 compounds were obtained by a filter of specific capacity greater than 100 mA h g^−1^. (V) After the first principle calculation, the 72 compounds were screened using the condition that the open circuit voltage (OCV) was higher than 3 V. (VI) The final screening condition is that the volume expansion rate of the optimized structure is less than 10% compared with the K-contained analog, and 27 compounds are selected (Fig. 1).

### 2.2 Bond valence energy landscape (BVEL) method

The bond-valence method^35^ is the development of the principle of local charge neutrality (first proposed by Pauling). The valence of an atom is the sum of the individual bond valences surrounding the atom. The results show that the dependence of *S*_*ij*_ on the distance between the *i*^th^ and *j*^th^ ions *R*_*ij*_ can be well described by exponential function.
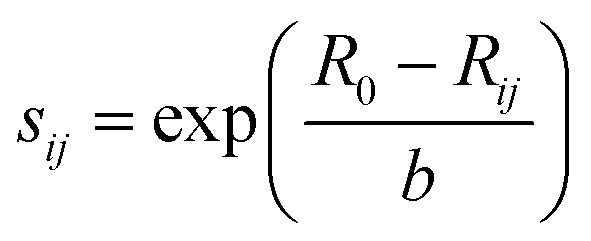
where *R*_0_ and *b* are empirical parameters.^36^

As shown in S. Adams's work,^31^ the bond valence site energy of *i* can then be interpreted as a result of a Morse-type interaction with the adjacent anions *j*, it can be combined with the Coulomb repulsion *E*_repulsion_ of *i* with other immobile cations.
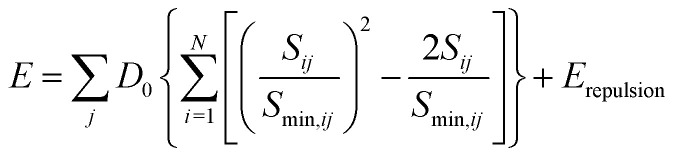

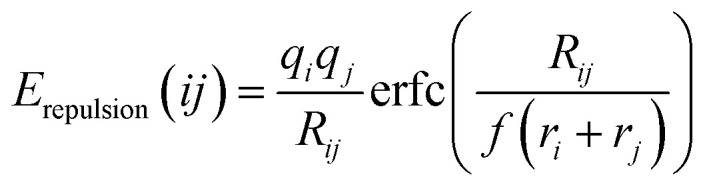
where *r*_*i*_ and *r*_*j*_ are the covalent radii of the respective cation (or anion) pair *i*, *j* and *f* is a screening factor (typically of the order *f* ≈ 0.75).^37^ It may be noted that for the purpose of analyzing *E* landscapes, Coulomb repulsions are considered only between mobile and immobile cations to reveal continuous pathways, while coulomb attraction terms are generally integrated into the Morse attraction term. The charge screening is based on an error function complement term *E*_repulsion_.

The console utilities CrysFML (Crystallographic Fortran 95 Modules Library) which are developed by N. A. Katcho *et al.*^38^ are used for bond-valence energy landscapes calculations. Small Python scripts have been written to bond the input and output between programs to reduce human intervention and facilitate data management. Within the theoretical framework of bond-valence sum, the diffusion barriers (*E*_a_) in three directions of *a*, *b* and *c* of the cell are obtained. The fundamental process that enables the functioning of most of today's rechargeable batteries is the reversible intercalation of ions into electrode. Ionic conductivity emerges as a paramount criterion. We abandon the structures with diffusion barrier higher than 0.3 eV.

#### 2.2.2 Structure and specific capacity selection

Most of the cathode materials for Li-ion or Na-ion batteries have more than 3 elements in their chemical formulas.^39,40^ For AIBs, we focus on compounds with the general formula of (NH_4_)_*a*_M_*b*_(XY_*c*_)_*d*_. And we also eliminate the compounds which have elements with low abundance and high price such as Sc, Y, In or rare-earth metals.

Specific capacity is an indicator to the amount of electric charge stored by the electroactive materials in a unit mass. The formula of the specific capacity is as follows:*C* = *nF*/*M*where *n* is the number of transferred electrons, *M* is the molar mass of the selected compounds, and *F* is Faraday constant (26 801 mA h mol^−1^). We selected compounds with theoretical specific capacity more than 100 mA h g^−1^ for the next round of screening. 108 kinds of materials were screened from 8221 types of materials.

#### 2.2.3 Density functional theory (DFT) calculations

The density functional theory method has emerged as an integral and important part of the screening process for electrode materials design. All calculations were performed with the Vienna *Ab initio* Simulation Package (VASP),^41^ within the projector augmented wave (PAW) approach.^42^ The Python Materials Genomics (pymatgen) materials analysis library was used for all analyses.^43^ All structural relaxations and total energy calculations were carried out using parameters similar to those used in the Materials Project.^44^ The key parameters are the use of the Perdew–Burke–Ernzerhof (PBE) generalized-gradient approximation (GGA)^45^ exchange correlation functional, an energy cutoff of 520 eV and a *k*-point mesh of at least 4377 per atom. The relaxation process is divided into two steps. First, keep the cell volume unchanged, optimize the atomic position, and then optimize the cell fully until the convergence criterion is reached. For structures with fractional occupancy in the database, VCA (virtual crystal approximation)^46^ method is used. VCA method is to scale the local potential, the augmentation charges, and the non-local pseudopotential strength parameters of atoms occupied by fractions from the values provided. We also carried out the statications self-consistent and non-self-consistent calcul to explore the density of states and electronic band structure of compounds.

Open circle voltage (OCV)^47,48^ is determined by the chemical potential difference between cathode and anode, and can be calculated as
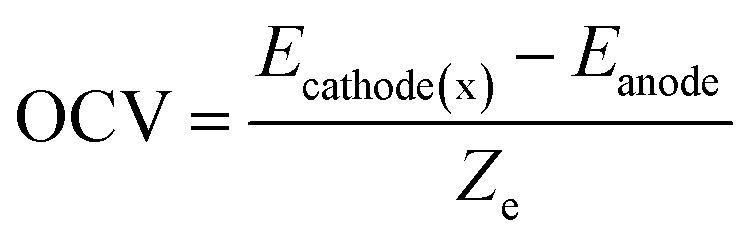
where *E*_cathode_ is defined as the free energy of the intercalated cathode. *E*_anode_ is defined as the free energy of each atom of the anode (NH_4_^+^). *Z*_e_ represents the transferred charge. For NH_4_^+^, we choose a condensed phase similar to body centered cubic lattice, and *Z* is the valence number (*Z* = 1 for NH_4_^+^). The widely used anode in AIBs is conductive polymer materials such as polyaniline (PANI) which has good electronic conductivity, high accommodation capabilities for large ions and light weight.^18^ This anode has higher chemical potential compared with our “fake” pure NH_4_^+^ lattice. Thus, our OCV values are overestimated can only work as upper limit of the expected OCV values.

To further evaluate the ionic conductivities, the nudged elastic band (NEB)^49,50^ method based on DFT is adopted to identify ion migration paths and compute their corresponding energy barriers. Despite the success of the NEB method in characterizing the diffusion process in electrode, the significant computational expense of NEB relative to standard DFT calculations, *e.g.*, geometry relaxations and static energy calculations, has hampered its application to cover all the filtered compounds. Only the final screening compounds are calculated to verify the diffusion barrier calculated by the BVEL method.

## 3. Results and discussion

### 3.1 Screening overview

In the screening algorithm, only the first screening method BVEL diffusion barrier calculation is empirical method. The rest of the filtering is based on first principle calculations. The diffusion barrier, open circuit voltage, and specific capacity are designed for better battery performance. While the general formula of (NH_4_)_*a*_M_*b*_(XY_*c*_)_*d*_ and smaller volume expansion rate compounds are selected to obtain more stable structures. Moreover, the smaller volume expansion rate means that the compound can be more likely synthesized by the ion exchange experiment.

From 8221 compounds containing K^+^ in the ICSD database, we obtained 27 potential electrode materials for AIBs (Table 1). The calculated OCV is in the range of 3.39 V to 6.85 V. The maximum OCV is obtained from NH_4_Zn(BeF_3_)_3_ which contains fluorine atoms. The OCV of (NH_4_)_*a*_M_*b*_(PO_4_)_*d*_ derivatives are about 5.0 V depending on the different metal ion involved. The specific capacity of selected compounds is in the range of 100.33 to 336.97 mA h g^−1^. The maximum specific capacity is calculated from NH_4_Li_2_(BO_3_) which contains only light elements. We also explored their electronic structures within the theoretical framework of density functional theory. Band structures of the screened compounds have been obtained, and the band gaps are shown in Table S6 in ESI.† Further, we have also performed DFT-based NEB calculations for 27 candidate compounds to analyze the feasibility of the screening algorithm and the diffusion properties of candidate materials (Table 2).

**Table 1:** 27 potential electrode materials for ammonium-ion batteries after the screening. ID is the ICSD collection code of the compound containing K^+^ before replacement, and *E* (eV) is the minimum potential barrier value calculated by BVEL. The dimension of the diffusion path (*D*) is judged by barrier energies along the three coordinate axes. Open circuit voltage OCV (V), B_g (eV) is the band gap. The *c* (mA h g^−1^) is the specific capacity. *V* (Å^3^) is the unit cell volume of compounds containing K^+^, *V*′ (Å^3^) is the unit cell volume of compounds containing NH_4_^+^ after replacement and optimization, and Ver (%) is the volume expansion rate

ID	Formula	*E*	*D*	*c*	OCV	B_g	*V*	*V*′	Ver
16 557	NH_4_TiNbO_5_	0.09	2	121.22	5.24	2.88	452.43	483.36	6.84
18 022	NH_4_Zn(BeF_3_)_3_	0.30	3	101.86	6.85	6.14	361.15	390.72	8.19
30 910	NaNH_4_GeO_3_	0.17	2	186.03	4.37	3.31	353.52	378.93	7.19
39 440	NH_4_CrPO_4_F	0.29	3	161.38	4.80	2.57	855.76	910.87	6.44
39 697	(NH_4_)_2_(Zr_0.86_Ti_0.14_)(Si_3_O_9_)(H_2_O)	0.23	3	171.19	3.76	0.04	969.57	1060.22	9.35
39 735	NH_4_GeOPO_4_	0.26	3	145.59	5.04	3.15	794.77	853.99	7.45
48 177	NH_4_Li_2_(BO_3_)	0.26	3	336.97	3.39	4.34	331.05	356.73	7.76
59 281	(NH_4_)_2_MgWO_2_(PO_4_)_2_	0.29	3	124.60	5.06	3.55	898.89	968.91	7.79
59 285	NH_4_(TiO)(PO_4_)	0.24	3	168.48	5.16	3.07	866.17	919.61	6.17
65 260	NH_4_Li_4_(AlO_4_)	0.15	1	273.36	3.95	4.16	889.14	968.35	8.91
69 429	NH_4_(SbO)(SiO_4_)	0.17	3	116.47	5.33	2.67	893.75	954.77	6.83
74 591	NH_4_(TiO)(AsO_4_)	0.19	3	131.97	5.13	3.13	928.52	989.23	6.54
79 650	NH_4_SnO(PO_4_)	0.22	3	116.47	5.30	3.02	925.30	983.87	6.33
79 651	NH_4_(VO)(PO_4_)	0.24	3	165.36	3.98	1.55	864.21	909.35	5.22
79 702	(NH_4_)_2_Ni(WO_2_(PO_4_)_2_)	0.19	3	123.45	4.93	3.38	896.14	964.34	7.61
80 023	NH_4_(TiO)((P_0.56_As_0.44_)O_4_)	0.22	3	150.20	5.15	3.12	894.19	960.44	7.41
80 893	NH_4_GaF(PO_4_)	0.30	3	145.59	5.18	3.98	835.98	895.06	7.07
82 398	(NH_4_)_2_(Be_2_Si_3_O_9_)	0.28	3	217.80	4.92	5.03	829.99	871.74	5.03
82 457	NH_4_Li(Si_2_O_5_)	0.28	2	187.34	4.99	4.98	233.78	255.75	9.40
91 534	NH_4_(Ti_0.936_Sn_0.064_)O(PO_4_)	0.24	3	163.80	4.90	—	870.50	913.43	4.93
200 310	(NH_4_)_2_Ni(MoO_4_)_2_	0.27	2	141.37	5.16	3.85	1747.49	955.38	9.34
200 613	NH_4_Fe(PO_4_)F	0.28	3	157.58	5.17	2.33	874.62	925.15	5.78
203 214	(NH_4_)_2_(Mn_3_(OH)_2_(VO_4_)_2_)	0.30	2	124.89	3.48	1.96	487.51	261.09	7.11
203 218	NH_4_(MoO_2_)(AsO_4_)	0.28	1	100.33	5.24	2.86	493.16	542.13	9.93
280 327	(NH_4_)_2_(TiSi_3_O_9_)(H_2_O)	0.16	3	194.12	5.19	3.46	920.71	985.68	7.06
400 850	(NH_4_)_2_(TiO)_2_(As_0.43_P_0.57_O_4_)_2_	0.21	3	150.57	5.15	3.16	895.69	959.36	7.11
411 501	(NH_4_)_3_(BSb_4_O_13_)	0.21	3	113.67	5.22	1.78	598.67	642.27	7.28

**Table 2:** Diffusion barrier from NEB method *E*_neb (eV) and BVEL method three axis *E*_*a*/*b*/*c* (eV) of 27 potential ammonium-ion battery electrode materials, and their open circuit voltage OCV (V) is also listed

ID	Formula	*E*_*a*	*E*_*b*	*E*_*c*	*E*_neb	OCV
16 557	NH_4_TiNbO_5_	0.09	0.65	—	0.11	5.24
18 022	NH4Zn(BeF_3_)_3_	3.06	3.06	0.30	1.18	6.85
30 910	NaNH_4_GeO_3_	—	0.17	2.00	0.67	4.37
39 440	NH_4_CrPO_4_F	1.00	0.29	1.00	0.69	4.80
39 697	(NH_4_)_2_(Zr_0.86_Ti_0.14_)(Si_3_O_9_)(H_2_O)	0.85	0.85	0.23	0.26	3.76
39 735	NH_4_GeOPO_4_	0.91	0.91	0.26	1.24	5.04
48 177	NH_4_Li_2_(BO_3_)	1.72	0.26	1.72	0.12	3.39
59 281	(NH_4_)_2_MgWO_2_(PO_4_)_2_	0.53	0.49	0.29	0.46	5.06
59 285	NH_4_(TiO)(PO_4_)	0.67	0.67	0.24	0.79	5.16
65 260	NH_4_Li_4_(AlO_4_)	0.15	—	—	0.60	3.95
69 429	NH_4_(SbO)(SiO_4_)	0.72	0.72	0.17	0.83	5.33
74 591	NH_4_(TiO)(AsO_4_)	0.51	0.51	0.19	0.14	5.13
79 650	NH_4_SnO(PO_4_)	0.67	0.67	0.22	1.67	5.30
79 651	NH_4_(VO)(PO_4_)	0.65	0.65	0.24	0.14	3.98
79 702	(NH_4_)_2_Ni(WO_2_(PO_4_)_2_)	0.44	0.44	0.19	0.48	4.93
80 023	NH_4_(TiO)((P_0.56_As_0.44_)O_4_)	0.58	0.58	0.22	0.62	5.15
80 893	NH_4_GaF(PO_4_)	1.17	1.17	0.30	0.21	5.18
82 398	(NH_4_)_2_(Be_2_Si_3_O_9_)	0.77	0.28	0.77	0.49	4.92
82 457	NH_4_Li(Si_2_O_5_)	3.17	0.28	—	0.50	4.99
91 534	NH_4_(Ti_0.936_Sn_0.064_)O(PO_4_)	0.67	0.67	0.24	0.14	4.90
200 310	(NH_4_)_2_Ni(MoO_4_)_2_	0.27	—	0.47	0.54	5.16
200 613	NH_4_Fe(PO_4_)F	0.28	1.03	1.03	0.12	5.17
203 214	(NH_4_)_2_(Mn_3_(OH)_2_(VO_4_)_2_)	—	0.30	2.31	0.12	3.48
203 218	NH_4_(MoO_2_)(AsO_4_)	—	0.28	—	0.69	5.24
280 327	(NH_4_)_2_(TiSi_3_O_9_)(H_2_O)	0.16	0.50	0.49	0.63	5.19
400 850	(NH_4_)_2_(TiO)_2_(As_0.43_P_0.57_O_4_)_2_	0.57	0.57	0.21	0.63	5.15
411 501	(NH_4_)_3_(BSb_4_O_13_)	0.21	0.23	0.67	0.23	5.22

#### 3.1.1 Layered type

After screening, only two compounds have an obvious layered structure, namely NH_4_TiNbO_5_ and (NH_4_)_2_Ni(MoO_4_)_2_. As shown in Table S6.† A recent study^51^ performed ion exchange from KTiNbO_5_ to NH_4_TiNbO_5_. The cell parameters of NH_4_TiNbO_5_ are consistent with those in this paper, which demonstrate *Pnmm* orthorhombic structure with *a* = 6.47 Å, *b* = 3.80 Å, *c* = 19.29 Å, and *V* = 474.06 Å^3^. The calculations in this paper show basically consistent results of *a* = 6.61 Å, *b* = 3.83 Å, *c* = 19.09 Å, and *V* = 483.36 Å^3^. And it has a theoretical open circuit voltage of 5.24 V and a low diffusion barrier of 0.11 eV calculated by NEB (and 0.09 eV calculated by BVEL), which means that it may be a high-performance electrode material for AIBs. Meanwhile, from our screening results, the number of compounds containing Mo is large, which is consistent with the types of electrode materials reported for AIBs. Wherein (NH_4_)_2_Ni(MoO_4_)_2_ has 5.16 V open circuit voltage and 0.47 eV diffusion barrier calculated by BVEL.

**Fig. 2:**
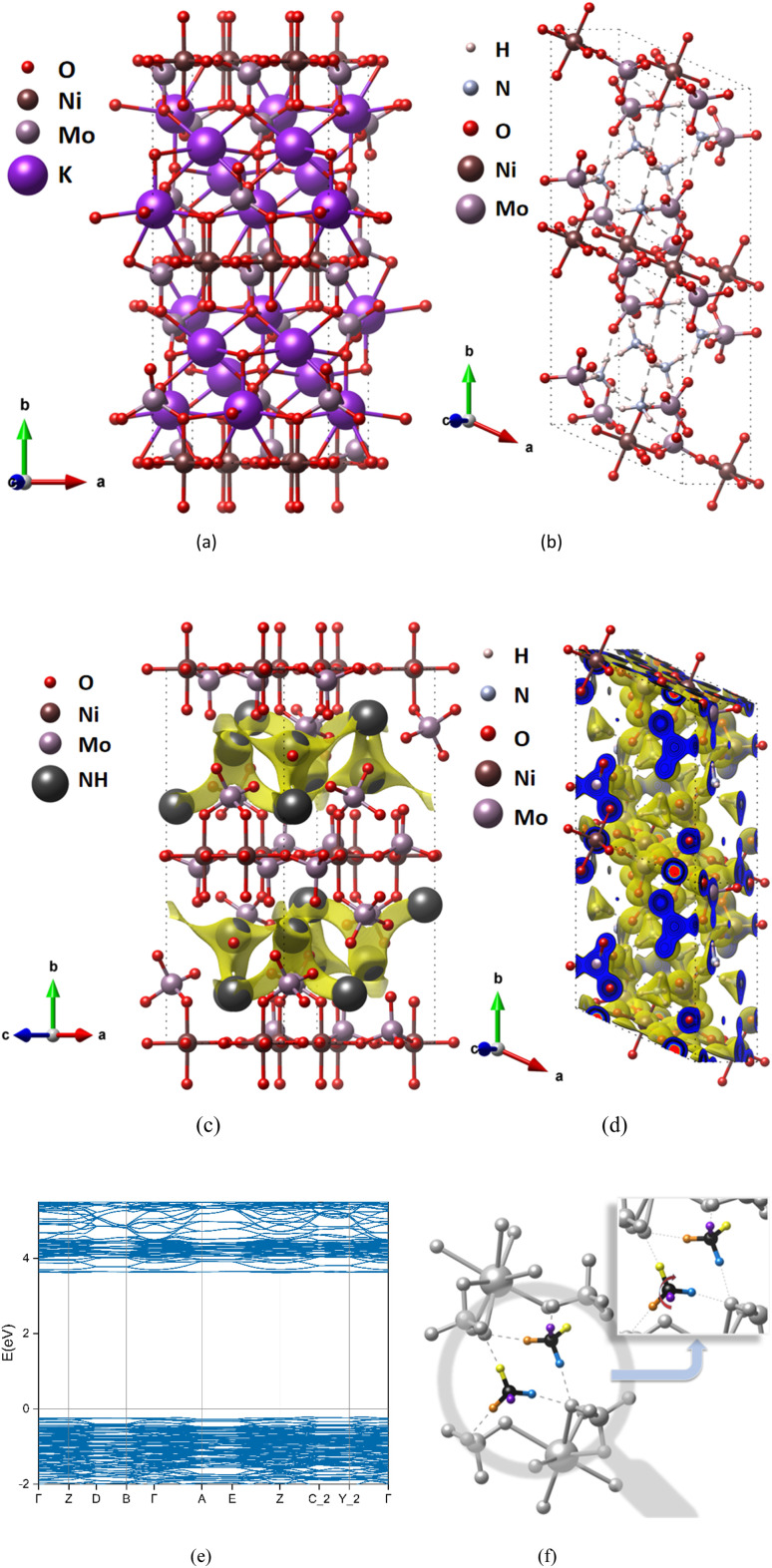
Structure and charge distribution of (K/NH_4_)_2_Ni(MoO_4_)_2_, the atomic type is marked in the upper left corner of the figures, where “NH” represents ammonium-ion. Unoptimized K precursor K_2_Ni(MoO_4_)_2_ (a) and optimized structure after replacing K^+^ with NH_4_^+^ (NH_4_)_2_Ni(MoO_4_)_2_ (b). BVEL data (c), charge density diagram (d) and band structure calculated by DFT (e). The diffusion mode of ammonium ion is revealed by NEB calculation (f), in which ammonium is a colored atom, and the same color represents the same atom before and after diffusion.

We take (NH_4_)_2_Ni(MoO_4_)_2_ as an example. (NH_4_)_2_Ni(MoO_4_)_2_ (Fig. 2(b)) is a compound obtained by replacing K^+^ from K_2_Ni(MoO_4_)_2_ (Fig. 2(a)). The space group is P1, and NH_4_^+^ can migrate along the *a*-axis and *c*-axis. Therefore, after BVEL calculation, as shown in Fig. 2(c), the low-valued isosurface identifies areas with a low energy barrier and therefore represents the most likely pathway for ion transport. In addition, Fig. 2(d) shows the charge density after the DFT calculation. When we replace K^+^ with NH_4_^+^, and after structural relaxation, it is found that the unit cell has a small expansion (Fig. 2(a) and (b)), the unit cell volume of K_2_Ni(MoO_4_)_2_ expanded from 1747.49 Å^3^ to 1910.76 Å^3^ (Tables S4 and S5 in ESI†), At the same time, the symmetry of the structure also changed because of the difference between the tetrahedral structure of NH_4_^+^ and the spherical structure of K^+^. This expansion distortion will not cause the structure of the compound to collapse. From NEB calculation, it is revealed that the strong hydrogen bond makes the diffusion pattern of NH_4_^+^ ions similar to the monkey swing (Fig. 2(f)), which is consistent with the study of Ji's team.^11^ And this diffusion mode is accompanied by the continuous formation and breaking of hydrogen bonds, which can show rapid electrode dynamics and further improve the electrochemical performance of AIBs.

#### 3.1.2 Tunnel type

**Fig. 3:**
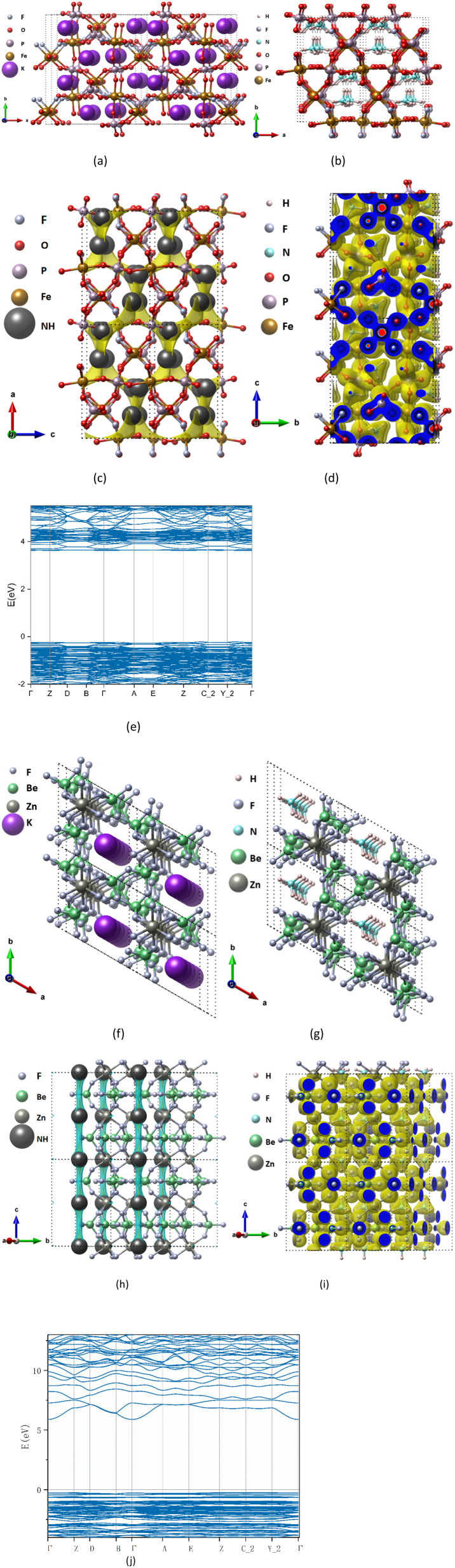
Structure and charge distribution of (K/NH_4_)Fe(PO_4_)F and (K/NH_4_)Zn(BeF_3_)_3_ (2 × 2 × 2 supercell), the atomic type is marked in the upper left corner of the figures, where “NH” represents ammonium-ion. Unoptimized K precursor KFe(PO_4_)F (a), KZn(BeF_3_)_3_ (f) and optimized structure after replacing K^+^ with NH_4_^+^ NH_4_Fe(PO_4_)F (b) NH_4_Zn(BeF_3_)_3_ (g). BVEL data (c and h) and charge density diagram (d and i) and band structure calculated by DFT (e and j).

The tunnel electrode materials account for 25 of the 27 selected materials. To take a further look at NH_4_Fe(PO_4_)F, the minimum BVS mismatch path of NH_4_^+^ shows obvious 3 dimension pathways. From the BVEL data map (isosurface = 0.58 e Bohr^−3^), ammonium-ion diffuses in a zig-zag-shaped path (Fig. 3(c)), which is the same as reported in ref. 52 which the electrode material NaFeSO_4_F of Na-ion batteries was shown.^52^ In these tunnel type electrodes, there is 1 dimension diffusing type electrode. The BVEL map of NH_4_Zn(BeF_3_)_3_ shows a 1 dimension diffusion along the *z*-axis (Fig. 3(g)), which is similar to the reported α-MoO_3_ electrode.^53^ It has a simple tunnel structure, and the formation and fracture of hydrogen bonds are still the primary way of its diffusion, which also guarantees ultrafast kinetics in the reversible insertion/deinsertion process during cycling.^54^ NH_4_CrPO_4_F, NH_4_(TiO)AsO_4_ and (NH_4_)_3_(BSb_4_O_13_) also have very small diffusion barriers and large specific capacity, which is potential candidate for fast ammonium ion storage electrode.

### 3.2 Electronic structure

The preliminary idea to calculate the electronic band structure of a potential electrode material is to determine its metallic, semiconducting or insulating character. The structures and density of states diagrams are listed in Fig. S1–S27 and Tables S7–S33 of the ESI.† Almost all the compounds have band gaps, suggesting poor electronic conductivity. Therefore, further work is needed to improve electronic conductivity.

Among the 27 selected compound, there are eleven (NH_4_)_*a*_M_*b*_(PO_4_)_*d*_ derivatives. The band gap of NH_4_CrPO_4_F, NH_4_GeOPO_4_, (NH_4_)_2_MgWO_2_(PO_4_)_2_, NH_4_(TiO)(PO_4_), NH_4_SnO(PO_4_), NH_4_(VO)(PO_4_), (NH_4_)_2_Ni(WO_2_(PO_4_)_2_), NH_4_(TiO)((P_0.56_As_0.44_)O_4_), NH_4_GaF(PO_4_), NH_4_Fe(PO_4_)F and (NH_4_)_2_(TiO)_2_(As_0.43_P_0.57_O_4_)_2_ is 2.57, 3.15, 3.55, 3.07, 3.02, 1.55, 3.38, 3.12, 3.98, 2.33 and 3.16 eV respectively. The band gap of LiFePO_4_ determined with the *ab initio* GGA+U method is 3.8 eV.^55^ Incorporating of metal ion can efficiently modulate the band gap of (NH_4_)_*a*_M_*b*_(PO_4_)_*d*_ derivatives. The only exception is NH_4_(VO)(PO_4_) which has much smaller band gap (1.55 eV). Also, there are five (NH_4_)_*a*_M_*b*_(Si_*c*_O_*d*_)_*e*_ derivatives. The band gap of these compound is in the range 0.04 eV to 5.03 eV. The maximum band gap is 6.14 eV calculated from fluorid NH_4_Zn(BeF_3_)_3_. The theoretical band gap of (NH_4_)_2_(Zr_0.86_Ti_0.14_)(Si_3_O_9_)(H_2_O) calculated by virtual crystal approximation is very small (0.04 eV) and NH_4_(Ti_0.936_Sn_0.064_)O(PO_4_) does not show band gap. These two values are abnormal requiring further calculation under more complex methodological architectures.

### 3.3 Diffusion barrier calculation

Further, we have also carried out more accurate NEB calculations of the screened 27 compounds. The layered structure of NH_4_TiNbO_5_ and (NH_4_)_2_Ni(MoO_4_)_2_ has small diffusion barrier (0.11 and 0.54 eV) which indicates fast NH_4_^+^ kinetics. From a structural point of view, the compounds with small diffusion barrier have obvious diffusing channel. The selected bulk materials shown in ESI,† have diverse diffusion pattern.

The results (Table 2) show that there is small error between the NEB method based on DFT and the BVEL calculation results for most cases. The main cause of the deviation is that a “static” structure is used during the BVEL calculation. And the cell parameters of the compound remain unchanged after NH_4_^+^ substitution, which further leads to the difference between BVEL and the experimental (or DFT) barrier. For instance, the diffusion barrier of NH_4_Zn(BeF_3_)_3_ and NH_4_(SnO)(PO_4_) using NEB based on DFT optimization shows ∼1 eV higher than the BVEL barrier, which is due to the significant changes in structural parameters compared with its K precursor (specific data are shown in Tables S4 and S5 of ESI†). Despite deviations in the computed values, the high-throughput screening is capable to predict trends in structure–property relationships. The magnitude of deviations can differ substantially for different materials, environments, and thermodynamic factors. The results from the NEB method are more trustable and should have the quantitative agreement of dynamical properties with experimental results. Nevertheless, the BVEL method is still reliable as a rough selection of candidate battery electrode materials. Considering the complexity of the diffusion process of these compounds and the NEB paths considered may be unexhaustive, all the 27 compounds are reserved for further comprehensive study.

## 4. Summary

In this work, we have scanned 8221 K^+^-contained compounds from ICSD to find the proper electrode material for ammonium-ion batteries. The compounds with the exchange of K^+^ ions with NH_4_^+^ ions are used as the input of the following screening process. After the BVEL diffusion barrier filter, general formula filter, OCV screening, specific capacity screening, and volume expansion rate screening, we have successfully predicted 27 potential fast ammonium ion conductors for AIBs, all of which can stimulate further experimental studies and thorough theoretical investigations.

Our research is not exhaustive due to the rich structural chemistry of these filtered compounds, it gives an idea of the research interest in electrode materials for ammonium-ion batteries applications. Due to the simplicity of the BVEL method, its straightforward application to high-throughput schemes has some shortcomings. The empirical parameters of the BVEL method for NH_4_^+^ ions are incomplete. This is the main factor that causes the miss. Although the ionic radii and valence of K^+^ and NH_4_^+^ ions are similar, the stability analysis after the replacement would probably be insufficient, and additional phonon dispersion calculation would be required. Structures with partial occupations need to be properly treated, which requires more complex methodological architectures. Continued effort is undertaken to expand the databases used for screening and solving associated algorithmic and computational problems.

## Data availability

All data generated or analyzed during this study are included in this article and its ESI.†

## Conflicts of interest

The authors declare that they have no known competing financial interests or personal relationships that could have appeared to influence the work reported in this paper.

## Supplementary Material

RA-013-D3RA00284E-s001
