# Supplementary material for: Screening of electrode materials for ammonium ion batteries by high throughput calculation
Source: RSC Adv. 2023 Feb 24;13(10):6548–56. doi: 10.1039/d3ra00284e (PMC9951187; doi:10.1039/d3ra00284e)
Supplement: RA-013-D3RA00284E-s001 [file RA-013-D3RA00284E-s001.pdf]

Electronic Supplementary Material (ESI) for RSC Advances.
This journal is © The Royal Society of Chemistry 2023

Supporting information for:

Screening of electrode materials for ammonium ion battery by high throughput calculation

Sheqiang Han[1], Xiaodong Zhang[1], Qi Song[1], Bo Zhou[11], Shangwu Fan[2]

[1]Institute of Modern Physics, Shaanxi Key Laboratory for Theoretical Physics Frontiers, Northwest University, Xi'an 710069, People's Republic of China.

[2]Science and Technology on Thermostructural Composite Materials Laboratory, Northwestern Polytechnical University, Xi'an, Shaanxi 710072, People's Republic of China.

Summary

This supporting information provides data during the screening process.

[1] E-mail: zhoubo@nwu.edu.cn

Data Description

The basic data is derived from the compounds screened in the ICSD (version 2009). There are 120481 kinds of compounds.

The main steps of the filtering algorithm are as follows:

Screening compounds containing K^+ in the database and replacing K^+ with NH_4^+, about 8221 compounds were obtained. Through bond valence energy landscape (BVEL) calculation, 441 compounds with diffusion barrier less than 0.3 eV were screened (Table S1).

For a robust framework and suitable chemical exchange route, 166 compounds with general formula of $(NH_4)_aM_b(XY_c)_d$ (M - metal. in which, inactive metals such as Zn, Zr, Ga, Ge, Mg, Al etc for electrolyte and active transition metal such as Fe, Mn, Ti, Cr, V, Co, Ni, Nb, Mo, etc for electrode material; XY_c - polyanion groups, X - metalloids such as B, Si, Ge, As and Mo, Nb, etc, Y - O, F, S, etc) were screened (Table S2).

108 compounds were obtained after screening with a specific capacity greater than 100 mAh/g (Table S3).

After the first principle calculation, the 72 compounds (Table S4, S5, S6) were screened by using the condition that the open circuit voltage (OCV) was greater than 3 V. At the same time, Table S6 lists the volume expansion rate of the optimized structure is less than 10% compared with the K-contained analogue, and compounds less than 10% are finally selected.

The structure diagram, density of states diagrams and Structure parameters of 27 compounds finally selected is shown in Figures S1 to S27 and Tables S7 to S33.

Table S1. 441 compounds with diffusion barrier calculated by BVEL less than 0.3 eV were screened. ID is the ICSD Collection Code, STRU is the chemical formula and a, b and c are respectively the diffusion barrier values(eV) calculated by BVEL on the three axes.

ICSD	STRU	a	b	c
158	$(NH_4)_4SnO_4$	0.23	0.24	0.24
228	$(NH_4)_8SiW_{11}O_{39}(H_2O)_{13}$	0.25	0.25	0.25
266	$(NH_4)_6(HgS_4)$	0.30	0.30	0.23
491	$(NH_4)_2Cr_8O_{16}$	-	0.03	-
1120	$(NH_4)_2(Zn_6O_7)$	-	-	0.16
1486	NH_4VO_3	-	1.83	0.14
1512	$(NH_4)_3(MoO_4)(ReO_4)$	0.31	0.29	2.09
1562	$NH_4(Ru_4O_8)$	-	-	0.02
1564	$(NH_4)_4PbO_3$	-	0.06	0.44
1570	NH_4TlO	0.50	0.19	0.63
2060	$(NH_4)_3(Sb_5O_{14})$	0.70	0.05	0.70
2061	$(NH_4)_2Sb_4O_{11}$	0.16	0.16	0.11
2155	$(NH_4)_4(Si_8O_{18})$	0.85	0.16	2.44
2266	$(NH_4)_2(PbO_2)$	0.44	0.44	0.29
2269	$(NH_4)_6Ga_2O_6$	0.10	0.19	0.26
2488	$(NH_4)_6(Mn_2O_6)$	0.33	0.15	0.28
4138	$(NH_4)_3(VO_4)$	0.28	0.28	0.28
4160	$(NH_4)_4SiW_{12}O_{40}$	-	0.14	0.61
4187	$(NH_4)_2((NH_4)_{0.5}Sm_{0.5})(MoO_4)_2$	0.26	0.26	-
4188	$NH_4Ga_{0.33}W_{5.66}O_{18}$	-	-	0.03
4215	$(NH_4)_3SbS_3(Sb_2O_3)_3$	3.26	3.26	0.08
6149	$(NH_4)_6(Fe_2O_6)$	0.10	0.22	0.29
6153	$(NH_4)_6(Co_2O_7)$	0.18	0.29	0.29
6158	$(NH_4)_2PdO_2$	0.50	0.50	0.30
6199	$NH_4(TiO)(PO_4)$	0.66	0.23	0.66
9009	NH_4GaO_2	0.24	0.37	0.29
10290	$NH_4(NO_3)$	1.12	1.12	0.27
10363	NH_4NiCrF_6	0.05	0.05	0.05
10491	$NH_4(TiNbO_5)$	0.09	0.65	-
10492	$(NH_4)_{0.85}Ti_{0.85}Nb_{1.15}O_5$	0.08	0.71	-
10497	NH_4TiTaO_5	0.12	0.31	-
14157	$(NH_4)_3Ni_2O_4$	0.53	0.53	0.21
14158	$(NH_4)_3Pt_2O_4$	0.45	0.45	0.17
14162	$(NH_4)_9Ni_2O_7$	-	0.24	0.24
15095	$NH_4(CuO_2)$	0.04	-	0.14
15115	NH_4AuO_2	0.92	2.49	0.14
15157	NH_4PrO_2	0.17	0.17	-

15517	$NH_4Ti_3TaO_9$	3.49	0.26	-
15518	$(NH_4)_3TiTa_7O_{21}$	0.26	0.26	-
15643	$NH_4Ni(CrF_6)$	0.04	0.04	0.04
15770	NH_4CoO_2	0.26	0.25	-
15929	$(NH_4)_2PbO_3$	0.23	0.23	0.76
16005	$(NH_4)_3(B_3O_6)$	0.83	0.83	0.28
16154	$(NH_4)_2(MoO_4)$	0.93	0.21	0.32
16223	$(NH_4)_2Cd_2O_3$	0.61	0.14	0.14
16264	$(NH_4)_2(ZrO_3)$	0.07	0.48	0.27
16265	$(NH_4)_2(SnO_3)$	0.08	0.45	0.36
16534	$(NH_4)_6(Fe_2O_6)$	0.10	0.21	0.29
16557	NH_4TiNbO_5	0.09	0.65	-
16690	$NH_4(ClO_3)$	0.30	0.38	-
17064	$(NH_4)_6(Si_2O_7)$	0.18	0.31	0.31
18022	$NH_4Zn(BeF_3)_3$	3.06	3.06	0.30
18034	NH_4NiAlF_6	0.12	0.12	0.12
18064	$NH_4Nb(WO_6)$	0.07	0.07	0.07
18301	$(NH_4)_2(Zr_2O_5)$	0.11	0.37	0.24
20021	$NH_4HgI_3H_2O$	-	-	0.09
20141	$(NH_4)_2UO_4$	0.27	0.27	-
20478	$NH_4Y(MoO_4)_2$	0.10	-	0.72
20510	$(NH_4)_2(UO_4)$	0.24	0.24	-
20569	$(NH_4Cl)_2HgCl_2H_2O$	1.75	1.75	0.27
20970	$NH_4(TiO)(PO_4)$	0.66	0.23	0.66
21069	$(NH_4)_2NiO_2$	0.16	0.16	-
22063	$(NH_4)_2PbO_3$	-	0.31	0.19
23184	$(NH_4)_2MnF_4$	0.04	0.04	-
23426	$NH_4Cr_{0.333}W_{1.667}O_6$	0.06	0.06	0.06
23588	$NH_4(FeF_4)$	-	0.25	-
23633	$(NH_4)_4Be_2O_4$	0.26	0.26	0.26
24131	$(NH_4)_2(SnO_3)$	0.34	0.34	0.12
24818	NH_4AgO	0.82	0.82	0.19
25004	$(NH_4)_2CdO_2$	-	-	0.06
25744	NH_4AgO	0.81	0.81	0.29
26147	$NH_4(PO_3)$	0.69	0.30	0.70
26181	$(NH_4)_2(WO_4)$	0.80	0.20	0.31
26258	$(NH_4)_2PtS_2$	1.04	1.04	0.28
26547	$(NH_4)_2H_5(PO_4)(P_2O_7)$	-	0.14	0.20
26632	$NH_4Ag(CO_3)$	-	0.26	0.26
26735	$(NH_4)_2S$	0.15	0.15	0.15
27410	$(NH_4)_2(HgO_2)$	0.15	0.15	-
27584	$NH_4Na_3(Al_4Si_4O_{16})$	2.93	2.93	0.21
27596	$NH_4Na_3(Al_4Si_4O_{16})$	2.89	2.88	0.21
28408	$(NH_4)_3Sb_5O_{14}$	0.74	0.08	0.76

28493	$NH_4Sb_3O_5$	1.55	1.75	0.27
30247	$(NH_4)_2Pb_2Ge_2O_7$	0.13	0.13	-
30886	$NH_4Cu(PO_4)$	-	0.14	0.22
30910	$NaNH_4GeO_3$	-	0.17	2.00
30945	$(NH_4)_2GeO_3$	-	0.49	0.14
30964	NH_4LiO	-	0.21	0.20
31021	NH_4AgCO_3	-	0.26	0.26
31285	$(Na_{0.14}(NH_4)_{0.86})(Mg_{1.44}Fe_{0.56})(Mg_{0.23}Fe_{0.16}Al_{2.61})(Si_{10.36}Al_{1.64}O_{30})$	2.22	2.22	0.24
31686	$(NH_4)_3(B_3O_6)$	0.23	0.23	0.23
31983	NH_4MnO_2	0.30	0.15	0.95
32023	$(NH_4)_{5.52}Ta_{15.76}O_{42.00}$	0.20	0.20	3.45
32743	NH_4NaO	0.10	0.10	-
33294	$(NH_4)_4(WO_5)$	0.27	0.14	0.27
33706	$NH_4(VO_3)$	0.11	1.68	-
33797	$Rb_2(NH_4)_4((CoO_2)O(CoO_2))$	0.28	0.28	-
33808	$(NH_4)_4(Be_3O_5)$	0.15	0.15	0.36
33920	$(NH_4)_3OBr$	0.21	0.21	0.21
34392	$(NH_4)_6(Mn_2O_6)$	0.34	0.16	0.28
34446	$NH_4(H_2PO_4)$	0.22	0.22	0.22
34603	$(NH_4)_2ZnO_2$	0.31	-	0.04
34958	NH_4ScO_2	0.17	0.17	-
35030	$(NH_4)_4Nb_6O_{17}$	0.33	-	0.16
35367	$(NH_4)_6(Co_2O_7)$	0.19	0.30	0.30
35463	$(NH_4)_4(Mo_8P_{12}O_{52})$	0.07	1.97	0.07
36202	$NH_4(NO_2)$	0.08	0.08	-
36380	$(NH_4)_2Na(FeO_3)$	0.27	0.12	0.53
36612	$(NH_4)_2Na_4((FeO_3)_2)$	2.75	0.30	2.75
37204	$(NH_4)_3(NO_3)$	0.26	0.26	0.57
37268	$(NH_4)_4PbO_4$	0.26	0.25	0.26
37271	$(NH_4)_4GeO_4$	0.21	0.21	0.20
37324	NH_4AgO	0.81	0.81	0.18
37325	NH_4CuO	0.73	0.73	0.20
38005	$Na_{0.98}(NH_4)_{0.02}NbO_3$	0.32	0.88	0.26
38301	$(NH_4)_2SnS_3(H_2O)_2$	0.30	0.80	0.80
38325	$Na(NH_4)_3Th_2O_6$	0.11	0.11	-
39440	NH_4CrPO_4F	1.00	0.29	1.00
39463	$NH_4SbO(GeO_4)$	0.56	0.56	0.13
39560	$NH_4FeF(PO4)$	1.08	1.08	0.28
39561	$NH_4(TiO)(PO_4)$	0.67	0.67	0.24
39585	$NH_4TaO(GeO_4)$	0.40	0.40	0.13
39697	$(NH_4)_2(Zr_{0.86}Ti_{0.14})(Si_3$	0.85	0.85	0.23

	$O_9)(H_2O)$			
39735	NH_4GeOPO_4	0.91	0.91	0.26
39882	$NH_4(Ge_{0.063}Ti_{0.937}O)(PO_4)$	0.68	0.68	0.23
39950	$NH_4(Ge_{0.042}Ti_{0.958}O)(PO_4)$	0.67	0.68	0.23
39951	$NH_4(Ge_{0.184}Ti_{0.816}O)(PO_4)$	0.69	0.69	0.23
40154	NH_4AgO	0.81	0.81	0.18
40158	NH_4CuO	0.73	0.73	0.17
40266	NH_4PrO_2	0.18	0.18	-
40267	NH_4CrO_2	0.27	0.27	-
40430	$(NH_4)_2PtSe_2$	1.29	1.29	0.27
40819	$(NH_4)_{0.6}VS_2$	0.09	0.09	-
44528	$NH_4(VO_3)$	0.13	1.94	-
47105	$NH_4Na_2(CuO_2)$	0.25	0.25	-
47218	$(NH_4)_6(HgS_4)$	0.29	0.29	0.23
47223	$(NH_4)_4(IrO_4)$	0.26	0.26	0.36
48118	$NH_4Na_4(GaO_4)$	0.38	0.13	2.46
48119	$Cs(NH_4)_4(GaO_4)$	-	0.06	-
48169	$(NH_4)_5(TlO_4)$	-	0.08	0.24
48177	$NH_4Li_2(BO_3)$	1.72	0.26	1.72
48180	$(NH_4)_3CuO_2$	-	0.11	0.11
49008	NH_4CuS	0.49	0.18	0.18
52376	$NH_4(NO_3)$	0.20	0.24	0.24
54149	$(NH_4)_{0.97}((Ti_{0.97}Nb_{0.03})O)(PO_4)$	0.71	0.71	0.26
55856	$(NH_4)_7(H_3O)_5(As_2W_{18}Ce_3O_{71}(H2O)_3)(H_2O)_9$	-	0.45	0.25
56869	$NH_4(Sb_2PO_8)$	-	3.21	0.27
59281	$(NH_4)_2MgWO_2(PO_4)_2$	0.53	0.49	0.29
59285	$NH_4(TiO)(PO_4)$	0.67	0.67	0.24
59820	$NH_4Sc(HAsO_4)_2$	0.15	-	0.15
59887	$(NH_4)_3Bi_2(AsO_4)_3$	-	0.63	0.26
60103	$(NH_4)_2GeO_3$	-	0.48	0.14
60439	$(NH_4)_2S$	0.13	0.13	0.13
60440	$(NH_4)_2Se$	0.16	0.16	0.16
60441	$(NH_4)_2Te$	0.17	0.17	0.17
60525	$NH_4(AlF_4)$	0.28	-	-
60615	$NH_4(FeF_4)$	0.23	-	3.21
60651	$(NH_4)_6(Ge_2O_7)$	0.18	0.30	0.29
61038	$(NH_4)_2Mn_2O_3$	0.68	0.19	0.19
61105	$(NH_4)_{0.08}YClO_{0.82}$	0.14	0.14	-
61221	$(NH_4)_{0.26}WO_3$	-	-	0.03

61222	$(NH_4)_{0.26}WO_3$	-	-	0.03
61378	$(NH_4)_2Ru_8O_{16}$	-	-	0.02
61388	$NH_4(Sb_2PO_8)$	-	3.06	0.28
61403	NH_4NaMnO_2	0.38	0.38	0.27
62053	$(NH_4)_6CdO_4$	-	-	0.05
62064	$Cs(NH_4)_2AuO_2$	0.63	0.21	0.32
62102	$(NH_4)_9Li_3(IO_6)_2O$	0.50	0.50	0.10
62130	$(NH_4)_2Nb_{10}W_7O_{47}$	-	0.14	-
62136	$(NH_4)_2Na_4(GaO_3)_2$	2.84	0.29	2.84
62138	$(NH_4)_2PbO_3$	0.52	0.30	0.13
62146	$(NH_4)_2Zn_3O_4$	2.68	2.68	0.13
62166	$(NH_4)_3(Sb_3P_2O_{14})$	0.04	0.04	-
62287	$NH_4Mn_4(PO_4)_3$	-	-	0.21
62650	$Na_4(NH_4)_2(Ge_2O_7)$	0.22	0.26	3.08
62656	$(NH_4)_4(ZnO_3)$	0.19	0.19	0.19
63048	$(NH_4)_3Ho(Si_3O_9)$	0.50	0.51	0.08
63489	$(NH_4)_3Na_2(InO_4)$	0.26	0.26	0.26
63544	$NH_4Ni(AsO_4)$	0.17	0.17	-
63563	$(NH_4)_{1.88}Nb(WO_6)$	0.01	0.01	0.01
65163	$NH_4(OD)$	0.27	0.18	0.27
65164	$NH_4(OD)$	0.27	0.18	0.27
65260	$NH_4Li_4(AlO_4)$	0.15	-	-
65291	$(NH_4)_3(LiSiO_4)$	-	0.27	0.34
65345	$(NH_4)_6Lu_2(Si_6O_{18})$	0.44	0.47	0.08
65420	$NH_4(Mo_2P_3O_{13})$	2.05	0.06	0.06
65448	$(NH_4)_6(MnS_4)$	0.29	0.29	0.27
65453	$(NH_4)_2(MnS_2)$	0.77	0.77	0.18
65456	$(NH_4)_2(MnSe_2)$	0.97	0.97	0.29
65644	$(NH_4)_2Tm_{23.33}S_{36}$	-	-	0.21
65656	$NH_4Mo(P_2O_7)$	2.77	2.13	0.28
65682	$NH_4MoW(P_3O_{12})$	-	0.25	1.68
65942	$Cs_2(NH_4)_4(Fe_2O_5)$	0.24	-	0.29
65976	$(NH_4)_{11}(Li(MnO_4)_4)$	0.30	0.30	0.47
65978	$(NH_4)_3Na_2(LiTeO_6)$	0.21	-	0.11
66073	$(NH_4)_4(SiO_4)$	0.20	0.13	0.15
66275	$(NH_4)_2(HgO_2)$	0.14	0.14	-
66570	$NH_4(TiO)(PO_4)$	0.66	0.66	0.23
66774	$(NH_4)_6(Ti_2O_7)$	0.18	0.33	0.33
67120	$(NH_4)_{0.84}(Nb_{0.08}Ti_{0.92})OPO_4$	0.66	0.66	0.22
67170	$(NH_4)_{14}(Fe_4O_{13})$	-	0.18	0.07
67284	$(NH_4)_2(W_2O_7)$	1.19	-	0.16
67387	$(NH_4)_2CoS_2$	0.91	0.91	0.21
67390	$(NH_4)_2CoSe_2$	1.05	1.05	0.29

67396	$(NH_4)_6(Ti_2O_7)$	0.18	0.33	0.33
67397	$(NH_4)_6(Ti_2O_7)$	0.23	0.35	0.35
67738	$(NH_4)_2O(Nb_2O_5)_7$	-	-	0.04
67961	$(NH_4)_{14}(In_4O_{13})$	-	0.18	0.07
68361	$(NH_4)_8(U_2O_{10})$	0.20	0.20	0.13
68496	$(NH_4)_{0.75}MoNbO(PO_4)(P_2O_7)$	-	0.25	1.60
68505	$(NH_4)_4OBr_2$	0.30	0.30	0.38
68533	$(NH_4)Mo_4O_6$	-	-	0.12
68603	$(NH_4)_6(CoS_4)$	0.31	0.31	0.30
68705	$NH_4(TiO)(PO_4)$	0.66	0.66	0.23
68706	$NH_4SnO(PO_4)$	0.78	0.78	0.22
68989	$NH_4(GaO_2)$	0.28	0.40	0.32
69019	NH_4GaO_2	0.46	0.27	0.28
69429	$NH_4(SbO)(SiO_4)$	0.72	0.72	0.17
69556	$NH_4VO(PO_4)$	0.67	0.25	0.67
71536	$(NH_4)_2Co(SeO_3)_2$	0.10	0.10	-
71540	$(NH_4)_2Mn(SeO_3)_2$	0.09	0.09	-
71803	$(NH_4)_3Nd(Si_6O_{15})$	0.38	0.39	0.19
71904	$NH_4(TiO)((P_{0.58}As_{0.42})O_4)$	0.58	0.58	0.22
71927	$(NH_4)_6Cr_{0.38}Nb_{15.62}O_{42}$	0.26	0.26	-
72051	$NH_4(TiO)((P_{0.5}As_{0.5})O_4)$	0.59	0.21	0.59
72514	$(NH_4)_3(Nb_8O_{21})$	0.27	0.27	-
72720	$NH_4(Ti_{.496}Sn_{.504})O(PO_4)$	0.65	0.24	0.65
72736	$NH_4(ScF_4)$	-	0.24	-
72873	$(NH_4)_3(NbP_2O_9)$	0.23	0.93	0.93
72976	$(NH_4)_2Bi_8Se_{13}$	-	-	0.27
73136	$Rb_4(NH_4)_2(Fe_2O_5)$	0.27	0.57	0.73
73205	$(NH_4)_5Li(Ge_2O_7)$	0.26	0.53	0.26
73212	$(NH_4)_3CoO_2$	0.21	0.17	0.21
73260	$(NH_4)_6CrNb_{15}O_{42}$	0.27	0.27	-
73550	$(NH_4)_6(Ge_2O_7)$	0.18	0.31	0.30
73807	$NH_4Mo_4O_6$	-	-	0.12
74193	$(NH_4)_2La_2(Ti_3O_{10})$	0.20	0.20	-
74300	$(Rb_{0.88}(NH_4)_{0.12})_2((NH_4)_{0.91}Rb_{0.09})_2(GeO_3)_2$	-	0.29	1.69
74591	$NH_4(TiO)(AsO_4)$	0.51	0.51	0.19
74592	$NH_4(TiO)(AsO_4)$	0.48	0.48	0.16
74593	$NH_4(TiO)(AsO_4)$	0.48	0.48	0.15
74594	$NH_4(TiO)(AsO_4)$	0.47	0.47	0.16
74873	$(NH_4)_6(Pb_2O_5)$	0.48	0.50	0.27
74874	$(NH_4)_4(PbO_3)$	-	0.06	0.46

74911	$(NH_4)_5(InO_4)$	-	0.28	0.27
74940	$(NH_4)_2(CoO_2)$	0.22	0.19	0.19
74955	$Cs(NH_4)_4(TlO_4)$	-	0.09	-
74956	$(NH_4)_4Na_2(Tl_2O_6)$	0.28	0.07	0.97
74968	$(NH_4)_6(Al_2O_6)$	0.11	0.21	0.27
75322	$NH_4(TiO)(AsO_4)$	0.51	0.51	0.18
76891	$(NH_4)_2Pb(SO_4)_2$	0.09	0.09	-
76935	$(NH_4)_3Na(UO_2)(CO_3)_3$	2.76	2.76	0.04
77333	$NH_4(SbO_3)$	0.16	0.16	0.16
78891	$(NH_4)_3OCl$	0.13	0.13	0.13
79101	$(NH_4)_4(SnO_3)$	-	0.06	0.41
79200	$NH_4(Bi_3S_5)$	-	0.29	-
79535	$(NH_4)_{0.95}Mo_4O_6$	-	-	0.12
79650	$NH_4(SnO)(PO_4)$	0.67	0.67	0.22
79651	$NH_4(VO)(PO_4)$	0.65	0.65	0.24
79702	$(NH_4)_2Ni(WO_2(PO_4)_2)$	0.44	0.44	0.19
79711	$NH_4(AlAs_2O_7)$	1.63	-	0.23
80022	$NH_4(TiO)((P_{0.75}As_{0.25})O_4)$	0.61	0.61	0.22
80023	$NH_4(TiO)((P_{0.56}As_{0.44})O_4)$	0.58	0.58	0.22
80024	$NH_4(TiO)((P_{0.38}As_{0.62})O_4)$	0.55	0.55	0.20
80336	$(NH_4)_{0.25}IrO_2$	-	0.02	-
80893	$NH_4GaF(PO_4)$	1.17	1.17	0.30
80976	$NH_4(SnO)(AsO_4)$	0.52	0.52	0.18
81478	$Cs_2(NH_4)_2B_{10}O_{17}$	-	0.27	1.18
81479	$(NH_4)_6(VO)_4(SO_4)_8$	0.62	3.01	0.07
81541	$(NH_4)_{0.99}(Mo_4O_{0.31})O_6$	-	-	0.12
81864	$(NH_4)_2(AlF_5)$	0.20	0.20	0.37
82276	$(NH_4)_2Cr_8O_{16}$	-	-	0.04
82391	$NH_4(GaAs_2O_7)$	2.02	-	0.22
82398	$(NH_4)_2(Be_2Si_3O_9)$	0.77	0.28	0.77
82457	$NH_4Li(Si_2O_5)$	3.17	0.28	-
82601	$(NH_4)_{0.998}((Ti_{0.998}W_{0.002})O)(PO_4)$	0.67	0.24	0.67
82905	$(NH_4)_2La_2(Ti_3O_{10})$	0.20	0.20	-
83285	$(NH_4)(Fe_{11}O_{17})$	0.12	0.12	-
83431	$(NH_4)_2Ca_2(CO_3)_3$	0.47	0.47	0.29
83843	$(NH_4)_2(Te_4O_9)$	-	0.58	0.29
83844	$(NH_4)_2(Te_2O_5)$	0.21	0.90	3.22
85404	$(NH_4)_6(CuSi_2O_8)$	0.18	0.23	0.43
85505	$(NH_4)_2Ca(NO_2)_4$	0.12	0.12	-
85583	$(NH_4)_2Au_4CdS_4$	0.81	0.27	1.66

85750	$(NH_4)_3Al_5(HPO_4)_6(PO_4)_2(H_2O)_{18}$	0.12	0.12	-
85751	$(NH_4)_3Al_5(DPO_4)_6(PO_4)_2(D_2O)_{18}$	0.15	0.15	-
86784	$NH_4Fe_{0.43}Nb_{1.57}O_5$	0.08	0.42	-
88595	$(NH_4)_2Mg(Sn_7O_{16})$	-	0.04	-
88774	NH_4AlO_2	0.24	0.43	0.25
88843	$(NH_4)_2Zr(Ge_2O_7)$	0.93	0.25	-
90736	$LaNH_4O(CO_3)$	0.29	0.29	-
91313	$(NH_4)_6(UO_2)_5(VO_4)_2O_5$	-	0.61	0.23
91534	$NH_4(Ti_{0.936}Sn_{0.064})O(PO_4)$	0.67	0.67	0.24
91556	$(NH_4)_{0.96}Ti_{0.96}Nb_{0.04}O(PO_4)$	0.67	0.67	0.23
91578	$(NH_4)_{0.8}Ti_{1.733}Li_{0.267}O_4(H_2O)_{0.112}$	0.68	-	0.16
93492	$(NH_4)_2Sr(Ta_2O_7)$	0.19	0.19	-
94465	$(NH_4)_3(Fe_2O_4)$	0.19	0.25	0.25
94466	$(NH_4)_3(Fe_2O_4)$	0.19	0.25	0.25
94467	$(NH_4)_2(Fe_2O_4)$	0.28	0.48	0.28
95260	$(NH_4)_2Sr_{1.5}Ta_3O_{10}$	0.17	0.17	-
95792	$(NH_4)_2La_{0.667}(Ta_2O_7)$	0.20	0.20	-
95959	$(NH_4)_2(ZnP_2O_7)$	0.23	0.23	-
96441	$(NH_4)_2(UO_2)_2(WO_5)O$	0.17	0.17	0.17
96443	$(NH_4)_2(UO_2)(W_2O_8)$	0.30	1.08	-
96739	$(NH_4)_2Te$	0.15	0.15	0.15
97570	$(NH_4)_2((NH_4)_{0.5}Yb_{0.5})(MoO_4)_2$	0.17	0.17	-
98708	$Na_{0.667}(NH_4)_{1.333}(Si_2O_5)$	0.30	0.19	0.30
100224	$Cu_{1.82}(NH_4)_{0.2}(Al_{3.9}Si_{8.1}O_{24})$	0.28	0.28	0.28
100596	$(NH_4)_2V_8O_{16}$	-	-	0.01
108934	$(NH_4)_3(CrO_4)$	0.29	0.29	0.29
150255	$(NH_4)_2Cd(C_2O_4)_2(H_2O)_2$	0.23	0.23	0.23
150292	$(NH_4)_2CuCl_3$	2.49	0.30	2.75
150840	$(NH_4)_2(WO_4)$	0.86	0.22	0.36
150841	$(NH_4)_2((Mo_{0.5}W_{0.5})O_4)$	0.95	0.24	0.36
150842	$(NH_4)_2(MoO_4)$	0.90	0.21	0.26
151882	$NH_4(AlO_2)$	0.22	0.44	0.24
151883	$NH_4(AlO_2)$	0.14	0.14	0.14
152439	$(NH_4)_4Co_7O_{14}$	0.09	0.09	-
153320	$(NH_4)_2(MgSn_7O_{16})$	-	-	0.07
153322	$(NH_4)_2(Fe_2Sn_6O_{16})$	-	-	0.07

153323	$(NH_4)_2(Mn_2Sn_6O_{16})$	-	-	0.07
153459	$NH_4(SbMoO_5)$	2.06	1.30	0.26
154164	$(NH_4)_2(V_8O_{21})$	-	0.10	-
154372	$(NH_4)_4(Fe_2O_5)$	0.62	0.12	0.14
155495	$NH_4(Os_2O_6)$	0.09	0.09	0.09
155528	$NH_4(TiO)(PO_4)$	0.65	0.65	0.23
155564	$(NH_4)_{1.88}Ga_{1.88}Sn_{6.12}O_{16}$	-	-	0.07
155716	$NH_4Mn(HP_3O_{10})$	-	-	0.06
157785	$NH_4(FeO_2)$	0.28	0.46	0.28
157839	$NH_4Ca_2(Nb_3O_{10})$	0.11	0.90	-
158349	$((NH_4)_{0.9}Na_{0.1})Na_{0.1}(Mg_{4.17}Fe_{0.83})(Si_{12}O_{30})$	1.58	1.57	0.27
158352	$((NH_4)_{0.85}Na_{0.15})Na_{0.14}(Mg_{4.18}Fe_{0.82})(Si_{12}O_{30})$	1.44	1.44	0.27
160190	$NH_4Y(P_2O_7)$	2.96	1.80	0.24
160191	$NH_4Lu(P_2O_7)$	2.51	2.00	0.24
160698	$NH_4(Os_2O_6)$	0.07	0.07	0.07
161904	$NH_4Mn(HP_3O_{10})$	-	-	0.06
170786	$(NH_4)_{1.33}Na_{0.67}Si_2O_5$	0.34	0.21	0.66
170832	$(NH_4)_2NbAlO_2((As_{0.8}Nb_{0.2})O_4)_2$	0.45	0.14	0.45
170948	$(NH_4)_6(Cd_4Sn_3Se_{13})$	-	0.26	0.26
171267	$(NH_4)_2(B(AsO_4)_2H)$	0.26	0.26	0.16
172528	$(NH_4)_3Gd_5(PO_4)_6$	-	-	0.29
172618	$NH_4Os_2O_6$	0.06	0.06	0.06
172619	$NH_4Os_2O_6$	0.06	0.06	0.06
172620	$NH_4Os_2O_6$	0.07	0.07	0.07
173150	$(NH_4)_2Ce(PO_4)_2$	0.04	0.04	0.11
173233	$NH_4(Ti_{0.96}Zr_{0.04})OPO_4$	0.66	0.66	0.23
173234	$NH_4(Ti_{0.97}Zr_{0.03})OPO_4$	0.65	0.65	0.23
173235	$NH_4(Ti_{0.97}Zr_{0.03})OPO_4$	0.65	0.65	0.24
200086	$(NH_4)_3AgO_2$	-	0.16	0.17
200100	$(NH_4)_{0.5}(H_2O)_{0.4}NbS_2$	0.02	0.02	-
200101	$(NH_4)_{0.33}(H_2O)_{0.6}TaS_2$	0.02	0.02	-
200161	$(NH_4)_3NdSi_6O_{15}$	0.36	0.37	0.18
200254	NH_4FeO_2	0.28	0.47	0.28
200255	NH_4FeO_2	0.25	0.49	0.27
200310	$(NH_4)_2Ni(MoO_4)_2$	0.27	-	0.47
200388	$(NH_4)_8Zr(MoO_4)_6$	0.56	0.26	0.55
200389	$(NH_4)_8Hf(MoO_4)_6$	0.55	0.25	0.56
200613	$NH_4Fe(PO_4)F$	0.28	1.03	1.03
200777	$(NH_4)_2Ni_4(PO_4)_2(P_2O_7)$	-	2.15	0.14
201060	$(NH_4)_{2.38}Sb_{3.62}Ti_{0.38}O_{11}$	-	0.26	0.26
201788	$(NH_4)_2(SbPO_6)$	0.32	0.29	1.01

201891	$(NH_4)_2NiO_2$	0.16	0.16	-
202071	$(NH_4)_3Li_3TeO_6$	-	0.13	0.39
202073	$(NH_4)_4Na_2(TeO_6)$	0.21	0.24	0.93
202158	$NH_4(TiO)(AsO_4)$	0.51	0.18	0.51
202178	NH_4AuS	1.24	1.24	0.24
202428	$(NH_4)_3Bi_2(VO_4)_3$	-	0.50	0.23
202520	$NH_4LiZn_3O_4$	-	-	0.18
202560	$(NH_4)_4Li(IO_6)$	-	0.27	0.78
202980	$(NH_4)_3(NbAs_2O_9)$	1.12	0.17	1.12
202997	NH_4CuO_2	0.04	-	0.14
203081	NH_4CuO_2	0.05	-	0.15
203214	$(NH_4)_2(Mn_3(OH)_2(VO_4)_2)$	-	0.30	2.31
203218	$NH_4(MoO_2)(AsO_4)$	-	0.28	-
240301	$NH_4La_2Ti_3O_{9.79}$	0.13	0.13	-
240717	$NH_4Yb(P_2O_7)$	2.91	1.88	0.23
240983	$In_{0.459}(NH_4)_{0.917}Nb_{0.541}OPO_4$	0.40	0.40	0.17
245417	$(NH_4)_4(CO_4)$	0.06	0.06	0.06
245420	$(NH_4)_4(CO_4)$	0.08	0.08	-
245421	$(NH_4)_4(CO_4)$	0.05	0.05	0.06
245422	$(NH_4)_4(CO_4)$	0.08	0.08	0.13
245423	$(NH_4)_4(CO_4)$	0.03	0.03	0.03
245424	$(NH_4)_4(CO_4)$	0.04	0.04	0.15
245425	$(NH_4)_4(CO_4)$	0.29	0.29	0.20
245426	$(NH_4)_4(CO_4)$	0.11	0.11	0.29
245428	$(NH_4)_4(CO_4)$	0.56	0.56	0.17
246135	$NH_4Mn_4(PO_4)_3$	0.21	-	-
246141	$NH_4(NbW_2O_9)$	-	-	0.18
246142	$NH_4(TaW_2O_9)$	-	-	0.18
249162	$(NH_4)_2(Bi(PO_4)(WO_4))$	-	0.52	0.16
249327	$NH_4SrNb_2O_6F$	0.06	-	1.07
249730	$((NH_4)_9F_2)(Eu_3Si_{12}O_{32})$	0.29	1.40	1.57
249731	$((NH_4)_9F_2)(Sm_3Si_{12}O_{32})$	0.29	1.41	1.52
250087	$NH_4((Ti_{0.47}Sn_{0.53})O)(PO_4)$	0.66	0.66	0.24
250088	$NH_4((Ti_{0.25}Sn_{0.75})O)(PO_4)$	0.67	0.67	0.22
250224	$NH_4(B_3O_5)$	1.77	0.29	1.77
250381	$(NH_4)_2Yb(PO_4)(MoO_4)$	-	0.73	0.19
250394	$(Na_{0.5}(NH_4)_{0.5})Ti_2(PO_4)_3$	0.21	0.21	0.21
250395	$(Rb_{0.5}(NH_4)_{0.5})Ti_2(PO_4)_3$	0.28	0.28	0.29

260054	$(NH_4)_2Ho(PO_4)(WO_4)$	0.19	0.58	-
280038	$(NH_4)_{1.97}Nb_{1.38}Al_{0.99}As_{1.63}O_{10}$	0.45	0.14	0.45
280310	$(NH_4)_2(Al_2Sb_2O_7)$	0.19	0.19	-
280327	$(NH_4)_2(TiSi_3O_9)(H_2O)$	0.16	0.50	0.49
280900	$(NH_4)_2Mn_2Sr_3(P_2O_7)_3$	-	0.23	0.89
280905	$(NH_4)_3Fe_5(PO_4)_6$	0.10	-	0.69
300177	$(NH_4)_5GaSe_4$	-	0.49	0.22
400850	$(NH_4)_2(TiO)_2(As_{.43}P_{.57}O_4)_2$	0.57	0.57	0.21
402000	$(NH_4)_3(AuSe_2)$	-	0.20	0.14
402642	$(NH_4)_3(SbS_4)$	0.55	0.55	0.27
407293	$(NH_4)_3(BiO_3)$	0.10	0.10	0.10
407357	$(NH_4)_3Al_5(HPO_4)_6(PO_4)_2(H_2O)_{12}$	0.12	0.12	-
408000	$(NH_4)_4(Bi_2O_5)$	0.15	0.46	-
409484	$NH_4(Ag(CO_3))$	-	0.28	0.28
409582	$(NH_4)_3Bi_2(PO_4)_3$	-	0.55	0.19
409792	$Co_{0.25}(NH_4)_{0.75}O_{0.63}$	0.13	0.13	-
411213	$(NH_4)_4(Sb_2O_5)$	0.06	-	0.19
411501	$(NH_4)_3(BSb_4O_{13})$	0.21	0.23	0.67
412135	$(NH_4)_4(GeO_4)$	0.24	0.22	0.17
412393	$(NH_4)_3(BiO_4)$	0.29	0.38	0.41
412528	$(NH_4)_3Lu(Si_2O_7)$	0.22	0.22	2.63
412877	$BaNH_4(FeO_3)$	0.27	3.39	0.30
413149	$NH_4(AsO_2)$	-	0.53	0.25
413432	$(NH_4)_3ScSi_2O_7$	0.21	0.21	3.35
413739	$(NH_4)_6(Cd_4Sn_3Se_{13})$	-	0.26	0.26
415753	$(NH_4)_{17}Fe_5O_{16}$	-	0.19	0.19
415754	$(NH_4)_5FeO_4$	-	0.03	0.16
416427	$(NH_4)_2Bi(PO_4)(MoO_4)$	-	0.59	0.14
416589	$NH_4(NbUO_6)$	-	0.22	-
417102	$SmNH_4PdO_3$	0.63	0.27	-
417103	$EuNH_4PdO_3$	0.62	0.25	-
417104	$PrNH_4PdO_3$	0.61	0.28	-
417105	$GdNH_4PdO_3$	0.63	0.24	-
417106	$PrNH_4(Cu_{0.14}Pd_{0.86})O_3$	0.63	0.29	-
417107	$NdNH_4PdO_3$	0.61	0.27	-
417892	$(NH_4)_2Mn(V_2O_7)$	0.22	0.22	2.07
418108	$(NH_4)_2PbMo_8(P_2O_7)_8$	-	0.28	-
418253	$(NH_4)_3Bi_5(PO_4)_6$	-	-	0.24
623958	$Co(NH_4)_2S_2$	0.97	0.97	0.22
623960	$Co(NH_4)_2S_2$	1.10	1.10	0.25
641321	$(NH_4)_2S$	0.13	0.13	0.13

641376	$(NH_4)_2Te$	0.17	0.17	0.17
660294	$Hg(NH_4)_6S_4$	0.29	0.29	0.23

Table S2. 166 more than four-elements compounds were screened. ID is the ICSD Collection Code, STRU is the chemical formula, C is the specific capacity(mAh/g). a, b and c are respectively the diffusion barrier values(eV) calculated by BVEL on the three axes.

ID	STRU	C	a	b	c
228	$(NH_4)_8SiW_{11}O_{39}(H_2O)_{13}$	80.09	0.25	0.25	0.25
4160	$(NH_4)_4SiW_{12}O_{40}$	37.26	-	0.14	0.61
4188	$NH_4Ga_{0.33}W_{5.66}O_{18}$	19.81	-	-	0.03
4215	$(NH_4)_3SbS_3(Sb_2O_3)_3$	73.46	3.26	3.26	0.08
10363	NH_4NiCrF_6	119.06	0.05	0.05	0.05
10492	$(NH_4)_{0.85}Ti_{0.85}Nb_{1.15}O_5$	99.98	0.08	0.71	-
10497	NH_4TiTaO_5	86.70	0.12	0.31	-
15517	$NH_4Ti_3TaO_9$	57.12	3.49	0.26	-
15518	$(NH_4)_3TiTa_7O_{21}$	48.68	0.26	0.26	-
16557	NH_4TiNbO_5	121.22	0.09	0.65	-
18022	$NH_4Zn(BeF_3)_3$	101.86	3.06	3.06	0.30
18034	NH_4NiAlF_6	133.95	0.12	0.12	0.12
18064	$NH_4Nb(WO_6)$	71.82	0.07	0.07	0.07
23426	$NH_4Cr_{.333}W_{1.667}O_6$	63.78	0.06	0.06	0.06
27596	$NH_4Na_3(Al_4Si_4O_{16})$	49.15	2.89	2.88	0.21
30247	$(NH_4)_2Pb_2Ge_2O_7$	79.73	0.13	0.13	-
30886	$NH_4Cu(PO_4)$	168.48	-	0.14	0.22
30910	$NaNH_4GeO_3$	186.03	-	0.17	2.00
31021	NH_4AgCO_3	159.46	-	0.26	0.26
32023	$(NH_4)_{5.52}Ta_{15.76}O_{42.00}$	41.96	0.20	0.20	3.45
33797	$Rb_2(NH_4)_4((CoO_2)O(CoO_2))$	291.18	0.28	0.28	-
35463	$(NH_4)_4(Mo_8P_{12}O_{52})$	54.34	0.07	1.97	0.07
36380	$(NH_4)_2Na(FeO_3)$	421.87	0.27	0.12	0.53
36612	$(NH_4)_2Na_4(FeO_3)_2$	178.59	2.75	0.30	2.75
39440	NH_4CrPO_4F	161.38	1.00	0.29	1.00
39463	$NH_4SbO(GeO_4)$	97.41	0.56	0.56	0.13
39560	$(NH_4)FeF(PO_4)$	157.58	1.08	1.08	0.28
39585	$NH_4TaO(GeO_4)$	80.21	0.40	0.40	0.13
39697	$(NH_4)_2(Zr_{0.86}Ti_{0.14})(Si_3O_9)(H_2O)$	171.19	0.85	0.85	0.23
39735	NH_4GeOPO_4	145.59	0.91	0.91	0.26
39882	$NH_4(Ge_{0.063}Ti_{0.937}O)(PO_4)$	166.83	0.68	0.68	0.23
39950	$NH_4(Ge_{0.042}Ti_{0.958}O)(PO_4)$	167.38	0.67	0.68	0.23
39951	$NH_4(Ge_{0.184}Ti_{0.816}O)(PO_4)$	163.75	0.69	0.69	0.23
47105	$NH_4Na_2(CuO_2)$	188.65	0.25	0.25	-
48118	$NH_4Na_4(GaO_4)$	118.54	0.38	0.13	2.46
48119	$Cs(NH_4)_4(GaO_4)$	401.33	-	0.06	-
48177	$NH_4Li_2(BO_3)$	366.97	1.72	0.26	1.72
54149	$(NH_4)_{0.97}((Ti_{0.97}Nb_{0.03})O)(PO_4)$	104.88	0.71	0.71	0.26

59281	$(NH_4)_2MgWO_2(PO_4)_2$	124.60	0.53	0.49	0.29
59285	$NH_4(TiO)(PO_4)$	168.48	0.67	0.67	0.24
59887	$(NH_4)_3Bi_2(AsO_4)_3$	96.25	-	0.63	0.26
61388	$NH_4(Sb_2PO_8)$	70.31	-	3.06	0.28
61403	NH_4NaMnO_2	243.54	0.38	0.38	0.27
62064	$Cs(NH_4)_2AuO_2$	148.01	0.63	0.21	0.32
62102	$(NH_4)_9Li_3(IO_6)_2O$	623.00	0.50	0.50	0.10
62130	$(NH_4)_2Nb_{10}W_7O_{47}$	18.04	-	0.14	-
62136	$(NH_4)_2Na_4(GaO_3)_2$	163.35	2.84	0.29	2.84
62166	$(NH_4)_3(Sb_3P_2O_{14})$	123.26	0.04	0.04	-
62287	$NH_4Mn_4(PO_4)_3$	53.05	-	-	0.21
62650	$Na_4(NH_4)_2(Ge_2O_7)$	153.08	0.22	0.26	3.08
63544	$NH_4Ni(AsO_4)$	135.30	0.17	0.17	-
63563	$(NH_4)_{1\cdot88}Nb(WO_6)$	135.02	0.01	0.01	0.01
65260	$NH_4Li_4(AlO_4)$	273.36	0.15	-	-
65291	$(NH_4)_3(LiSiO_4)$	811.79	-	0.27	0.34
65420	$NH_4(Mo_2P_3O_{13})$	54.34	2.05	0.06	0.06
65656	$NH_4Mo(P_2O_7)$	99.22	2.77	2.13	0.28
65682	$NH_4MoW(P_3O_{12})$	47.41	-	0.25	1.68
65942	$Cs_2(NH_4)_4(Fe_2O_5)$	329.71	0.24	-	0.29
65976	$(NH_4)_{11}(Li(MnO_4)_4)$	610.10	0.30	0.30	0.47
65978	$(NH_4)_3Na_2(LiTeO_6)$	290.13	0.21	-	0.11
67120	$(NH_4)_{.84}(Nb_{.08}Ti_{.92})OPO_4$	138.39	0.66	0.66	0.22
68496	$(NH_4)_{0\cdot75}MoNbO(PO_4)(P_2O_7)$	42.39	-	0.25	1.60
68706	$NH_4SnO(PO_4)$	116.47	0.78	0.78	0.22
69429	$NH_4(SbO)(SiO_4)$	116.47	0.72	0.72	0.17
69556	$NH_4VO(PO_4)$	165.36	0.67	0.25	0.67
71536	$(NH_4)_2Co(SeO_3)_2$	171.18	0.10	0.10	-
71540	$(NH_4)_2Mn(SeO_3)_2$	173.39	0.09	0.09	-
71904	$NH_4(TiO)((P_{0\cdot58}As_{0\cdot42})O_4)$	150.94	0.58	0.58	0.22
71927	$(NH_4)_6Cr_{0\cdot38}Nb_{15\cdot62}O_{42}$	74.95	0.26	0.26	-
72051	$NH_4(TiO)((P_{0\cdot5}As_{0\cdot5})O_4)$	148.01	0.59	0.21	0.59
72720	$NH_4(Ti_{.496}Sn_{.504})O(PO_4)$	137.53	0.65	0.24	0.65
72873	$(NH_4)_3(NbP_2O_9)$	268.79	0.23	0.93	0.93
73136	$Rb_4(NH_4)_2(Fe_2O_5)$	100.71	0.27	0.57	0.73
73205	$(NH_4)_5Li(Ge_2O_7)$	505.45	0.26	0.53	0.26
73260	$(NH_4)_6CrNb_{15}O_{42}$	75.85	0.27	0.27	-
74300	$(Rb_{0\cdot88}(NH_4)_{0\cdot12})_2((NH_4)_{0\cdot91}Rb_{0\cdot09})_2(GeO_3)_2$	135.62	-	0.29	1.69
74591	$NH_4(TiO)(AsO_4)$	131.97	0.51	0.51	0.19
74955	$Cs(NH_4)_4(TlO_4)$	267.22	-	0.09	-
74956	$(NH_4)_4Na_2(Tl_2O_6)$	194.83	0.28	0.07	0.97
76891	$(NH_4)_2Pb(SO_4)_2$	134.28	0.09	0.09	-
79650	$NH_4(SnO)(PO_4)$	116.47	0.67	0.67	0.22

79651	$NH_4(VO)(PO_4)$	165.36	0.65	0.65	0.24
79702	$(NH_4)_2Ni(WO_2(PO_4)_2)$	123.45	0.44	0.44	0.19
79711	$NH_4(AlAs_2O_7)$	92.70	1.63	-	0.23
80022	$NH_4(TiO)((P_{0.75}As_{0.25})O_4)$	157.58	0.61	0.61	0.22
80023	$NH_4(TiO)((P_{0.56}As_{0.44})O_4)$	150.20	0.58	0.58	0.22
80024	$NH_4(TiO)((P_{0.38}As_{0.62})O_4)$	143.81	0.55	0.55	0.20
80893	$NH_4GaF(PO_4)$	145.59	1.17	1.17	0.30
80976	$NH_4(SnO)(AsO_4)$	97.77	0.52	0.52	0.18
81478	$Cs_2(NH_4)_2B_{10}O_{17}$	82.68	-	0.27	1.18
81479	$(NH_4)_6(VO)_4(SO_4)_8$	155.15	0.62	3.01	0.07
82391	$NH_4(GaAs_2O_7)$	80.69	2.02	-	0.22
82398	$(NH_4)_2(Be_2Si_3O_9)$	217.80	0.77	0.28	0.77
82457	$NH_4Li(Si_2O_5)$	187.34	3.17	0.28	-
82601	$(NH_4)_{0.998}((Ti_{0.998}W_{0.002})O)(PO_4)$	167.86	0.67	0.24	0.67
83431	$(NH_4)_2Ca_2(CO_3)_3$	206.07	0.47	0.47	0.29
85404	$(NH_4)_6(CuSi_2O_8)$	648.12	0.18	0.23	0.43
85505	$(NH_4)_2Ca(NO_2)_4$	239.19	0.12	0.12	-
85583	$(NH_4)_2Au_4CdS_4$	52.12	0.81	0.27	1.66
85750	$(NH_4)_3Al_5(HPO_4)_6(PO_4)_2(H_2O)_{18}$	65.61	0.12	0.12	-
86784	$NH_4Fe_{0.43}Nb_{1.57}O_5$	107.12	0.08	0.42	-
88595	$(NH_4)_2Mg(Sn_7O_{16})$	48.14	-	0.04	-
88843	$(NH_4)_2Zr(Ge_2O_7)$	153.52	0.93	0.25	-
91534	$NH_4(Ti_{0.936}Sn_{0.064})O(PO_4)$	163.80	0.67	0.67	0.24
91556	$(NH_4)_{0.96}Ti_{0.96}Nb_{0.04}O(PO_4)$	159.93	0.67	0.67	0.23
91578	$(NH_4)_{0.8}Ti_{1.733}Li_{0.267}O_4(H_2O)_{0.112}$	143.78	0.68	-	0.16
93492	$(NH_4)_2Sr(Ta_2O_7)$	95.33	0.19	0.19	-
95260	$(NH_4)_2Sr_{1.5}Ta_3O_{10}$	64.17	0.17	0.17	-
95959	$(NH_4)_2(ZnP_2O_7)$	224.18	0.23	0.23	-
98708	$Na_{0.667}(NH_4)_{1.333}(Si_2O_5)$	235.96	0.30	0.19	0.30
100224	$Cu_{1.82}(NH_4)_{0.2}(Al_{3.9}Si_{8.1}O_{24})$	6.44	0.28	0.28	0.28
150255	$(NH_4)_2Cd(C_2O_4)_2(H_2O)_2$	186.03	0.23	0.23	0.23
150841	$(NH_4)_2((Mo_{0.5}W_{0.5})O_4)$	262.64	0.95	0.24	0.36
153320	$(NH_4)_2(MgSn_7O_{16})$	48.14	-	-	0.07
153322	$(NH_4)_2(Fe_2Sn_6O_{16})$	49.52	-	-	0.07
153323	$(NH_4)_2(Mn_2Sn_6O_{16})$	49.61	-	-	0.07
153459	$NH_4(SbMoO_5)$	89.90	2.06	1.30	0.26
155564	$(NH_4)_{1.88}Ga_{1.88}Sn_{6.12}O_{16}$	45.13	-	-	0.07
157839	$NH_4Ca_2(Nb_3O_{10})$	51.62	0.11	0.90	-
158349	$((NH_4)_{0.9}Na_{0.1})Na_{0.1}(Mg_{4.17}Fe_{0.83})(Si_{12}O_{30})$	24.93	1.58	1.57	0.27
158352	$((NH_4)_{0.85}Na_{0.15})Na_{0.14}(Mg_{4.18}F$	23.50	1.44	1.44	0.27

	$e_{0.82})(Si_{12}O_{30})$				
161904	$NH_4Mn(HP_3O_{10})$	86.70	-	-	0.06
170786	$(NH_4)_{1.33}Na_{0.67}Si_2O_5$	235.32	0.34	0.21	0.66
170832	$(NH_4)_2NbAlO_2((As_{0.8}Nb_{0.2})O_4)_2$	122.55	0.45	0.14	0.45
171267	$(NH_4)_2(B(AsO_4)_2H)$	184.75	0.26	0.26	0.16
173233	$NH_4(Ti_{0.96}Zr_{0.04})OPO_4$	166.68	0.66	0.66	0.23
173235	$NH_4(Ti_{0.97}Zr_{0.03})OPO_4$	167.13	0.65	0.65	0.24
200310	$(NH_4)_2Ni(MoO_4)_2$	141.37	0.27	-	0.47
200388	$(NH_4)_8Zr(MoO_4)_6$	203.91	0.56	0.26	0.55
200389	$(NH_4)_8Hf(MoO_4)_6$	188.32	0.55	0.25	0.56
200613	$NH_4Fe(PO_4)F$	157.58	0.28	1.03	1.03
200777	$(NH_4)_2Ni_4(PO_4)_2(P_2O_7)$	89.30	-	2.15	0.14
201060	$(NH_4)_{2.38}Sb_{3.62}Ti_{0.38}O_{11}$	100.27	-	0.26	0.26
201788	$(NH_4)_2(SbPO_6)$	215.17	0.32	0.29	1.01
202071	$(NH_4)_3Li_3TeO_6$	328.03	-	0.13	0.39
202073	$(NH_4)_4Na_2(TeO_6)$	396.87	0.21	0.24	0.93
202428	$(NH_4)_3Bi_2(VO_4)_3$	105.33	-	0.50	0.23
202520	$NH_4LiZn_3O_4$	100.71	-	-	0.18
202560	$(NH_4)_4Li(IO_6)$	465.90	-	0.27	0.78
202980	$(NH_4)_3(NbAs_2O_9)$	207.67	1.12	0.17	1.12
203214	$(NH_4)_2(Mn_3(OH)_2(VO_4)_2)$	124.89	-	0.30	2.31
203218	$NH_4(MoO_2)(AsO_4)$	100.33	-	0.28	-
246141	$NH_4(NbW_2O_9)$	44.28	-	-	0.18
246142	$NH_4(TaW_2O_9)$	38.66	-	-	0.18
249162	$(NH_4)_2(Bi(PO_4)(WO_4))$	97.06	-	0.52	0.16
249327	$NH_4SrNb_2O_6F$	68.87	0.06	-	1.07
250087	$NH_4((Ti_{0.47}Sn_{0.53})O)(PO_4)$	136.24	0.66	0.66	0.24
250088	$NH_4((Ti_{0.25}Sn_{0.75})O)(PO_4)$	126.21	0.67	0.67	0.22
250394	$(Na_{0.5}(NH_4)_{0.5})Ti_2(PO_4)_3$	34.13	0.21	0.21	0.21
250395	$(Rb_{0.5}(NH_4)_{0.5})Ti_2(PO_4)_3$	31.63	0.28	0.28	0.29
280038	$(NH_4)_{1.97}Nb_{1.38}Al_{0.99}As_{1.63}O_{10}$	120.68	0.45	0.14	0.45
280310	$(NH_4)_2(Al_2Sb_2O_7)$	130.68	0.19	0.19	-
280327	$(NH_4)_2(TiSi_3O_9)(H_2O)$	194.12	0.16	0.50	0.49
280900	$(NH_4)_2Mn_2Sr_3(P_2O_7)_3$	59.80	-	0.23	0.89
280905	$(NH_4)_3Fe_5(PO_4)_6$	94.55	0.10	-	0.69
400850	$(NH_4)_2(TiO)_2(As_{.43}P_{.57}O_4)_2$	150.57	0.57	0.57	0.21
407357	$(NH_4)_3Al_5(HPO_4)_6(PO_4)_2(H_2O)_{12}$	89.20	0.12	0.12	-
409484	$NH_4(Ag(CO_3))$	159.46	-	0.28	0.28
409582	$(NH_4)_3Bi_2(PO_4)_3$	114.32	-	0.55	0.19
411501	$(NH_4)_3(BSb_4O_{13})$	113.67	0.21	0.23	0.67
412877	$BaNH_4(FeO_3)$	111.16	0.27	3.39	0.30
413739	$(NH_4)_6(Cd_4Sn_3Se_{13})$	87.74	-	0.26	0.26
416427	$(NH_4)_2Bi(PO_4)(MoO_4)$	115.47	-	0.59	0.14

417892	$(NH_4)_2Mn(V_2O_7)$	199.17	0.22	0.22	2.07
418108	$(NH_4)_2PbMo_8(P_2O_7)_8$	22.64	-	0.28	-
418253	$(NH_4)_3Bi_5(PO_4)_6$	49.76	-	-	0.24

Table S3. 108 compounds were obtained using a filter with a specific capacity greater than 100 mAh/g. ID is the ICSD Collection Code, STRU is the chemical formula, C iand s the specific capacity (mAh/g). a, b and c are respectively the diffusion barrier values(eV) calculated by BVEL on the three axes.

ID	STRU	C	a	b	c
10363	NH_4NiCrF_6	119.06	0.05	0.05	0.05
16557	NH_4TiNbO_5	121.22	0.09	0.65	-
18022	$NH_4Zn(BeF_3)_3$	101.86	3.06	3.06	0.30
18034	NH_4NiAlF_6	133.95	0.12	0.12	0.12
30886	$NH_4Cu(PO_4)$	168.48	-	0.14	0.22
30910	$NaNH_4GeO_3$	186.03	-	0.17	2.00
31021	NH_4AgCO_3	159.46	-	0.26	0.26
33797	$Rb_2(NH_4)_4((CoO_2)O(CoO_2))$	291.18	0.28	0.28	-
36380	$(NH_4)_2Na(FeO_3)$	421.87	0.27	0.12	0.53
36612	$(NH_4)_2Na_4(FeO_3)_2$	178.59	2.75	0.30	2.75
39440	NH_4CrPO_4F	161.38	1.00	0.29	1.00
39560	$NH_4FeF(PO_4)$	157.58	1.08	1.08	0.28
39697	$(NH_4)_2(Zr_{0\cdot86}Ti_{0\cdot14})(Si_3O_9)(H_2O)$	171.19	0.85	0.85	0.23
39735	NH_4GeOPO_4	145.59	0.91	0.91	0.26
39882	$NH_4(Ge_{0\cdot063}Ti_{0\cdot937}O)(PO_4)$	166.83	0.68	0.68	0.23
39950	$NH_4(Ge_{0\cdot042}Ti_{0\cdot958}O)(PO_4)$	167.38	0.67	0.68	0.23
39951	$NH_4(Ge_{0\cdot184}Ti_{0\cdot816}O)(PO_4)$	163.75	0.69	0.69	0.23
47105	$NH_4Na_2(CuO_2)$	188.65	0.25	0.25	-
48118	$NH_4Na_4(GaO_4)$	118.54	0.38	0.13	2.46
48119	$Cs(NH_4)_4(GaO_4)$	401.33	-	0.06	-
48177	$NH_4Li_2(BO_3)$	366.97	1.72	0.26	1.72
54149	$(NH_4)_{0\cdot97}((Ti_{0\cdot97}Nb_{0\cdot03})O)(PO_4)$	104.88	0.71	0.71	0.26
59281	$(NH_4)_2MgWO_2(PO_4)_2$	124.60	0.53	0.49	0.29
59285	$NH_4(TiO)(PO_4)$	168.48	0.67	0.67	0.24
61403	NH_4NaMnO_2	243.54	0.38	0.38	0.27
62064	$Cs(NH_4)_2AuO_2$	148.01	0.63	0.21	0.32
62102	$(NH_4)_9Li_3(IO_6)_2O$	623.00	0.50	0.50	0.10
62136	$(NH_4)_2Na_4(GaO_3)_2$	163.35	2.84	0.29	2.84
62166	$(NH_4)_3(Sb_3P_2O_{14})$	123.26	0.04	0.04	-
62650	$Na_4(NH_4)_2(Ge_2O_7)$	153.08	0.22	0.26	3.08

63544	$NH_4Ni(AsO_4)$	135.30	0.17	0.17	-
63563	$(NH_4)_{1.88}Nb(WO_6)$	135.02	0.01	0.01	0.01
65260	$NH_4Li_4(AlO_4)$	273.36	0.15	-	-
65291	$(NH_4)_3(LiSiO_4)$	811.79	-	0.27	0.34
65942	$Cs_2(NH_4)_4(Fe_2O_5)$	329.71	0.24	-	0.29
65976	$(NH_4)_{11}(Li(MnO_4)_4)$	610.10	0.30	0.30	0.47
65978	$(NH_4)_3Na_2(LiTeO_6)$	290.13	0.21	-	0.11
67120	$(NH_4)_{.84}(Nb_{.08}Ti_{.92})OPO_4$	138.39	0.66	0.66	0.22
68706	$NH_4SnO(PO_4)$	116.47	0.78	0.78	0.22
69429	$NH_4(SbO)(SiO_4)$	116.47	0.72	0.72	0.17
69556	$NH_4VO(PO_4)$	165.36	0.67	0.25	0.67
71536	$(NH_4)_2Co(SeO_3)_2$	171.18	0.10	0.10	-
71540	$(NH_4)_2Mn(SeO_3)_2$	173.39	0.09	0.09	-
71904	$NH_4(TiO)((P_{0.58}As_{0.42})O_4)$	150.94	0.58	0.58	0.22
72051	$NH_4(TiO)((P_{0.5}As_{0.5})O_4)$	148.01	0.59	0.21	0.59
72720	$NH_4(Ti_{.496}Sn_{.504})O(PO_4)$	137.53	0.65	0.24	0.65
72873	$(NH_4)_3(NbP_2O_9)$	268.79	0.23	0.93	0.93
73136	$Rb_4(NH_4)_2(Fe_2O_5)$	100.71	0.27	0.57	0.73
73205	$(NH_4)_5Li(Ge_2O_7)$	505.45	0.26	0.53	0.26
74300	$(Rb_{0.88}(NH_4)_{0.12})_2((NH_4)_{0.91}Rb_{0.09})_2(GeO_3)_2$	135.62	-	0.29	1.69
74591	$NH_4(TiO)(AsO_4)$	131.97	0.51	0.51	0.19
74955	$Cs(NH_4)_4(TlO_4)$	267.22	-	0.09	-
74956	$(NH_4)_4Na_2(Tl_2O_6)$	194.83	0.28	0.07	0.97
76891	$(NH_4)_2Pb(SO_4)_2$	134.28	0.09	0.09	-
79650	$NH_4(SnO)(PO_4)$	116.47	0.67	0.67	0.22
79651	$NH_4(VO)(PO_4)$	165.36	0.65	0.65	0.24
79702	$(NH_4)_2Ni(WO_2(PO_4)_2)$	123.45	0.44	0.44	0.19
80022	$NH_4(TiO)((P_{0.75}As_{0.25})O_4)$	157.58	0.61	0.61	0.22
80023	$NH_4(TiO)((P_{0.56}As_{0.44})O_4)$	150.20	0.58	0.58	0.22
80024	$NH_4(TiO)((P_{0.38}As_{0.62})O_4)$	143.81	0.55	0.55	0.20
80893	$NH_4GaF(PO_4)$	145.59	1.17	1.17	0.30
81479	$(NH_4)_6(VO)_4(SO_4)_8$	155.15	0.62	3.01	0.07
82398	$(NH_4)_2(Be_2Si_3O_9)$	217.80	0.77	0.28	0.77
82457	$NH_4Li(Si_2O_5)$	187.34	3.17	0.28	-
82601	$(NH_4)_{0.998}((Ti_{0.998}W_{0.002})O)(PO_4)$	167.86	0.67	0.24	0.67
83431	$(NH_4)_2Ca_2(CO_3)_3$	206.07	0.47	0.47	0.29
85404	$(NH_4)_6(CuSi_2O_8)$	648.12	0.18	0.23	0.43
85505	$(NH_4)_2Ca(NO_2)_4$	239.19	0.12	0.12	-

86784	$NH_4Fe_{0.43}Nb_{1.57}O_5$	107.12	0.08	0.42	-
88843	$(NH_4)_2Zr(Ge_2O_7)$	153.52	0.93	0.25	-
91534	$NH_4(Ti_{0.936}Sn_{0.064})O(PO_4)$	163.80	0.67	0.67	0.24
91556	$(NH_4)_{0.96}Ti_{0.96}Nb_{0.04}O(PO_4)$	159.93	0.67	0.67	0.23
91578	$(NH_4)_{0.8}Ti_{1.733}Li_{0.267}O_4(H_2O)_{0.112}$	143.78	0.68	-	0.16
95959	$(NH_4)_2(ZnP_2O_7)$	224.18	0.23	0.23	-
98708	$Na_{0.667}(NH_4)_{1.333}(Si_2O_5)$	235.96	0.30	0.19	0.30
150255	$(NH_4)_2Cd(C_2O_4)_2(H_2O)_2$	186.03	0.23	0.23	0.23
150841	$(NH_4)_2((Mo_{0.5}W_{0.5})O_4)$	262.64	0.95	0.24	0.36
170786	$(NH_4)_{1.33}Na_{0.67}Si_2O_5$	235.32	0.34	0.21	0.66
170832	$(NH_4)_2NbAlO_2((As_{0.8}Nb_{0.2})O_4)_2$	122.55	0.45	0.14	0.45
171267	$(NH_4)_2(B(AsO_4)_2H)$	184.75	0.26	0.26	0.16
173233	$NH_4(Ti_{0.96}Zr_{0.04})OPO_4$	166.68	0.66	0.66	0.23
173235	$NH_4(Ti_{0.97}Zr_{0.03})OPO_4$	167.13	0.65	0.65	0.24
200310	$(NH_4)_2Ni(MoO_4)_2$	141.37	0.27	-	0.47
200388	$(NH_4)_8Zr(MoO_4)_6$	203.91	0.56	0.26	0.55
200389	$(NH_4)_8Hf(MoO_4)_6$	188.32	0.55	0.25	0.56
200613	$NH_4Fe(PO_4)F$	157.58	0.28	1.03	1.03
201060	$(NH_4)_{2.38}Sb_{3.62}Ti_{0.38}O_{11}$	100.27	-	0.26	0.26
201788	$(NH_4)_2(SbPO_6)$	215.17	0.32	0.29	1.01
202071	$(NH_4)_3Li_3TeO_6$	328.03	-	0.13	0.39
202073	$(NH_4)_4Na_2(TeO_6)$	396.87	0.21	0.24	0.93
202428	$(NH_4)_3Bi_2(VO_4)_3$	105.33	-	0.50	0.23
202520	$NH_4LiZn_3O_4$	100.71	-	-	0.18
202560	$(NH_4)_4Li(IO_6)$	465.90	-	0.27	0.78
202980	$(NH_4)_3(NbAs_2O_9)$	207.67	1.12	0.17	1.12
203214	$(NH_4)_2(Mn_3(OH)_2(VO_4)_2)$	124.89	-	0.30	2.31
203218	$NH_4(MoO_2)(AsO_4)$	100.33	-	0.28	-
250087	$NH_4((Ti_{0.47}Sn_{0.53})O)(PO_4)$	136.24	0.66	0.66	0.24
250088	$NH_4((Ti_{0.25}Sn_{0.75})O)(PO_4)$	126.21	0.67	0.67	0.22
280038	$(NH_4)_{1.97}Nb_{1.38}Al_{0.99}As_{1.63}O_{10}$	120.68	0.45	0.14	0.45
280310	$(NH_4)_2(Al_2Sb_2O_7)$	130.68	0.19	0.19	-
280327	$(NH_4)_2(TiSi_3O_9)(H_2O)$	194.12	0.16	0.50	0.49
400850	$(NH_4)_2(TiO)_2(As_{.43}P_{.57}O_4)_2$	150.57	0.57	0.57	0.21
409484	$NH_4(Ag(CO_3))$	159.46	-	0.28	0.28

409582	$(NH_4)_3Bi_2(PO_4)_3$	114.32	-	0.55	0.19
411501	$(NH_4)_3(BSb_4O_{13})$	113.67	0.21	0.23	0.67
412877	$BaNH_4(FeO_3)$	111.16	0.27	3.39	0.30
416427	$(NH_4)_2Bi(PO_4)(MoO_4)$	115.47	-	0.59	0.14
417892	$(NH_4)_2Mn(V_2O_7)$	199.17	0.22	0.22	2.07

Table S4. The final 72 compounds (Original structure containing K) were screened by using the condition that the open circuit voltage (OCV) was greater than 3 V. ICSD is the ICSD Collection Code, STRU is the chemical formula. The a, b, c (Å) and α, β, γ (°) are unit cell parameters. V ($Å^3$) is the cell volume.

ID	STRU	a	b	c	α	β	γ	V
16557	$KTiNbO_5$	6.46	3.79	18.47	90.00	90.00	90.00	452.43
18022	$KZn(BeF_3)_3$	6.58	6.58	9.62	90.00	90.00	120.00	361.15
30886	$KCu(PO_4)$	17.94	6.74	6.80	90.00	90.00	90.00	821.87
30910	$NaKGeO_3$	10.68	6.90	4.80	90.00	90.00	90.00	353.52
31021	$KAgCO_3$	20.50	5.82	6.00	90.00	90.00	90.00	715.86
36380	$K_2Na(FeO_3)$	11.01	6.20	6.71	90.00	90.00	90.00	457.63
36612	$K_2Na_4(FeO_3)_2$	6.51	6.20	10.21	90.00	90.00	90.00	411.38
39440	$KCrPO_4F$	6.35	10.56	12.78	90.00	90.00	90.00	855.76
39697	K2(Zr0.86Ti0.14)(Si3O9)	10.21	13.24	7.17	90.00	90.00	90.00	969.57
39735	$KGeOPO_4$	12.60	6.31	10.00	90.00	90.00	90.00	794.77
48118	$KNa_4(GaO_4)$	10.46	5.96	18.71	90.00	90.00	90.00	1167.17
48119	$CsK_4(GaO_4)$	11.55	6.68	20.97	90.00	90.00	90.00	1616.46
48177	$KLi_2(BO_3)$	7.97	6.43	6.46	90.00	90.00	90.00	331.05
59281	$K_2MgWO_2(PO_4)_2$	9.14	9.16	10.74	90.28	90.52	90.11	898.89
59285	$K(TiO)(PO_4)$	12.80	6.39	10.59	90.00	90.00	90.00	866.17
62064	CsK_2AuO_2	12.57	7.27	6.28	90.00	90.00	90.00	573.81
62102	$K_9Li_3(IO_6)_2O$	9.55	9.55	11.72	90.00	90.00	120.00	925.65
62136	$K_2Na_4(GaO_3)_2$	6.52	6.13	10.25	90.00	90.00	90.00	409.29
63544	$KNi(AsO_4)$	4.97	4.97	28.53	90.00	90.00	120.00	610.73
65260	$KLi_4(AlO_4)$	5.54	15.44	11.03	90.00	109.71	90.00	889.14
65291	$K_3(LiSiO_4)$	11.33	9.74	11.77	90.00	121.30	90.00	1109.50
65942	$Cs_2K_4(Fe_2O_5)$	7.07	11.39	7.00	90.00	91.76	90.00	563.25
65976	$K_{11}(Li(MnO_4)_4)$	7.87	7.87	17.51	90.00	90.00	90.00	1084.95
65978	$K_3Na_2(LiTeO_6)$	9.28	11.87	6.79	90.00	93.80	90.00	746.46
68706	$KSnO(PO_4)$	13.15	6.53	10.38	90.00	90.00	90.00	890.27
69429	$K(SbO)(SiO_4)$	13.01	6.47	10.61	90.00	90.00	90.00	893.75
69556	$KVO(PO_4)$	12.76	10.52	6.36	90.00	90.00	90.00	854.27
71536	$K_2Co(SeO_3)_2$	5.52	5.52	18.52	90.00	90.00	120.00	488.00
71540	$K_2Mn(SeO_3)_2$	5.62	5.62	18.67	90.00	90.00	120.00	509.69
72873	$K_3(NbP_2O_9)$	5.21	17.70	9.75	90.00	90.48	90.00	899.61
73205	$K_5Li(Ge_2O_7)$	6.25	15.87	10.58	90.00	109.38	90.00	989.82
74591	$K(TiO)(AsO_4)$	13.12	6.57	10.78	90.00	90.00	90.00	928.52
74955	$CsK_4(TlO_4)$	11.92	6.86	21.44	90.00	90.00	90.00	1752.15
74956	$K_4Na_2(Tl_2O_6)$	6.41	6.91	11.88	90.00	95.69	90.00	524.17

76891	$K_2Pb(SO_4)_2$	5.59	5.59	20.69	90.00	90.00	120.00	559.40
79650	$K(SnO)(PO_4)$	13.17	6.54	10.75	90.00	90.00	90.00	925.30
79651	$KVO(PO_4)$	12.82	6.39	10.56	90.00	90.00	90.00	864.21
79702	$K_2Ni(WO_2(PO_4)_2)$	9.16	9.16	10.68	90.00	90.00	90.00	896.14
80023	$K(TiO)((P_{0.56}As_{0.44})O_4)$	12.95	6.48	10.66	90.00	90.00	90.00	894.19
80893	$KGaF(PO_4)$	12.72	6.30	10.43	90.00	90.00	90.00	835.98
81479	$K_6(VO)_4(SO_4)_8$	8.93	18.30	9.97	90.00	90.11	90.00	1629.90
82398	$K_2(Be_2Si_3O_9)$	10.26	6.94	12.18	90.00	106.76	90.00	829.99
82457	$KLi(Si_2O_5)$	5.98	4.80	8.16	90.00	93.48	90.00	233.78
83431	$K_2Ca_2(CO_3)_3$	13.01	13.01	8.62	90.00	90.00	120.00	1262.82
85404	$K_6(CuSi_2O_8)$	6.19	6.66	7.53	83.66	87.71	70.19	290.24
85505	$K_2Ca(NO_2)_4$	9.45	5.45	10.91	90.00	88.57	90.00	561.82
88843	$K_2Zr(Ge_2O_7)$	9.96	5.56	12.96	90.00	105.17	90.00	692.31
91534	$K(Ti_{0.936}Sn_{0.064})O(PO_4)$	12.83	6.41	10.58	90.00	90.00	90.00	870.50
95959	$K_2(ZnP_2O_7)$	7.86	7.86	11.32	90.00	90.00	90.00	699.11
98708	$Na_{0.667}K_{1.333}(Si_2O_5)$	12.73	7.32	17.83	90.00	100.85	90.00	1631.84
150841	$K_2((Mo_{0.5}W_{0.5})O_4)$	12.38	6.11	7.55	90.00	115.80	90.00	514.28
170786	$K_{1.33}Na_{0.67}Si_2O_5$	19.68	7.27	12.57	90.00	117.82	90.00	1589.86
200310	$K_2Ni(MoO_4)_2$	8.47	19.04	10.84	90.00	90.00	90.00	1747.49
200388	$K_8Zr(MoO_4)_6$	10.20	11.85	13.12	90.00	119.00	90.00	1386.98
200389	$K_8Hf(MoO_4)_6$	10.18	11.77	13.10	90.00	118.82	90.00	1374.86
200613	$KFe(PO_4)F$	10.66	12.89	6.37	90.00	90.00	90.00	874.62
201788	$K_2(SbPO_6)$	9.43	5.89	11.03	90.00	90.00	90.00	612.67
202071	$K_3Li_3TeO_6$	11.70	5.99	9.69	90.00	90.00	90.00	678.40
202073	$K_4Na_2(TeO_6)$	7.26	9.83	6.67	90.00	122.74	90.00	399.93
202428	$K_3Bi_2(VO_4)_3$	13.96	13.86	7.10	90.00	112.80	90.00	1265.06
202560	$K_4Li(IO_6)$	13.12	9.98	11.19	90.00	90.00	90.00	1465.58
202980	$K_3(NbAs_2O_9)$	22.39	5.44	7.87	90.00	90.00	90.00	957.83
203214	$K_2(Mn_3(OH)_2(VO_4)_2)$	15.20	6.16	5.40	90.00	105.40	90.00	487.51
203218	$K(MoO_2)(AsO_4)$	10.68	6.62	6.98	90.00	90.00	90.00	493.16
280310	$K_2(Al_2Sb_2O_7)$	5.63	5.63	8.05	90.00	90.00	120.00	221.03
280327	$K_2(TiSi_3O_9)(H_2O)$	7.15	9.94	12.95	90.00	90.00	90.00	920.71
400850	$K_2(TiO)_2(As_{.43}P_{.57}O_4)_2$	12.95	6.48	10.68	90.00	90.00	90.00	895.69
409484	$K(Ag(CO_3))$	20.39	5.78	5.94	90.00	90.00	90.00	699.89
409582	$K_3Bi_2(PO_4)_3$	13.83	13.48	6.81	90.00	114.94	90.00	1150.86
411501	$K_3(BSb_4O_{13})$	7.13	7.23	13.26	82.00	99.77	117.08	598.67

412877	$BaK(FeO_3)$	5.80	11.53	12.78	90.00	90.00	90.00	854.82
417892	$K_2Mn(V_2O_7)$	8.61	8.61	5.54	90.00	90.00	90.00	410.45

Table S5. The 72 compounds (Optimized structure containing NH_4^+) were screened by using the condition that the open circuit voltage (OCV) was greater than 3 V. ICSD is the ICSD Collection Code, STRU is the Struct formula. The a', b', c'(Å) and α', β', γ'(°) are unit cell parameters. V'(Å^3) is the cell volume.

ICSD	STRU	a'	b'	c'	α'	β'	γ'	V'
16557	NH_4TiNbO_5	6.61	3.83	19.09	90.00	90.00	90.02	483.36
18022	$NH_4Zn(BeF_3)_3$	6.78	6.78	9.80	90.00	90.00	120.01	390.72
30886	$NH_4Cu(PO_4)$	6.65	7.64	18.47	90.00	90.00	90.00	938.31
30910	$NaNH_4GeO_3$	4.88	7.04	11.04	90.00	90.00	89.93	378.93
31021	NH_4AgCO_3	6.19	6.13	11.44	105.54	105.68	90.00	401.66
36380	$(NH_4)_2Na(FeO_3)$	6.43	6.72	7.18	90.00	90.00	119.62	269.91
36612	$(NH_4)_2Na_4(FeO_3)_2$	6.38	7.32	10.27	90.00	90.00	86.81	478.55
39440	NH_4CrPO_4F	6.49	10.76	13.04	90.00	90.00	90.00	910.87
39697	$(NH_4)_2(Zr_{0.86}Ti_{0.14})(Si_3O_9)(H_2O)$	10.64	13.47	7.40	89.10	90.62	89.63	1060.22
39735	NH_4GeOPO_4	6.55	10.15	12.84	89.70	89.80	89.89	853.99
48118	$NH_4Na_4(GaO_4)$	5.80	11.34	19.66	90.00	90.00	90.00	1293.73
48119	$Cs(NH_4)_4(GaO_4)$	7.32	12.44	24.21	90.00	90.00	88.90	2201.95
48177	$NH_4Li_2(BO_3)$	6.44	6.50	8.52	90.00	90.12	90.00	356.73
59281	$(NH_4)_2MgWO_2(PO_4)_2$	9.43	9.45	10.87	89.66	90.53	89.80	968.91
59285	$NH_4(TiO)(PO_4)$	6.58	10.71	13.05	89.85	90.06	89.97	919.61
62064	$Cs(NH_4)_2AuO_2$	7.12	7.74	13.55	87.18	90.00	90.00	745.12
62102	$(NH_4)_9Li_3(IO_6)_2O$	9.82	10.14	12.93	90.00	90.00	121.23	1101.02
62136	$(NH_4)_2Na_4(GaO_3)_2$	6.17	7.31	10.36	90.00	90.00	87.41	466.72
63544	$NH_4Ni(AsO_4)$	5.04	5.04	10.63	76.31	76.29	60.02	225.14
65260	$NH_4Li_4(AlO_4)$	5.60	10.66	16.47	90.01	90.00	100.22	968.35
65291	$(NH_4)_3(LiSiO_4)$	10.03	12.07	12.20	119.08	90.07	90.10	1291.13
65942	$Cs_2(NH_4)_4(Fe_2O_5)$	7.19	7.25	7.56	87.08	92.78	119.23	343.63
65976	$(NH_4)_{11}(Li(MnO_4)_4)$	8.85	8.82	10.69	115.36	113.80	89.66	675.73
65978	$(NH_4)_3Na_2(LiTeO_6)$	6.88	7.93	8.10	107.73	92.26	92.06	420.05
68706	$NH_4SnO(PO_4)$	6.71	10.92	13.42	90.31	89.65	90.21	983.56
69429	$NH_4(SbO)(SiO$	6.65	10.83	13.27	89.86	89.90	90.13	954.77

	$_4$)							
69556	$NH_4VO(PO_4)$	6.55	10.66	13.05	90.00	90.00	90.00	911.24
71536	$(NH_4)_2Co(SeO_3)_2$	5.46	5.60	7.47	67.82	68.58	60.80	179.91
71540	$(NH_4)_2Mn(SeO_3)_2$	5.68	5.68	7.58	67.99	68.05	59.99	191.25
72873	$(NH_4)_3(NbP_2O_9)$	5.20	9.89	19.81	90.08	90.21	90.47	1019.23
73205	$(NH_4)_5Li(Ge_2O_7)$	6.72	10.75	16.78	88.97	91.39	105.48	1167.25
74591	$NH_4(TiO)(AsO_4)$	6.75	10.97	13.37	89.75	89.69	89.63	989.23
74955	$Cs(NH_4)_4(TlO_4)$	7.65	13.53	22.93	90.00	90.00	91.15	2372.76
74956	$(NH_4)_4Na_2(Tl_2O_6)$	6.96	7.00	12.63	91.40	90.92	90.12	615.03
76891	$(NH_4)_2Pb(SO_4)_2$	5.64	5.90	8.81	69.79	63.63	57.05	218.35
79650	$NH_4(SnO)(PO_4)$	6.71	10.92	13.43	90.00	90.00	89.87	983.87
79651	$NH_4VO(PO_4)$	6.55	10.64	13.04	89.70	89.64	90.11	909.35
79702	$(NH_4)_2Ni(WO_2(PO_4)_2)$	9.49	9.43	10.77	90.14	89.77	90.07	964.34
80023	$NH_4(TiO)((P_{0.56}As_{0.44})O_4)$	13.20	6.71	10.85	90.14	89.78	89.61	960.44
80893	$NH_4GaF(PO_4)$	6.47	10.60	13.06	89.86	89.39	89.86	895.06
81479	$(NH_4)_6(VO)_4(SO_4)_8$	9.73	10.03	18.90	90.01	90.09	90.55	1844.83
82398	$(NH_4)_2(Be_2Si_3O_9)$	7.03	10.50	12.23	105.11	90.00	90.00	871.74
82457	$NH_4Li(Si_2O_5)$	4.82	6.30	8.45	94.93	89.99	90.03	255.75
83431	$(NH_4)_2Ca_2(CO_3)_3$	8.30	8.49	8.21	109.92	105.83	108.71	465.56
85404	$(NH_4)_6(CuSi_2O_8)$	6.77	6.77	8.08	92.16	87.74	70.67	348.41
85505	$(NH_4)_2Ca(NO_2)_4$	6.43	4.96	12.76	83.57	96.42	52.18	310.79
88843	$(NH_4)_2Zr(Ge_2O_7)$	5.70	5.78	13.74	77.30	78.04	60.45	381.73
91534	$NH_4(Ti_{0.936}Sn_{0.064})O(PO_4)$	13.02	6.56	10.69	89.91	89.56	89.83	913.43
95959	$(NH_4)_2(ZnP_2O_7)$	7.99	8.03	12.15	90.00	90.00	90.14	779.48
98708	$Na_{0.667}(NH_4)_{1.3}$	7.48	10.94	12.86	67.72	72.65	70.12	898.15

	$_{33}(Si_2O_5)$							
150841	$(NH_4)_2((Mo_{0.5}W_{0.5})O_4)$	11.89	6.98	8.05	80.34	118.38	94.03	579.29
170786	$(NH_4)_{1.33}Na_{0.67}Si_2O_5$	7.44	10.96	12.98	67.39	73.02	70.32	903.60
200310	$(NH_4)_2Ni(MoO_4)_2$	8.49	11.01	11.10	90.00	90.00	113.02	955.38
200388	$(NH_4)_8Zr(MoO_4)_6$	10.94	12.23	12.66	89.94	107.12	89.85	1618.43
200389	$(NH_4)_8Hf(MoO_4)_6$	10.89	12.16	12.75	90.00	107.86	90.01	1608.49
200613	$NH_4Fe(PO_4)F$	6.52	10.81	13.13	90.00	90.00	90.00	925.15
201788	$(NH_4)_2(SbPO_6)$	6.02	9.82	11.92	90.00	90.00	90.00	704.20
202071	$(NH_4)_3Li_3TeO_6$	6.13	9.88	12.47	90.00	88.76	90.00	754.32
202073	$(NH_4)_4Na_2(TeO_6)$	7.13	7.13	9.79	95.17	84.83	114.79	449.02
202428	$(NH_4)_3Bi_2(VO_4)_3$	7.35	10.28	10.12	88.36	73.24	72.87	698.80
202560	$(NH_4)_4Li(IO_6)$	10.17	11.91	14.17	90.00	89.93	90.00	1716.48
202980	$(NH_4)_3(NbAs_2O_9)$	5.48	8.36	23.43	90.00	89.63	90.00	1073.23
203214	$(NH_4)_2(Mn_3(OH)_2(VO_4)_2)$	5.54	6.37	8.21	112.86	101.09	90.00	261.09
203218	$NH_4(MoO_2)(AsO_4)$	6.72	7.29	11.07	90.00	90.00	90.00	542.13
280310	$(NH_4)_2(Al_2Sb_2O_7)$	5.68	5.75	9.01	89.95	82.24	120.43	250.79
280327	$(NH_4)_2(TiSi_3O_9)(H_2O)$	7.34	10.20	13.18	90.00	90.00	90.00	985.68
400850	$(NH_4)_2(TiO)_2(As_{.43}P_{.57}O_4)_2$	13.22	6.69	10.85	89.96	89.70	89.70	959.36
409484	$NH_4(Ag(CO_3))$	6.20	6.13	11.44	105.54	105.73	90.00	402.23
409582	$(NH_4)_3Bi_2(PO_4)_3$	7.08	10.13	9.78	88.09	72.68	71.73	634.82
411501	$(NH_4)_3(BSb_4O_{13})$	7.24	7.38	13.70	83.06	80.94	62.83	642.27
412877	$BaNH_4(FeO_3)$	6.07	6.92	13.03	90.00	90.00	115.96	491.82
417892	$(NH_4)_2Mn(V_2O_7)$	6.09	8.85	8.72	90.31	94.78	92.06	467.77

Table S6. Specific capacity C(mAh/g), type, open circuit voltage OCV(V), band gap(eV) and direct band gap ("T" represents a direct band gap, and "F" represents an indirect band gap) of the 72 compounds. ID is the ICSD Collection Code, STRU is the Struct formula. and Ver(%) is the volume expansion rate.

ID	STRU	C	type	OCV	band_gap	direct_gap	Ver
16557	NH_4TiNbO_5	121.22	layered	5.24	2.88	F	6.84
18022	$NH_4Zn(BeF_3)_3$	101.86	tunnel	6.85	6.14	F	8.19
30886	$NH_4Cu(PO_4)$	168.48	layered	4.90	0.23	T	14.17
30910	$NaNH_4GeO_3$	186.03	tunnel	4.37	3.31	F	7.19
31021	NH_4AgCO_3	159.46	layered	4.48	2.37	T	12.22
36380	$(NH_4)_2Na(FeO_3)$	421.87	tunnel	4.75	2.22	F	17.96
36612	$(NH_4)_2Na_4(FeO_3)_2$	178.59	tunnel	3.61	2.50	F	16.33
39440	NH_4CrPO_4F	161.38	tunnel	4.80	2.57	F	6.44
39697	$(NH_4)_2(Zr_{0\cdot 86}Ti_{0\cdot 14})(Si_3O_9)(H_2O)$	171.19	tunnel	3.76	0.04	F	9.35
39735	NH_4GeOPO_4	145.59	tunnel	5.04	3.15	T	7.45
48118	$NH_4Na_4(GaO_4)$	118.54	layered	3.83	2.40	T	10.84
48119	$Cs(NH_4)_4(GaO_4)$	401.33	layered	3.39	2.62	T	36.22
48177	$NH_4Li_2(BO_3)$	366.97	tunnel	3.39	4.34	T	7.76
59281	$(NH_4)_2MgWO_2(PO_4)_2$	124.60	tunnel	5.06	3.55	F	7.79
59285	$NH_4(TiO)(PO_4)$	168.48	tunnel	5.16	3.07	F	6.17
62064	$Cs(NH_4)_2AuO_2$	148.01	layered	3.33	3.24	F	29.85
62102	$(NH_4)_9Li_3(IO_6)_2O$	623.00	tunnel	4.46	2.33	F	18.95
62136	$(NH_4)_2Na_4(GaO_3)_2$	163.35	tunnel	4.02	2.60	F	14.03
63544	$NH_4Ni(AsO_4)$	135.30	layered	5.76	2.82	F	10.59
65260	$NH_4Li_4(AlO_4)$	273.36	tunnel	3.95	4.16	T	8.91
65291	$(NH_4)_3(LiSiO_4)$	811.79	layered	4.36	4.24	F	16.37
65942	$Cs_2(NH_4)_4(Fe_2O_5)$	329.71	layered	3.24	2.24	F	22.02
65976	$(NH_4)_{11}(Li(MnO_4)_4)$	610.10	layered	4.01	1.97	F	24.56
65978	$(NH_4)_3Na_2(LiTeO_6)$	290.13	layered	3.90	2.64	F	12.55
68706	$NH_4SnO(PO_4)$	116.47	tunnel	5.29	3.04	T	10.48
69429	$NH_4(SbO)(SiO_4)$	116.47	tunnel	5.33	2.67	F	6.83
69556	$NH_4VO(PO_4)$	165.36	tunnel	3.99	1.58	T	6.67
71536	$(NH_4)_2Co(SeO_3)_2$	171.18	layered	3.97	2.32	F	10.60
71540	$(NH_4)_2Mn(SeO_3)_2$	173.39	layered	3.88	3.62	F	12.57
72873	$(NH_4)_3(NbP_2O_9)$	268.79	tunnel	5.08	3.13	F	13.30
73205	$(NH_4)_5Li(Ge_2O_7)$	505.45	layered	3.87	3.58	F	17.93
74591	$NH_4(TiO)(AsO_4)$	131.97	tunnel	5.13	3.13	T	6.54
74955	$Cs(NH_4)_4(TlO_4)$	267.22	layered	3.20	1.72	T	35.42
74956	$(NH_4)_4Na_2(Tl_2O_6)$	194.83	tunnel	3.03	1.46	T	17.33

76891	$(NH_4)_2Pb(SO_4)_2$	134.28	layered	5.11	4.63	F	17.10
79650	$NH_4(SnO)(PO_4)$	116.47	tunnel	5.30	3.02	T	6.33
79651	$NH_4VO(PO_4)$	165.36	tunnel	3.98	1.55	T	5.22
79702	$(NH_4)_2Ni(WO_2(PO_4)_2)$	123.45	tunnel	4.93	3.38	F	7.61
80023	$NH_4(TiO)((P_{0.56}As_{0.44})O_4)$	150.20	tunnel	5.15	3.12	F	7.41
80893	$NH_4GaF(PO_4)$	145.59	tunnel	5.18	3.98	F	7.07
81479	$(NH_4)_6(VO)_4(SO_4)_8$	155.15	layered	5.24	1.30	F	13.19
82398	$(NH_4)_2(Be_2Si_3O_9)$	217.80	tunnel	4.92	5.03	F	5.03
82457	$NH_4Li(Si_2O_5)$	187.34	tunnel	4.99	4.98	F	9.40
83431	$(NH_4)_2Ca_2(CO_3)_3$	206.07	tunnel	4.35	4.52	T	10.60
85404	$(NH_4)_6(CuSi_2O_8)$	648.12	layered	3.65	0.44	T	20.04
85505	$(NH_4)_2Ca(NO_2)_4$	239.19	layered	3.89	2.11	T	10.64
88843	$(NH_4)_2Zr(Ge_2O_7)$	153.52	tunnel	4.86	4.00	F	10.28
91534	$NH_4(Ti_{0.936}Sn_{0.064})O(PO_4)$	163.80	tunnel	4.90			4.93
95959	$(NH_4)_2(ZnP_2O_7)$	224.18	tunnel	5.14	4.01	T	11.50
98708	$Na_{0.667}(NH_4)_{1.333}(Si_2O_5)$	235.96	tunnel	4.97	4.52	F	10.08
150841	$(NH_4)_2((Mo_{0.5}W_{0.5})O_4)$	262.64	layered	4.00	3.35	F	12.64
170786	$(NH_4)_{1.33}Na_{0.67}Si_2O_5$	235.32	tunnel	4.48	4.42	F	13.67
200310	$(NH_4)_2Ni(MoO_4)_2$	141.37	layered	5.16	3.85	F	9.34
200388	$(NH_4)_8Zr(MoO_4)_6$	203.91	tunnel	5.27	4.08	F	16.69
200389	$(NH_4)_8Hf(MoO_4)_6$	188.32	tunnel	5.07	4.25	F	16.99
200613	$NH_4Fe(PO_4)F$	157.58	tunnel	5.17	2.33	F	5.78
201788	$(NH_4)_2(SbPO_6)$	215.17	tunnel	5.29	2.73	F	14.94
202071	$(NH_4)_3Li_3TeO_6$	328.03	layered	3.84	2.78	T	11.19
202073	$(NH_4)_4Na_2(TeO_6)$	396.87	layered	3.98	2.80	F	12.27
202428	$(NH_4)_3Bi_2(VO_4)_3$	105.33	tunnel	4.57	2.68	T	10.48
202560	$(NH_4)_4Li(IO_6)$	465.90	layered	4.43	2.41	F	17.12
202980	$(NH_4)_3(NbAs_2O_9)$	207.67	layered	5.21	3.28	F	12.05
203214	$(NH_4)_2(Mn_3(OH)_2(VO_4)_2)$	124.89	tunnel	3.48	1.96	F	7.11
203218	$NH_4(MoO_2)(AsO_4)$	100.33	tunnel	5.24	2.86	F	9.93
280310	$(NH_4)_2(Al_2Sb_2O_7)$	130.68	layered	4.80	3.63	F	13.46
280327	$(NH_4)_2(TiSi_3O_9)(H_2O)$	194.12	tunnel	5.19	3.46	F	7.06
400850	$(NH_4)_2(TiO)_2(As_{.43}P_{.57}O_4)_2$	150.57	tunnel	5.15	3.16	T	7.11

409484	$NH_4(Ag(CO_3))$	159.46	layered	4.50	2.37	T	14.94
409582	$(NH_4)_3Bi_2(PO_4)_3$	114.32	tunnel	4.91	3.57	T	10.32
411501	$(NH_4)_3(BSb_4O_{13})$	113.67	tunnel	5.22	1.78	F	7.28
412877	$BaNH_4(FeO_3)$	111.16	tunnel	3.57	2.45	F	15.07
417892	$(NH_4)_2Mn(V_2O_7)$	199.17	layered	4.46	2.47	F	13.97

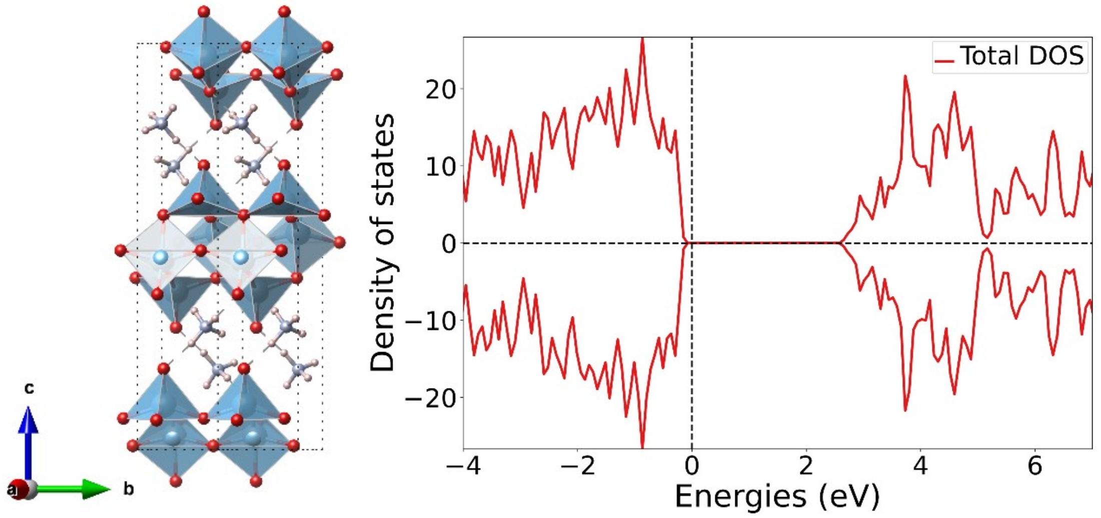

Figure S1. Structure diagram and density of states of NH_4TiNbO_5.

Table S7. Cell parameters of NH_4TiNbO_5, ICSD is the ICSD Collection Code, STRU is the chemical formula. The a, b, c (Å) and α, β, γ (°) are unit cell parameters. V (Å^3) is the cell volume and Structure parameters of NH_4TiNbO_5.

ICSD	STRU	a	b	c	α	β	γ	V
16557	NH_4TiNbO_5	6.61	3.83	19.09	90.00	90.00	90.02	483.36

NO.	E	x	y	z	NO.	E	x	y	z
1	N1	0.30208	0.24233	0.69764	29	O9	0.96053	0.23745	0.38226
2	N2	0.80208	0.24233	0.80236	30	O10	0.46053	0.23745	0.11774
3	N3	0.69824	0.75816	0.30237	31	O11	0.03969	0.76299	0.61776
4	N4	0.19824	0.75816	0.19763	32	O12	0.53969	0.76299	0.88224
5	Ti1	0.80623	0.24853	0.46647	33	O13	0.32882	0.27457	0.30079
6	Ti2	0.30623	0.24853	0.03353	34	O14	0.82882	0.27457	0.19921
7	Ti3	0.19399	0.75190	0.53354	35	O15	0.67138	0.72582	0.69922
8	Ti4	0.69399	0.75190	0.96646	36	O16	0.17138	0.72582	0.80078
9	Ti5	0.26111	0.25663	0.39052	37	O17	0.74260	0.24695	0.57731
10	Ti6	0.76111	0.25663	0.10948	38	O18	0.24260	0.24695	0.92269
11	Ti7	0.73912	0.74382	0.60950	39	O19	0.25762	0.75345	0.42271
12	Ti8	0.23912	0.74382	0.89050	40	O20	0.75762	0.75345	0.07729
13	Nb1	0.80623	0.24853	0.46647	41	H1	0.18661	0.08956	0.67668
14	Nb2	0.30623	0.24853	0.03353	42	H2	0.41517	0.07617	0.71441
15	Nb3	0.19399	0.75190	0.53354	43	H3	0.24983	0.40402	0.73850
16	Nb4	0.69399	0.75190	0.96646	44	H4	0.35986	0.40041	0.65870
17	Nb5	0.26111	0.25663	0.39052	45	H5	0.68661	0.08956	0.82332
18	Nb6	0.76111	0.25663	0.10948	46	H6	0.91517	0.07617	0.78559
19	Nb7	0.73912	0.74382	0.60950	47	H7	0.85986	0.40041	0.84130
20	Nb8	0.23912	0.74382	0.89050	48	H8	0.74983	0.40402	0.76150
21	O1	0.55729	0.25651	0.42713	49	H9	0.64046	0.60002	0.34130
22	O2	0.05729	0.25651	0.07287	50	H10	0.75048	0.59650	0.26150
23	O3	0.44293	0.74383	0.57288	51	H11	0.81370	0.91090	0.32334

24	O4	0.94293	0.74383	0.92712	52	H12	0.58516	0.92431	0.28560
25	O5	0.14580	0.25270	0.50988	53	H13	0.14046	0.60002	0.15870
26	O6	0.64580	0.25270	0.99012	54	H14	0.25048	0.59650	0.23850
27	O7	0.85441	0.74776	0.49015	55	H15	0.08516	0.92431	0.21440
28	O8	0.35441	0.74776	0.00985	56	H16	0.31370	0.91090	0.17666

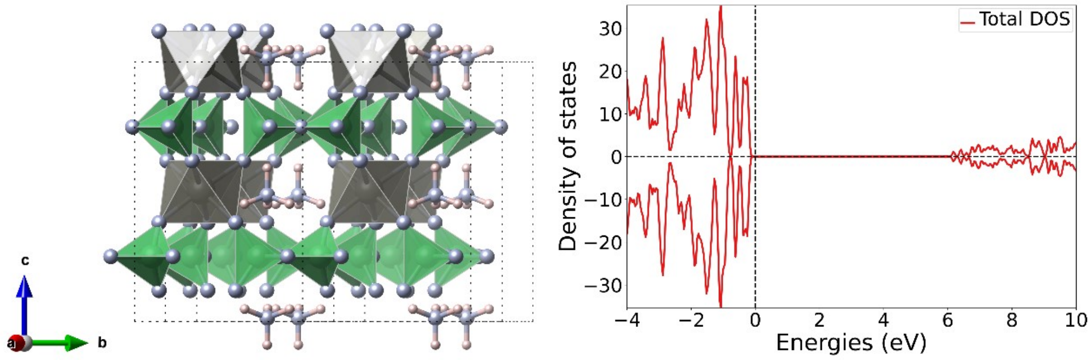

Figure S2. Structure diagram and density of states of $NH_4Zn(BeF_3)_3$.

Table S8. Cell parameters of $NH_4Zn(BeF_3)_3$, ICSD is the ICSD Collection Code, STRU is the chemical formula. The a, b, c (Å) and α, β, γ (°) are unit cell parameters. V (Å^3) is the cell volume and Structure parameters of $NH_4Zn(BeF_3)_3$.

ICSD	STRU	a	b	c	α	β	γ	V
18022	$NH_4Zn(BeF_3)_3$	6.78	6.78	9.80	90.00	90.00	120.01	390.72

NO.	E	x	y	z	NO.	E	x	y	z
1	Be1	0.79019	0.06537	0.25000	19	F1	0.76030	0.81577	0.25000
2	Be2	0.27505	0.20985	0.25000	20	F2	0.05531	0.23972	0.25000
3	Be3	0.93460	0.72508	0.25000	21	F3	0.18423	0.94478	0.25000
4	Be4	0.27450	0.06312	0.75000	22	F4	0.05418	0.81451	0.75000
5	Be5	0.93693	0.21132	0.75000	23	F5	0.18555	0.23968	0.75000
6	Be6	0.78860	0.72553	0.75000	24	F6	0.76041	0.94583	0.75000
7	Zn1	0.66670	0.33337	0.99320	25	F7	0.67210	0.08632	0.11910
8	Zn2	0.66670	0.33337	0.50680	26	F8	0.41419	0.32798	0.11912
9	H1	0.46263	0.82302	0.04696	27	F9	0.91374	0.58591	0.11918
10	H2	0.36053	0.53745	0.04736	28	F10	0.67210	0.08632	0.38090
11	H3	0.33109	0.66570	0.90935	29	F11	0.41419	0.32798	0.38088
12	H4	0.17701	0.63952	0.04844	30	F12	0.91374	0.58591	0.38082
13	H5	0.46263	0.82302	0.45304	31	F13	0.41222	0.08440	0.61900
14	H6	0.36053	0.53745	0.45264	32	F14	0.91567	0.32782	0.61901
15	H7	0.17701	0.63952	0.45156	33	F15	0.67222	0.58781	0.61898
16	H8	0.33109	0.66570	0.59065	34	F16	0.41222	0.08440	0.88100
17	N1	0.33291	0.66646	0.01351	35	F17	0.91567	0.32782	0.88099
18	N2	0.33291	0.66646	0.48649	36	F18	0.67222	0.58781	0.88102

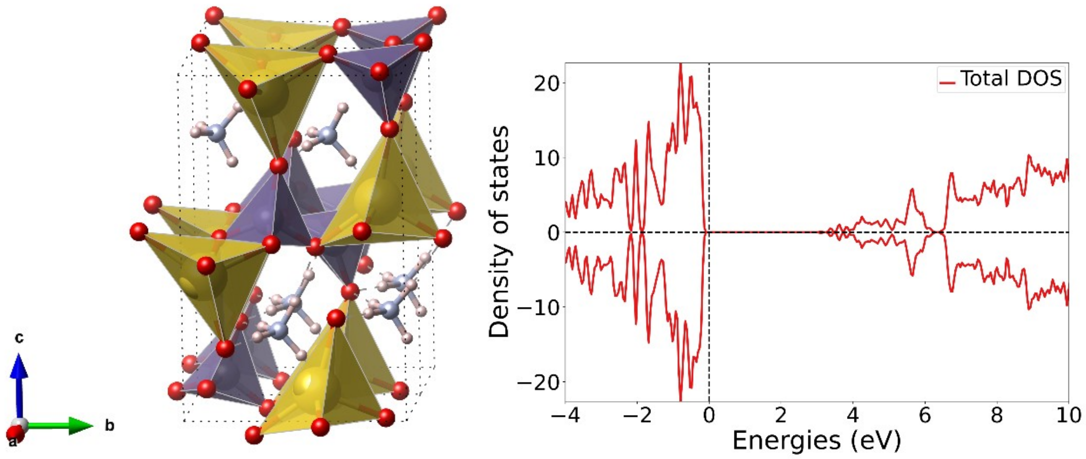

Figure S3. Structure diagram and density of states of $NaNH_4GeO_3$.

Table S9. Cell parameters of $NaNH_4GeO_3$, ICSD is the ICSD Collection Code, STRU is the chemical formula. The a, b, c (Å) and α, β, γ (°) are unit cell parameters. V (Å^3) is the cell volume and Structure parameters of $NaNH_4GeO_3$.

ICSD	STRU	a	b	c	α	β	γ	V
30910	$NaNH_4GeO_3$	4.88	7.04	11.04	90.00	90.00	89.93	378.93

NO.	E	x	y	z	NO.	E	x	y	z
1	Na1	0.46265	0.35562	0.90607	21	H13	0.99727	0.67425	0.82726
2	Na2	0.96408	0.59725	0.08467	22	H14	0.96584	0.70528	0.67164
3	Na3	0.96405	0.09726	0.41515	23	H15	0.79967	0.52459	0.74149
4	Na4	0.46263	0.85559	0.59373	24	H16	0.15971	0.52251	0.73019
5	Ge1	0.50560	0.36454	0.54310	25	N1	0.98012	0.10530	0.75376
6	Ge2	0.00171	0.62253	0.44316	26	N2	0.46701	0.90064	0.27907
7	Ge3	0.00173	0.12256	0.05666	27	N3	0.46707	0.40063	0.22074
8	Ge4	0.50564	0.86455	0.95670	28	N4	0.98006	0.60532	0.74606
9	H1	0.96589	0.20527	0.82817	29	O1	0.86685	0.39887	0.50029
10	H2	0.99730	0.17423	0.67256	30	O2	0.36895	0.56485	0.45479
11	H3	0.79974	0.02456	0.75834	31	O3	0.36897	0.06488	0.04499
12	H4	0.15978	0.02250	0.76963	32	O4	0.86688	0.89890	0.99955
13	H5	0.46250	0.99874	0.35121	33	O5	0.46532	0.41335	0.69696
14	H6	0.46180	0.97475	0.19753	34	O6	0.94863	0.68931	0.29291
15	H7	0.28894	0.81661	0.28194	35	O7	0.94876	0.18937	0.20692
16	H8	0.64930	0.81583	0.28418	36	O8	0.46539	0.91332	0.80283
17	H9	0.46184	0.47474	0.30228	37	O9	0.42957	0.14106	0.48355
18	H10	0.46256	0.49873	0.14860	38	O10	0.94313	0.81117	0.54429
19	H11	0.28900	0.31659	0.21786	39	O11	0.94312	0.31119	0.95552
20	H12	0.64939	0.31585	0.21564	40	O12	0.42954	0.64107	0.01626

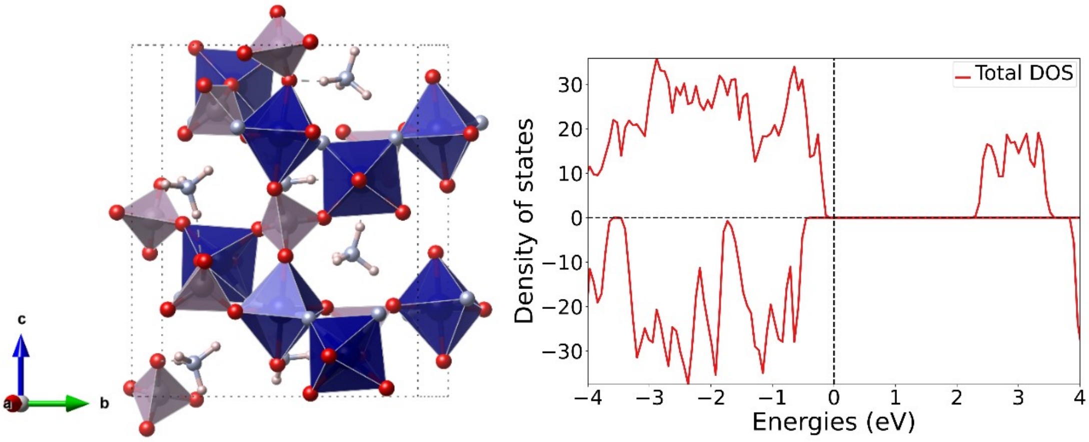

Figure S4. Structure diagram and density of states of NH_4CrPO_4F.

Table S10. Cell parameters of NH_4CrPO_4F, ICSD is the ICSD Collection Code, STRU is the chemical formula. The a, b, c (Å) and α, β, γ (°) are unit cell parameters. V (Å^3) is the cell volume and Structure parameters of NH_4CrPO_4F.

ICSD	STRU	a	b	c	α	β	γ	V
39440	NH_4CrPO_4F	6.49	10.76	13.04	90.00	90.00	90.00	910.87

NO.	E	x	y	z	NO.	E	x	y	z
1	Cr1	0.25006	0.99612	0.75326	49	N1	0.21788	0.45025	0.60856
2	Cr2	0.75024	0.49612	0.24673	50	N2	0.78234	0.95025	0.39144
3	Cr3	0.75006	0.49612	0.74674	51	N3	0.71788	0.95025	0.89144
4	Cr4	0.25024	0.99612	0.25327	52	N4	0.28234	0.45025	0.10856
5	Cr5	0.01005	0.25040	0.88300	53	N5	0.84354	0.18032	0.59487
6	Cr6	0.99027	0.75040	0.11700	54	N6	0.15669	0.68033	0.40514
7	Cr7	0.51005	0.75040	0.61700	55	N7	0.34354	0.68032	0.90513
8	Cr8	0.49027	0.25040	0.38300	56	N8	0.65669	0.18033	0.09486
9	P1	0.50176	0.24658	0.81487	57	O1	0.46326	0.12950	0.74761
10	P2	0.49855	0.74659	0.18511	58	O2	0.53700	0.62950	0.25237
11	P3	0.00176	0.74658	0.68513	59	O3	0.96326	0.62950	0.75239
12	P4	0.99855	0.24659	0.31489	60	O4	0.03700	0.12950	0.24763
13	P5	0.33231	0.00400	0.50612	61	O5	0.54634	0.35692	0.74138
14	P6	0.66794	0.50400	0.49389	62	O6	0.45408	0.85696	0.25857
15	P7	0.83231	0.50400	0.99388	63	O7	0.04634	0.85692	0.75862
16	P8	0.16794	0.00400	0.00611	64	O8	0.95408	0.35696	0.24143
17	H1	0.06170	0.43630	0.59683	65	O9	0.69546	0.22143	0.88483
18	H2	0.30900	0.41991	0.54720	66	O10	0.30482	0.72145	0.11516
19	H3	0.27301	0.40760	0.67363	67	O11	0.19546	0.72143	0.61517
20	H4	0.23543	0.54528	0.61722	68	O12	0.80482	0.22145	0.38484
21	H5	0.72719	0.90762	0.32636	69	O13	0.31531	0.27537	0.88461
22	H6	0.93853	0.93630	0.40316	70	O14	0.68501	0.77533	0.11535
23	H7	0.69123	0.91992	0.45279	71	O15	0.81531	0.77537	0.61539
24	H8	0.76481	0.04529	0.38279	72	O16	0.18501	0.27533	0.38465

25	H9	0.56170	0.93630	0.90317	73	O17	0.20133	0.97813	0.40841
26	H10	0.80900	0.91991	0.95280	74	O18	0.79894	0.47814	0.59158
27	H11	0.73543	0.04528	0.88278	75	O19	0.70133	0.47813	0.09159
28	H12	0.77301	0.90760	0.82637	76	O20	0.29894	0.97814	0.90842
29	H13	0.22719	0.40762	0.17364	77	O21	0.18892	0.01741	0.60080
30	H14	0.43853	0.43630	0.09684	78	O22	0.81132	0.51740	0.39920
31	H15	0.26481	0.54529	0.11721	79	O23	0.68892	0.51741	0.89920
32	H16	0.19123	0.41992	0.04721	80	O24	0.31132	0.01740	0.10080
33	H17	0.70124	0.14415	0.61355	81	O25	0.48225	0.89287	0.52374
34	H18	0.96092	0.11484	0.60316	82	O26	0.51797	0.39288	0.47626
35	H19	0.87113	0.25418	0.64267	83	O27	0.98225	0.39287	0.97626
36	H20	0.83823	0.20900	0.51823	84	O28	0.01797	0.89288	0.02374
37	H21	0.03928	0.61486	0.39685	85	O29	0.46714	0.12151	0.49131
38	H22	0.29897	0.64415	0.38645	86	O30	0.53312	0.62152	0.50868
39	H23	0.16201	0.70900	0.48178	87	O31	0.96714	0.62151	0.00869
40	H24	0.12910	0.75420	0.35735	88	O32	0.03312	0.12152	0.99132
41	H25	0.20124	0.64415	0.88645	89	F1	0.46739	0.86727	0.73443
42	H26	0.46092	0.61484	0.89684	90	F2	0.53293	0.36725	0.26555
43	H27	0.33823	0.70900	0.98177	91	F3	0.96739	0.36727	0.76557
44	H28	0.37113	0.75418	0.85733	92	F4	0.03293	0.86725	0.23445
45	H29	0.53928	0.11486	0.10315	93	F5	0.02944	0.11679	0.77858
46	H30	0.79897	0.14415	0.11355	94	F6	0.97084	0.61681	0.22144
47	H31	0.62910	0.25420	0.14265	95	F7	0.52944	0.61679	0.72142
48	H32	0.66201	0.20900	0.01822	96	F8	0.47084	0.11681	0.27856

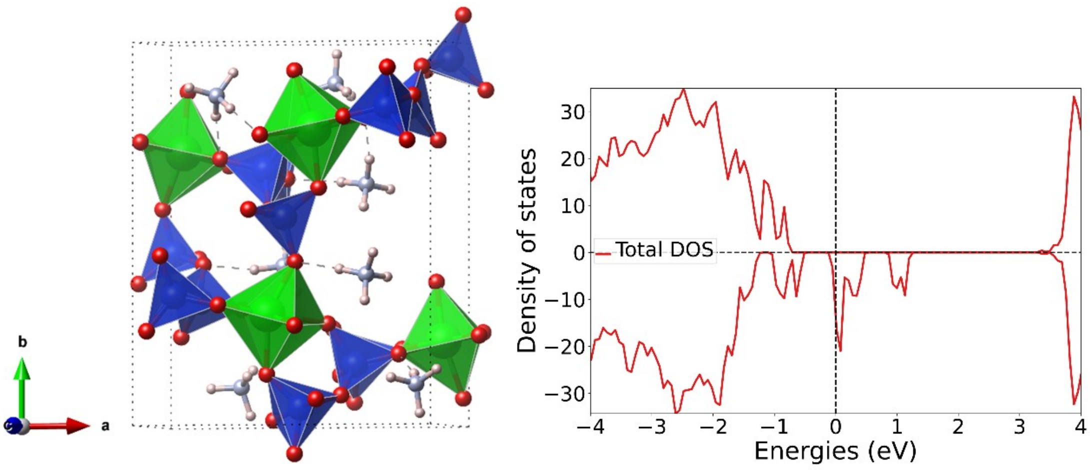

Figure S5. Structure diagram and density of states of $(NH_4)_2(Zr_{0.86}Ti_{0.14})(Si_3O_9)$.

Table S11. Cell parameters of $(NH_4)_2(Zr_{0.86}Ti_{0.14})(Si_3O_9)$, ICSD is the ICSD Collection Code, STRU is the chemical formula. The a, b, c (Å) and α, β, γ (°) are unit cell parameters. V ($Å^3$) is the cell volume and Structure parameters of $(NH_4)_2(Zr_{0.86}Ti_{0.14})(Si_3O_9)$.

ICSD	STRU	a	b	c	α	β	γ	V
39697	$(NH_4)_2(Zr_{0.86}Ti_{0.14})(Si_3O_9)(H_2O)$	10.64	13.47	7.40	89.10	90.62	89.63	1060.22

NO.	E	x	y	z	NO.	E	x	y	z
1	N1	0.92187	0.10656	0.77000	51	O23	0.41990	0.13388	0.76343
2	N2	0.58267	0.89650	0.24043	52	O24	0.08656	0.87664	0.23232
3	N3	0.40411	0.42107	0.20802	53	O25	0.91097	0.86044	0.76225
4	N4	0.04119	0.61057	0.76556	54	O26	0.60201	0.13488	0.26574
5	N5	0.76654	0.39194	0.86370	55	O27	0.41759	0.64843	0.22178
6	N6	0.69661	0.63234	0.31662	56	O28	0.09192	0.36274	0.69318
7	N7	0.26301	0.10476	0.21000	57	O29	0.86618	0.93098	0.08277
8	N8	0.24157	0.86200	0.68792	58	O30	0.64867	0.07952	0.60756
9	Zr1	0.94341	0.22316	0.22761	59	O31	0.37770	0.56332	0.91149
10	Zr2	0.57268	0.77350	0.73629	60	O32	0.15496	0.42354	0.36051
11	Zr3	0.42219	0.29102	0.76664	61	O33	0.89837	0.93829	0.43785
12	Zr4	0.07419	0.71686	0.25317	62	O34	0.60801	0.06687	0.94256
13	Ti1	0.94341	0.22316	0.22761	63	O35	0.39845	0.56510	0.54445
14	Ti2	0.57268	0.77350	0.73629	64	O36	0.09454	0.44251	0.01248
15	Ti3	0.42219	0.29102	0.76664	65	O37	0.85319	0.58720	0.03909
16	Ti4	0.07419	0.71686	0.25317	66	O38	0.61809	0.34324	0.41870
17	Si1	0.84867	0.83101	0.96057	67	O39	0.28673	0.89831	0.20320
18	Si2	0.67572	0.16020	0.45227	68	O40	0.41669	0.01513	0.56005
19	Si3	0.34927	0.66880	0.02029	69	H1	0.84964	0.12306	0.85888
20	Si4	0.16118	0.32684	0.50392	70	H2	0.93429	0.03091	0.76603
21	Si5	0.86999	0.83378	0.55101	71	H3	-0.00165	0.14399	0.82459

22	Si6	0.63503	0.16894	0.05277	72	H4	0.89853	0.13437	0.64095
23	Si7	0.37070	0.67102	0.43178	73	H5	0.49148	0.87380	0.25931
24	Si8	0.12733	0.33804	0.90822	74	H6	0.63521	0.87281	0.35372
25	Si9	0.96972	0.95843	0.24263	75	H7	0.58567	0.97258	0.22552
26	Si10	0.53050	0.04663	0.74287	76	H8	0.61944	0.86456	0.12333
27	Si11	0.47475	0.54422	0.73872	77	H9	0.31273	0.41135	0.25354
28	Si12	0.03692	0.45411	0.21863	78	H10	0.44193	0.35540	0.16201
29	O1	0.82031	0.18785	0.44019	79	H11	0.45529	0.44672	0.31579
30	O2	0.70084	0.80471	0.94217	80	H12	0.40137	0.47255	0.10129
31	O3	0.30953	0.30395	0.54386	81	H13	0.96503	0.59753	0.85100
32	O4	0.19916	0.68490	0.04317	82	H14	0.07327	0.54441	0.71630
33	O5	0.00868	0.07283	0.21594	83	H15	0.11024	0.64050	0.85087
34	O6	0.51376	0.92596	0.76705	84	H16	0.01597	0.65946	0.65959
35	O7	0.51418	0.42744	0.75459	85	H17	0.67249	0.40705	0.82315
36	O8	0.00490	0.57018	0.25734	86	H18	0.77184	0.32409	0.93785
37	O9	0.78174	0.20416	0.04553	87	H19	0.80013	0.44861	0.94305
38	O10	0.72089	0.80453	0.54164	88	H20	0.82275	0.38644	0.75253
39	O11	0.27758	0.31396	0.92923	89	H21	0.60626	0.63897	0.26412
40	O12	0.22210	0.69931	0.43532	90	H22	0.69933	0.57356	0.40609
41	O13	0.92960	0.74086	0.05749	91	H23	0.71654	0.69624	0.39096
42	O14	0.59464	0.26690	0.53460	92	H24	0.76238	0.61973	0.21038
43	O15	0.41619	0.75812	0.90956	93	H25	0.16353	0.10159	0.21799
44	O16	0.08773	0.23538	0.41363	94	H26	0.29197	0.03070	0.19370
45	O17	0.95897	0.74461	0.47836	95	H27	0.29837	0.13214	0.32862
46	O18	0.54192	0.25893	0.98632	96	H28	0.28837	0.14992	0.10351
47	O19	0.45793	0.75849	0.51164	97	H29	0.15719	0.88265	0.74159
48	O20	0.04575	0.24632	0.98765	98	H30	0.22623	0.80999	0.58640
49	O21	0.92200	0.37659	0.24106	99	H31	0.30013	0.82797	0.78769
50	O22	0.59230	0.62061	0.74938	100	H32	0.29078	0.92251	0.63488

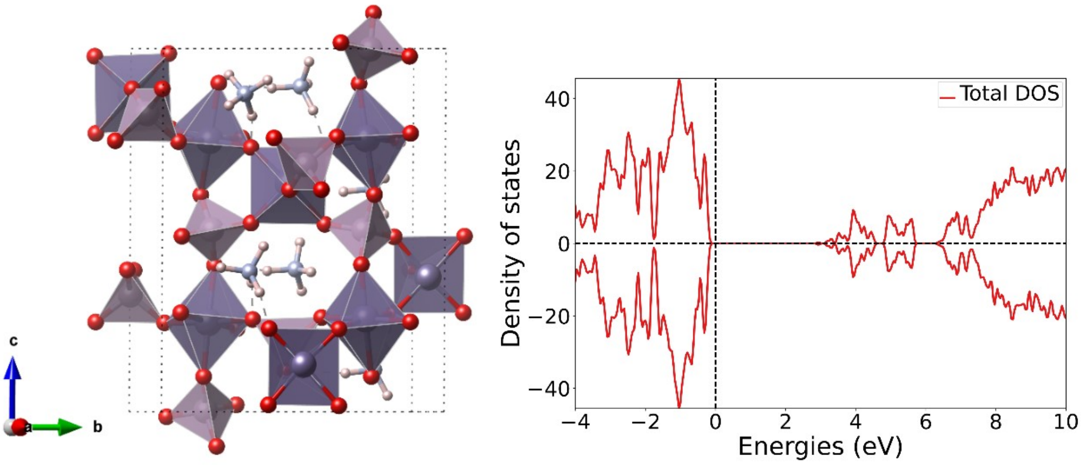

Figure S6. Structure diagram and density of states of NH_4GeOPO_4.

Table S12. Cell parameters of NH_4GeOPO_4, ICSD is the ICSD Collection Code, STRU is the chemical formula. The a, b, c (Å) and α, β, γ (°) are unit cell parameters. V (Å^3) is the cell volume and Structure parameters of NH_4GeOPO_4.

ICSD	STRU	a	b	c	α	β	γ	V
39735	NH_4GeOPO_4	6.55	10.15	12.84	89.70	89.80	89.89	853.99

NO.	E	x	y	z	NO.	E	x	y	z
1	Ge1	0.00338	0.00157	0.87098	49	N1	0.71965	0.32167	0.87550
2	Ge2	0.98982	0.50051	0.13028	50	N2	0.28764	0.83275	0.11558
3	Ge3	0.50533	0.50112	0.62882	51	N3	0.20949	0.83211	0.61413
4	Ge4	0.48815	0.99846	0.37100	52	N4	0.78845	0.33319	0.38579
5	Ge5	0.24382	0.25181	0.74802	53	N5	0.82274	0.07399	0.60526
6	Ge6	0.75327	0.74861	0.25656	54	N6	0.18623	0.56449	0.39278
7	Ge7	0.74218	0.75050	0.75245	55	N7	0.32283	0.56416	0.89613
8	Ge8	0.25296	0.24933	0.24613	56	N8	0.68474	0.06306	0.10689
9	P1	0.16088	0.25125	0.00245	57	O1	0.01930	0.13043	0.98635
10	P2	0.83606	0.75104	0.99852	58	O2	0.98004	0.63030	0.01642
11	P3	0.66240	0.75067	0.50028	59	O3	0.51889	0.62964	0.51635
12	P4	0.33594	0.25027	0.50113	60	O4	0.47695	0.12888	0.48349
13	P5	0.99546	0.50541	0.68064	61	O5	0.02425	0.37602	0.01929
14	P6	0.00203	0.00201	0.32108	62	O6	0.97418	0.87401	0.97881
15	P7	0.49140	0.00164	0.82062	63	O7	0.52522	0.87445	0.48174
16	P8	0.50438	0.50313	0.18174	64	O8	0.47254	0.37343	0.51970
17	H1	0.62522	0.35785	0.81456	65	O9	0.29698	0.27654	0.90492
18	H2	0.62446	0.27569	0.92731	66	O10	0.70526	0.77613	0.09726
19	H3	0.80371	0.39173	0.91335	67	O11	0.79388	0.77348	0.59797
20	H4	0.82064	0.25419	0.84352	68	O12	0.20327	0.27605	0.40326
21	H5	0.30762	0.87865	0.04484	69	O13	0.30270	0.22834	0.09714
22	H6	0.14691	0.85748	0.14640	70	O14	0.69595	0.72751	0.90358
23	H7	0.39901	0.85727	0.17174	71	O15	0.80461	0.72729	0.40531
24	H8	0.29192	0.73169	0.10628	72	O16	0.19299	0.22696	0.59680

25	H9	0.18579	0.87658	0.54317	73	O17	0.45165	0.37461	0.72757
26	H10	0.09838	0.85641	0.67060	74	O18	0.54746	0.87362	0.27223
27	H11	0.34985	0.85921	0.64332	75	O19	0.94102	0.88310	0.76675
28	H12	0.20815	0.73086	0.60585	76	O20	0.04825	0.37474	0.22898
29	H13	0.89947	0.35720	0.32855	77	O21	0.53622	0.63022	0.72712
30	H14	0.64737	0.35746	0.35541	78	O22	0.45775	0.12832	0.27268
31	H15	0.80906	0.38019	0.45555	79	O23	0.03004	0.13883	0.77913
32	H16	0.79272	0.23250	0.39705	80	O24	0.96071	0.63075	0.22786
33	H17	0.80974	0.04102	0.52937	81	O25	0.18269	0.54130	0.61165
34	H18	0.68550	0.11381	0.62807	82	O26	0.81340	0.03581	0.38918
35	H19	0.93865	0.14264	0.60910	83	O27	0.67732	0.03369	0.88938
36	H20	0.85871	0.99854	0.65709	84	O28	0.31702	0.53960	0.11455
37	H21	0.08940	0.59816	0.33235	85	O29	0.80724	0.47127	0.61172
38	H22	0.13381	0.58546	0.46635	86	O30	0.18976	0.96871	0.39074
39	H23	0.33020	0.60307	0.38489	87	O31	0.30106	0.96977	0.88914
40	H24	0.19977	0.46328	0.38806	88	O32	0.69359	0.47222	0.11267
41	H25	0.40406	0.59888	0.82984	89	O33	0.94974	0.62105	0.75649
42	H26	0.17841	0.60306	0.90279	90	O34	0.04699	0.11796	0.24505
43	H27	0.39839	0.58423	0.96390	91	O35	0.44634	0.11785	0.74399
44	H28	0.30814	0.46309	0.89143	92	O36	0.54980	0.61650	0.25965
45	H29	0.63236	0.07982	0.03252	93	O37	0.04599	0.38824	0.75389
46	H30	0.58693	0.09738	0.16657	94	O38	0.95543	0.88475	0.24738
47	H31	0.82749	0.10389	0.11227	95	O39	0.53658	0.88435	0.74635
48	H32	0.70145	0.96224	0.11469	96	O40	0.45712	0.38337	0.25426

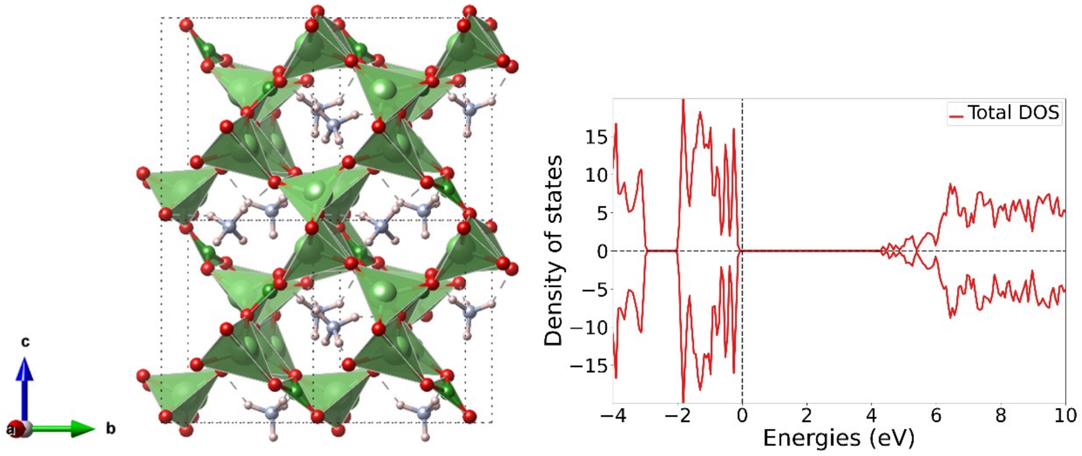

Figure S7. Structure diagram and density of states of $NH_4Li_2(BO_3)$.

Table S13. Cell parameters of $NH_4Li_2(BO_3)$, ICSD is the ICSD Collection Code, STRU is the chemical formula. The a, b, c (Å) and α, β, γ (°) are unit cell parameters. V ($Å^3$) is the cell volume and Structure parameters of $NH_4Li_2(BO_3)$.

ICSD	STRU	a	b	c	α	β	γ	V
48177	$NH_4Li_2(BO_3)$	6.44	6.50	8.52	90.00	90.12	90.00	356.73

NO.	E	x	y	z	NO.	E	x	y	z
1	Li1	0.52244	0.54924	0.35661	23	H11	0.38025	0.69281	0.10734
2	Li2	0.94312	0.96318	0.85587	24	H12	0.29784	0.45045	0.08605
3	Li3	0.02600	0.46290	0.62901	25	H13	0.29010	0.88715	0.42732
4	Li4	0.44408	0.06378	0.13156	26	H14	0.12113	0.84839	0.58224
5	Li5	0.44408	0.43622	0.63156	27	H15	0.29784	0.04955	0.58605
6	Li6	0.02600	0.03710	0.12901	28	H16	0.38025	0.80719	0.60734
7	Li7	0.94312	0.53682	0.35587	29	N1	0.76985	0.40831	0.96934
8	Li8	0.52244	0.95076	0.85661	30	N2	0.76985	0.09169	0.46934
9	B1	0.72757	0.83350	0.14961	31	N3	0.26958	0.59922	0.04599
10	B2	0.72757	0.66650	0.64961	32	N4	0.26958	0.90078	0.54599
11	B3	0.23646	0.17197	0.83810	33	O1	0.73105	0.98118	0.02935
12	B4	0.23646	0.32803	0.33810	34	O2	0.73105	0.51882	0.52935
13	H1	0.65975	0.47834	0.89957	35	O3	0.23109	0.03040	0.96253
14	H2	0.70957	0.27740	0.02089	36	O4	0.23109	0.46960	0.46253
15	H3	0.82231	0.51097	0.05385	37	O5	0.91016	0.73169	0.18787
16	H4	0.89920	0.34847	0.89313	38	O6	0.54221	0.69884	0.73029
17	H5	0.65975	0.02166	0.39957	39	O7	0.42013	0.20336	0.75895
18	H6	0.70957	0.22260	0.52089	40	O8	0.04922	0.24194	0.28704
19	H7	0.89920	0.15153	0.39313	41	O9	0.04922	0.25806	0.78704
20	H8	0.82231	0.98903	0.55385	42	O10	0.42013	0.29664	0.25895
21	H9	0.29010	0.61285	0.92732	43	O11	0.54221	0.80116	0.23029
22	H10	0.12113	0.65161	0.08224	44	O12	0.91016	0.76831	0.68787

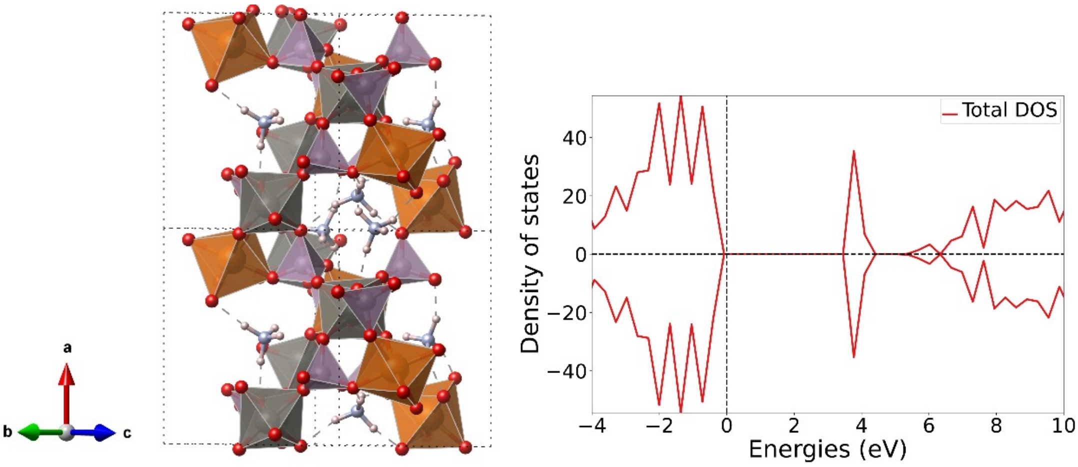

Figure S8. Structure diagram and density of states of $(NH_4)_2MgWO_2(PO_4)_2$.

Table S14. Cell parameters of $(NH_4)_2MgWO_2(PO_4)_2$, ICSD is the ICSD Collection Code, STRU is the chemical formula. The a, b, c (Å) and α, β, γ (°) are unit cell parameters. V ($Å^3$) is the cell volume and Structure parameters of $(NH_4)_2MgWO_2(PO_4)_2$.

ICSD	STRU	a	b	c	α	β	γ	V
59281	$(NH_4)_2MgWO_2(PO_4)_2$	9.43	9.45	10.87	89.66	90.53	89.80	968.91

NO.	E	x	y	z	NO.	E	x	y	z
1	Mg1	0.36009	0.62672	0.99342	49	N1	0.00900	0.70002	0.94590
2	Mg2	0.64796	0.35857	0.50083	50	N2	0.99395	0.34349	0.34235
3	Mg3	0.13405	0.12551	0.75897	51	N3	0.15917	0.50200	0.67301
4	Mg4	0.85732	0.84376	0.25294	52	N4	0.73319	0.47632	0.06836
5	P1	0.67240	0.81535	0.99411	53	N5	0.49198	0.18824	0.80449
6	P2	0.32203	0.17857	0.50524	54	N6	0.49520	0.78984	0.39379
7	P3	0.32884	0.81490	0.73569	55	N7	0.34024	0.00409	0.08343
8	P4	0.66514	0.15614	0.25470	56	N8	0.72167	0.00244	0.55937
9	P5	0.82484	0.31472	0.76503	57	O1	0.77305	0.23436	0.88404
10	P6	0.17210	0.68008	0.24518	58	O2	0.21289	0.77813	0.35679
11	P7	0.16834	0.31340	0.01560	59	O3	0.75497	0.71043	0.62707
12	P8	0.83595	0.66153	0.50761	60	O4	0.24386	0.28331	0.14240
13	H1	0.08649	0.75320	0.89763	61	O5	0.72491	0.73515	0.87571
14	H2	0.04834	0.59661	0.95683	62	O6	0.27959	0.27557	0.39186
15	H3	0.91127	0.70533	0.90116	63	O7	0.75190	0.19925	0.13720
16	H4	0.99909	0.74177	0.03266	64	O8	0.25639	0.78280	0.60883
17	H5	0.09333	0.30628	0.36967	65	O9	0.71396	0.97592	0.97166
18	H6	0.91696	0.26535	0.33907	66	O10	0.26713	0.02619	0.46515
19	H7	0.00475	0.38927	0.25748	67	O11	0.49141	0.77441	0.71236
20	H8	0.95233	0.41851	0.40350	68	O12	0.50725	0.21302	0.22749
21	H9	0.22866	0.43079	0.63405	69	O13	0.78297	0.47581	0.79086
22	H10	0.07910	0.43700	0.70964	70	O14	0.23240	0.52827	0.28606
23	H11	0.11710	0.57165	0.60880	71	O15	0.00801	0.26766	0.04023

24	H12	0.20812	0.56110	0.74130	72	O16	0.99617	0.71139	0.53032
25	H13	0.75624	0.46958	0.97615	73	O17	0.23058	0.21558	0.91684
26	H14	0.76801	0.38388	0.11101	74	O18	0.77399	0.73904	0.39682
27	H15	0.62305	0.48009	0.07838	75	O19	0.74131	0.26187	0.65362
28	H16	0.77161	0.57142	0.10321	76	O20	0.25165	0.72845	0.13065
29	H17	0.58905	0.18831	0.85015	77	O21	0.26908	0.71521	0.83466
30	H18	0.44719	0.08709	0.79528	78	O22	0.72751	0.23516	0.36502
31	H19	0.50582	0.22768	0.71689	79	O23	0.75461	0.75820	0.10572
32	H20	0.41655	0.24652	0.85064	80	O24	0.24224	0.22743	0.61903
33	H21	0.55056	0.87168	0.35038	81	O25	0.17772	0.46996	0.98173
34	H22	0.54083	0.69523	0.36921	82	O26	0.83243	0.50038	0.49759
35	H23	0.38617	0.79107	0.37248	83	O27	0.98467	0.30025	0.75300
36	H24	0.50501	0.79731	0.48755	84	O28	0.01200	0.67558	0.22874
37	H25	0.41899	0.93572	0.04861	85	O29	0.31461	0.97038	0.76971
38	H26	0.26678	0.93652	0.12142	86	O30	0.66450	0.99564	0.27083
39	H27	0.29459	0.06538	0.01385	87	O31	0.51220	0.80050	0.00414
40	H28	0.38264	0.07153	0.14809	88	O32	0.48295	0.17845	0.52280
41	H29	0.76135	0.08564	0.61216	89	O33	0.99521	0.03604	0.88605
42	H30	0.72379	0.90598	0.60534	90	O34	0.97791	0.95015	0.38727
43	H31	0.78135	0.99376	0.48088	91	O35	0.55740	0.49416	0.62591
44	H32	0.61984	0.03654	0.53585	92	O36	0.44801	0.48920	0.12392
45	W1	0.88402	0.09775	0.00734	93	O37	0.50356	0.54102	0.86763
46	W2	0.10872	0.89135	0.49832	94	O38	0.51418	0.45194	0.36765
47	W3	0.61708	0.60647	0.74823	95	O39	0.94728	0.98142	0.12682
48	W4	0.38718	0.38907	0.25622	96	O40	0.04850	0.99150	0.62715

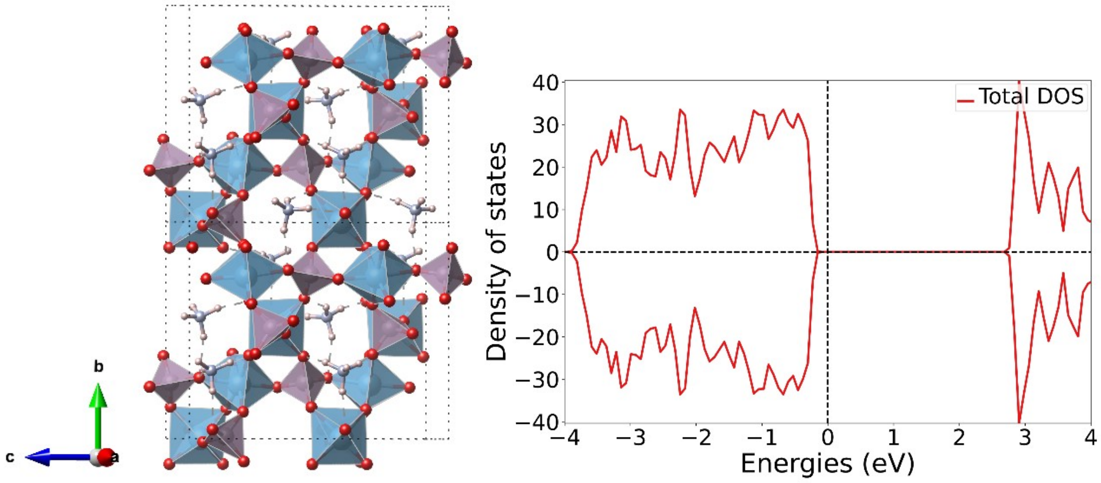

Figure S9. Structure diagram and density of states of $NH_4(TiO)(PO_4)$.

Table S15. Cell parameters of $NH_4(TiO)(PO_4)$, ICSD is the ICSD Collection Code, STRU is the chemical formula. The a, b, c (Å) and α, β, γ (°) are unit cell parameters. V ($Å^3$) is the cell volume and Structure parameters of $NH_4(TiO)(PO_4)$.

ICSD	STRU	a	b	c	α	β	γ	V
59285	$NH_4(TiO)(PO_4)$	6.58	10.71	13.05	89.85	90.06	89.97	919.61

NO.	E	x	y	z	NO.	E	x	y	z
1	Ti1	0.00210	0.00112	0.86879	49	N1	0.70356	0.31413	0.88884
2	Ti2	0.00436	0.50401	0.12939	50	N2	0.28600	0.82535	0.12151
3	Ti3	0.49692	0.50364	0.62921	51	N3	0.21457	0.82539	0.62087
4	Ti4	0.49911	0.00144	0.36899	52	N4	0.79125	0.31435	0.38780
5	Ti5	0.23146	0.25172	0.73924	53	N5	0.81171	0.05787	0.60083
6	Ti6	0.76652	0.74921	0.25798	54	N6	0.18947	0.56301	0.39727
7	Ti7	0.73441	0.74896	0.75791	55	N7	0.31203	0.56274	0.89705
8	Ti8	0.26861	0.25153	0.23960	56	N8	0.68830	0.05852	0.10016
9	P1	0.15682	0.25936	0.99617	57	O1	0.94671	0.88372	0.77649
10	P2	0.84490	0.75960	0.00210	58	O2	0.06209	0.38570	0.21969
11	P3	0.65665	0.75956	0.50209	59	O3	0.44049	0.38527	0.71953
12	P4	0.34268	0.25941	0.49593	60	O4	0.55471	0.88397	0.27663
13	P5	0.98992	0.51052	0.68076	61	O5	0.03806	0.13518	0.77243
14	P6	0.00760	0.01109	0.31620	62	O6	0.96637	0.63700	0.22593
15	P7	0.49363	0.01098	0.81592	63	O7	0.53499	0.63682	0.72581
16	P8	0.51201	0.51053	0.18079	64	O8	0.46322	0.13531	0.27258
17	H1	0.74476	0.35174	0.81942	65	O9	0.01537	0.14627	0.98364
18	H2	0.54757	0.32124	0.89996	66	O10	0.99097	0.64802	0.01371
19	H3	0.79124	0.35510	0.94586	67	O11	0.51083	0.64801	0.51350
20	H4	0.73594	0.21948	0.88672	68	O12	0.48438	0.14632	0.48369
21	H5	0.28696	0.86832	0.05115	69	O13	0.03020	0.38082	0.01271
22	H6	0.15291	0.84955	0.15991	70	O14	0.96733	0.88229	0.98464
23	H7	0.40717	0.85177	0.16826	71	O15	0.53439	0.88243	0.48466
24	H8	0.29101	0.72950	0.11203	72	O16	0.46884	0.38091	0.51288

25	H9	0.21268	0.86860	0.55059	73	O17	0.28694	0.28155	0.89787
26	H10	0.09342	0.85135	0.66768	74	O18	0.71614	0.77935	0.10060
27	H11	0.34767	0.84983	0.65927	75	O19	0.78537	0.77912	0.60071
28	H12	0.21028	0.72954	0.61126	76	O20	0.21342	0.28186	0.39724
29	H13	0.72370	0.34818	0.32186	77	O21	0.30070	0.23828	0.08995
30	H14	0.71524	0.35362	0.44943	78	O22	0.70249	0.73780	0.90751
31	H15	0.94665	0.32885	0.38888	79	O23	0.79908	0.73782	0.40757
32	H16	0.77038	0.21839	0.38938	80	O24	0.19893	0.23834	0.58968
33	H17	0.88045	0.06418	0.52999	81	O25	0.17869	0.54397	0.61461
34	H18	0.67133	0.10060	0.59274	82	O26	0.81887	0.04214	0.38293
35	H19	0.89837	0.09726	0.65954	83	O27	0.68297	0.04161	0.88241
36	H20	0.78702	0.96420	0.61606	84	O28	0.32281	0.54341	0.11464
37	H21	0.10829	0.60242	0.33639	85	O29	0.80015	0.48671	0.61303
38	H22	0.10875	0.56517	0.46482	86	O30	0.19613	0.98363	0.38445
39	H23	0.32599	0.60811	0.41280	87	O31	0.30555	0.98387	0.88445
40	H24	0.22221	0.47093	0.38081	88	O32	0.70157	0.48712	0.11309
41	H25	0.39268	0.60226	0.83616	89	O33	0.94853	0.62211	0.75455
42	H26	0.17549	0.60777	0.91267	90	O34	0.05124	0.12418	0.24427
43	H27	0.39322	0.56491	0.96451	91	O35	0.44977	0.12419	0.74413
44	H28	0.27950	0.47066	0.88047	92	O36	0.55262	0.62235	0.25448
45	H29	0.61587	0.06450	0.03024	93	O37	0.03003	0.39215	0.74665
46	H30	0.60396	0.09788	0.15964	94	O38	0.96382	0.89487	0.24860
47	H31	0.82808	0.10148	0.09006	95	O39	0.53673	0.89465	0.74829
48	H32	0.71434	0.96492	0.11524	96	O40	0.47333	0.39196	0.24681

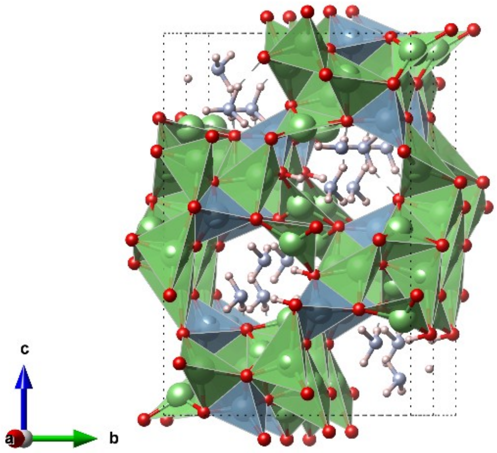

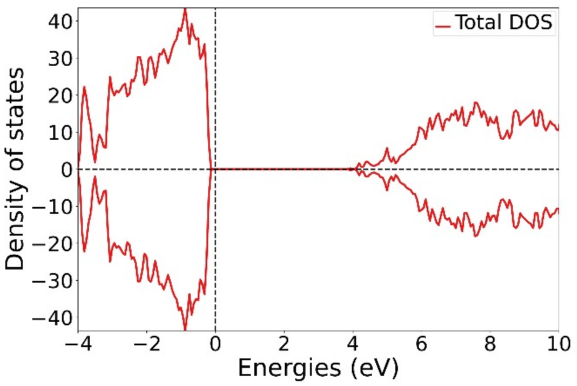

Figure S10. Structure diagram and density of states of $NH_4Li_4(AlO_4)$.

Table S16. Cell parameters of $NH_4Li_4(AlO_4)$, ICSD is the ICSD Collection Code, STRU is the chemical formula. The a, b, c (Å) and α, β, γ (°) are unit cell parameters. V (Å^3) is the cell volume and Structure parameters of $NH_4Li_4(AlO_4)$.

ICSD	STRU	a	b	c	α	β	γ	V
65260	$NH_4Li_4(AlO_4)$	5.60	10.66	16.47	90.01	90.00	100.22	968.35

NO.	E	x	y	z	NO.	E	x	y	z
1	Li1	0.32346	0.22544	0.61889	57	H17	0.17925	0.78758	0.22039
2	Li2	0.18713	0.26915	0.11623	58	H18	0.30264	0.74581	0.13690
3	Li3	0.68726	0.76894	0.38381	59	H19	0.45581	0.75252	0.22268
4	Li4	0.82376	0.72536	0.88115	60	H20	0.48294	0.94640	0.19664
5	Li5	0.11908	0.41486	0.01649	61	H21	0.96714	0.72666	0.71976
6	Li6	0.38441	0.07533	0.52358	62	H22	0.18237	0.77067	0.64109
7	Li7	0.88466	0.57541	0.97653	63	H23	0.19321	0.64422	0.70354
8	Li8	0.61902	0.91503	0.48336	64	H24	0.26265	0.80447	0.73716
9	Li9	0.17339	0.03160	0.74874	65	H25	0.46658	0.22667	0.78031
10	Li10	0.34637	0.45495	0.24698	66	H26	0.76231	0.30422	0.76296
11	Li11	0.84667	0.95433	0.25280	67	H27	0.69230	0.14395	0.79651
12	Li12	0.67344	0.53136	0.75157	68	H28	0.68192	0.27044	0.85899
13	Li13	0.78007	0.13084	0.11775	69	H29	0.67515	0.28843	0.28024
14	Li14	0.72575	0.36346	0.62276	70	H30	-0.04984	0.25175	0.27699
15	Li15	0.22599	0.86345	0.87745	71	H31	0.79952	0.24543	0.36322
16	Li16	0.27954	0.63061	0.38244	72	H32	0.98233	0.44603	0.30347
17	Li17	0.51678	0.45560	0.91734	73	N1	0.63606	0.64197	0.59440
18	Li18	0.98232	0.04285	0.42091	74	N2	0.85610	0.84855	0.09386
19	Li19	0.48239	0.54264	0.07901	75	N3	0.35205	0.34895	0.40565
20	Li20	0.01650	0.95566	0.58281	76	N4	0.13599	0.14142	0.90620
21	Li21	0.34904	0.01012	0.04778	77	N5	0.34180	0.79785	0.18946
22	Li22	0.14496	0.47754	0.54903	78	N6	0.14827	0.73291	0.69944
23	Li23	0.64516	0.97713	0.95119	79	N7	0.64775	0.23273	0.80063
24	Li24	0.84759	0.51013	0.45273	80	N8	0.83838	0.29774	0.31073

25	Li25	0.91268	0.13437	0.66589	81	O1	0.68560	0.71310	0.77060
26	Li26	0.60246	0.36328	0.15834	82	O2	0.85272	0.77710	0.27676
27	Li27	0.10259	0.86328	0.34172	83	O3	0.35189	0.27745	0.22326
28	Li28	0.41284	0.63431	0.83424	84	O4	0.18565	0.21317	0.72951
29	Li29	0.96040	0.51069	0.13592	85	O5	0.41284	0.92742	0.77644
30	Li30	0.53773	0.98488	0.64652	86	O6	0.06540	0.53803	0.28834
31	Li31	0.03777	0.48482	0.85358	87	O7	0.56558	0.03854	0.21167
32	Li32	0.46058	0.01087	0.36403	88	O8	0.91276	0.42742	0.72383
33	Al1	0.82133	0.19602	0.52208	89	O9	0.61255	0.57323	0.36492
34	Al2	0.67835	0.29596	0.01667	90	O10	0.87193	0.91806	0.86730
35	Al3	0.17819	0.79604	0.48334	91	O11	0.37195	0.41833	0.63292
36	Al4	0.32154	0.69584	0.97804	92	O12	0.11285	0.07347	0.13501
37	Al5	0.79454	0.61121	0.27259	93	O13	0.86557	0.96003	0.69025
38	Al6	0.70202	0.88033	0.77194	94	O14	0.64572	0.53790	0.18677
39	Al7	0.20199	0.38037	0.72822	95	O15	0.14555	0.03790	0.31315
40	Al8	0.29436	0.11154	0.22745	96	O16	0.36558	0.45989	0.80999
41	H1	0.52296	0.68375	0.55845	97	O17	0.42008	0.36443	0.04128
42	H2	0.79958	0.64846	0.56454	98	O18	0.08647	0.13133	0.54423
43	H3	0.47829	0.50498	0.61924	99	O19	0.58685	0.63135	0.95586
44	H4	0.66282	0.69439	0.64735	100	O20	0.92019	0.86469	0.45868
45	H5	0.69463	0.84346	0.06295	101	O21	0.66343	0.09668	0.44447
46	H6	0.97119	0.80842	0.05763	102	O22	0.83099	0.39148	0.93647
47	H7	0.82216	0.79158	0.14444	103	O23	0.33089	0.89144	0.56361
48	H8	0.01312	0.98493	0.12058	104	O24	0.16378	0.59638	0.05558
49	H9	0.51209	0.48487	0.37934	105	O25	0.87315	0.36330	0.51250
50	H10	0.31707	0.29292	0.35466	106	O26	0.61913	0.12822	0.00847
51	H11	0.19059	0.34478	0.43647	107	O27	0.11819	0.62834	0.49160
52	H12	0.46497	0.30705	0.44167	108	O28	0.37301	0.86313	0.98783
53	H13	0.02242	0.18208	0.94266	109	O29	0.62361	0.16653	0.60905
54	H14	0.16224	0.19488	0.85371	110	O30	0.88286	0.32527	0.10239
55	H15	0.97844	0.00468	0.88100	111	O31	0.38300	0.82531	0.39767
56	H16	0.29969	0.14774	0.93594	112	O32	0.12392	0.66653	0.89105

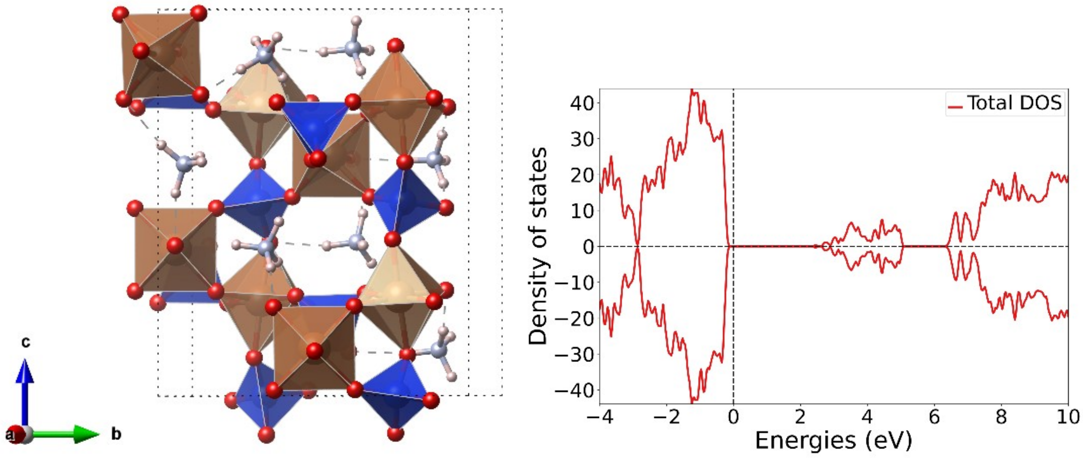

Figure S11. Structure diagram and density of states of $NH_4(SbO)(SiO_4)$.

Table S17. Cell parameters of $NH_4(SbO)(SiO_4)$, ICSD is the ICSD Collection Code, STRU is the chemical formula. The a, b, c (Å) and α, β, γ (°) are unit cell parameters. V (Å^3) is the cell volume and Structure parameters of $NH_4(SbO)(SiO_4)$.

ICSD	STRU	a	b	c	α	β	γ	V
69429	$NH_4(SbO)(SiO_4)$	6.65	10.83	13.27	89.86	89.90	90.13	954.77

NO.	E	x	y	z	NO.	E	x	y	z
1	Si1	0.16366	0.25180	0.00167	49	N1	0.72197	0.31347	0.87977
2	Si2	0.83168	0.75110	0.00160	50	N2	0.27776	0.82558	0.11790
3	Si3	0.66762	0.75211	0.50133	51	N3	0.21509	0.80499	0.60980
4	Si4	0.33304	0.25031	0.50071	52	N4	0.77741	0.32362	0.38151
5	Si5	0.99868	0.50572	0.68396	53	N5	0.83911	0.06786	0.59976
6	Si6	0.00167	0.00313	0.31808	54	N6	0.18917	0.55137	0.39765
7	Si7	0.48878	0.00172	0.81932	55	N7	0.31543	0.55305	0.89800
8	Si8	0.50348	0.50440	0.18455	56	N8	0.68362	0.05169	0.10220
9	Sb1	0.99246	0.00425	0.88122	57	O1	0.01178	0.13303	0.98943
10	Sb2	0.99914	0.50388	0.12210	58	O2	0.98596	0.63357	0.01576
11	Sb3	0.50428	0.50157	0.62109	59	O3	0.52007	0.63108	0.51435
12	Sb4	0.49592	0.00217	0.37959	60	O4	0.48347	0.13133	0.48622
13	Sb5	0.24530	0.25163	0.74883	61	O5	0.02437	0.37762	0.01436
14	Sb6	0.75351	0.74931	0.25575	62	O6	0.96881	0.87663	0.98682
15	Sb7	0.74292	0.75267	0.75513	63	O7	0.52144	0.87420	0.48579
16	Sb8	0.25279	0.24945	0.24689	64	O8	0.47260	0.37482	0.51544
17	H1	0.65357	0.36423	0.82270	65	O9	0.30344	0.27342	0.89991
18	H2	0.60590	0.27197	0.91845	66	O10	0.69765	0.77069	0.10530
19	H3	0.81385	0.36383	0.92779	67	O11	0.80039	0.77377	0.60426
20	H4	0.80977	0.24732	0.84471	68	O12	0.19838	0.27225	0.39770
21	H5	0.28438	0.86776	0.04851	69	O13	0.31178	0.23462	0.09936
22	H6	0.14225	0.84806	0.15301	70	O14	0.68629	0.73115	0.90316
23	H7	0.39231	0.85253	0.16710	71	O15	0.81598	0.73606	0.40337

24	H8	0.28534	0.73102	0.10926	72	O16	0.18393	0.23131	0.59949
25	H9	0.29306	0.84196	0.54876	73	O17	0.19825	0.53781	0.61294
26	H10	0.06238	0.82271	0.60855	74	O18	0.80236	0.03363	0.38904
27	H11	0.28407	0.83714	0.67441	75	O19	0.68672	0.03271	0.88994
28	H12	0.22841	0.70952	0.60929	76	O20	0.30476	0.53812	0.11492
29	H13	0.89126	0.35099	0.33155	77	O21	0.80428	0.47574	0.61082
30	H14	0.64146	0.34574	0.34667	78	O22	0.19725	0.97633	0.39001
31	H15	0.78462	0.36621	0.45043	79	O23	0.29217	0.97810	0.89240
32	H16	0.78562	0.22922	0.39097	80	O24	0.69989	0.47889	0.11276
33	H17	0.83069	0.03830	0.52527	81	O25	0.95599	0.62355	0.75964
34	H18	0.70038	0.10239	0.61973	82	O26	0.04202	0.12004	0.24090
35	H19	0.95028	0.13523	0.60530	83	O27	0.44849	0.11802	0.74084
36	H20	0.87475	0.99722	0.64923	84	O28	0.54503	0.61835	0.26446
37	H21	0.09207	0.58852	0.34338	85	O29	0.04850	0.38878	0.75856
38	H22	0.14476	0.56665	0.47120	86	O30	0.95580	0.88559	0.24273
39	H23	0.33165	0.58794	0.38895	87	O31	0.52768	0.88117	0.74644
40	H24	0.19994	0.45687	0.38809	88	O32	0.45673	0.38459	0.25821
41	H25	0.40825	0.58879	0.84089	89	O33	0.52780	0.62783	0.72985
42	H26	0.17291	0.59010	0.89362	90	O34	0.47370	0.12915	0.27085
43	H27	0.36858	0.56969	0.96962	91	O35	0.01427	0.13999	0.77943
44	H28	0.30212	0.45850	0.88974	92	O36	0.97499	0.62961	0.23160
45	H29	0.63072	0.06220	0.02939	93	O37	0.46703	0.37480	0.72793
46	H30	0.59088	0.08979	0.15772	94	O38	0.53678	0.87599	0.27250
47	H31	0.82521	0.09112	0.10343	95	O39	0.95162	0.88551	0.76857
48	H32	0.70040	0.95818	0.11514	96	O40	0.03762	0.37640	0.22888

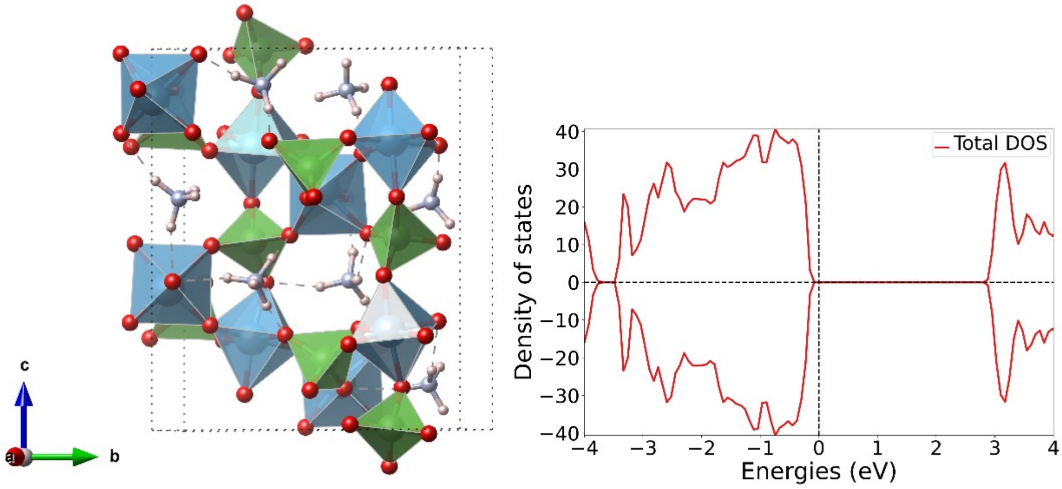

Figure S12. Structure diagram and density of states of $NH_4(TiO)(AsO_4)$.

Table S18. Cell parameters of $NH_4(TiO)(AsO_4)$, ICSD is the ICSD Collection Code, STRU is the chemical formula. The a, b, c (Å) and α, β, γ (°) are unit cell parameters. V (Å^3) is the cell volume and Structure parameters of $NH_4(TiO)(AsO_4)$.

ICSD	STRU	a	b	c	α	β	γ	V
74591	$NH_4(TiO)(AsO_4)$	6.75	10.97	13.37	89.75	89.69	89.63	989.23

NO.	E	x	y	z	NO.	E	x	y	z
1	Ti1	0.98076	0.00376	0.86805	49	N1	0.71308	0.32953	0.90445
2	Ti2	0.02006	0.50539	0.12750	50	N2	0.28473	0.82498	0.11896
3	Ti3	0.48548	0.50266	0.63062	51	N3	0.20958	0.82526	0.59703
4	Ti4	0.50727	0.00069	0.36983	52	N4	0.78456	0.31412	0.38933
5	Ti5	0.22546	0.24964	0.74237	53	N5	0.82101	0.06908	0.60721
6	Ti6	0.77374	0.74680	0.25809	54	N6	0.18900	0.55623	0.39315
7	Ti7	0.72243	0.74899	0.75762	55	N7	0.31263	0.56095	0.89138
8	Ti8	0.27397	0.24863	0.23961	56	N8	0.68703	0.05534	0.10489
9	As1	0.16457	0.25957	0.99586	57	O1	0.49446	0.14095	0.48426
10	As2	0.84018	0.76022	0.00261	58	O2	0.49050	0.64782	0.51493
11	As3	0.66427	0.75912	0.50363	59	O3	0.00756	0.64454	0.01263
12	As4	0.33499	0.25980	0.49616	60	O4	0.98686	0.15097	0.98504
13	As5	0.98201	0.51126	0.68270	61	O5	0.05124	0.39962	0.00869
14	As6	0.01246	0.01081	0.31816	62	O6	0.96508	0.89554	0.98766
15	As7	0.47972	0.01001	0.81718	63	O7	0.54405	0.89712	0.49038
16	As8	0.52216	0.51019	0.18045	64	O8	0.46721	0.39258	0.51119
17	H1	0.79406	0.35801	0.84237	65	O9	0.30912	0.27795	0.88944
18	H2	0.56439	0.31369	0.88866	66	O10	0.70142	0.78058	0.11060
19	H3	0.72865	0.39207	0.96130	67	O11	0.80693	0.77421	0.60961
20	H4	0.77941	0.24883	0.92886	68	O12	0.19739	0.28616	0.38862
21	H5	0.26799	0.86970	0.05154	69	O13	0.17174	0.23770	0.59550
22	H6	0.16401	0.84766	0.16521	70	O14	0.82235	0.73431	0.40316
23	H7	0.41458	0.84730	0.15481	71	O15	0.68027	0.73759	0.90365

24	H8	0.28634	0.73149	0.10718	72	O16	0.32192	0.23015	0.09494
25	H9	0.23003	0.88743	0.53986	73	O17	0.68131	0.04578	0.88988
26	H10	0.05933	0.81048	0.61121	74	O18	0.32089	0.55350	0.10946
27	H11	0.28712	0.85360	0.65952	75	O19	0.18437	0.54564	0.60906
28	H12	0.27520	0.74400	0.57402	76	O20	0.80804	0.04705	0.38991
29	H13	0.70764	0.34179	0.32593	77	O21	0.72320	0.48460	0.10382
30	H14	0.72353	0.36044	0.44960	78	O22	0.27898	0.98412	0.89335
31	H15	0.93689	0.32442	0.38271	79	O23	0.21174	0.97992	0.39386
32	H16	0.75923	0.22157	0.39912	80	O24	0.78107	0.48396	0.60801
33	H17	0.80832	0.04198	0.53325	81	O25	0.44701	0.13000	0.73364
34	H18	0.68594	0.10594	0.62866	82	O26	0.55853	0.62670	0.26496
35	H19	0.93267	0.13289	0.61303	83	O27	0.94488	0.63135	0.76436
36	H20	0.85276	0.99608	0.65410	84	O28	0.05120	0.13234	0.23812
37	H21	0.09566	0.59681	0.34076	85	O29	0.01914	0.38390	0.75675
38	H22	0.13624	0.56273	0.46590	86	O30	0.97203	0.88542	0.24415
39	H23	0.32735	0.59625	0.39325	87	O31	0.51546	0.88204	0.74446
40	H24	0.20673	0.46449	0.37661	88	O32	0.48494	0.37952	0.24965
41	H25	0.39984	0.60163	0.83596	89	O33	0.42402	0.38748	0.71666
42	H26	0.17411	0.60162	0.89827	90	O34	0.56816	0.88393	0.28356
43	H27	0.37481	0.56629	0.96160	91	O35	0.92028	0.89278	0.77844
44	H28	0.29232	0.47024	0.87411	92	O36	0.08178	0.38760	0.21270
45	H29	0.62871	0.05983	0.03341	93	O37	0.04524	0.13738	0.77972
46	H30	0.59546	0.09527	0.15860	94	O38	0.96183	0.63487	0.22269
47	H31	0.82336	0.09795	0.09991	95	O39	0.54019	0.63473	0.72378
48	H32	0.71100	0.96443	0.12256	96	O40	0.45496	0.13207	0.27604

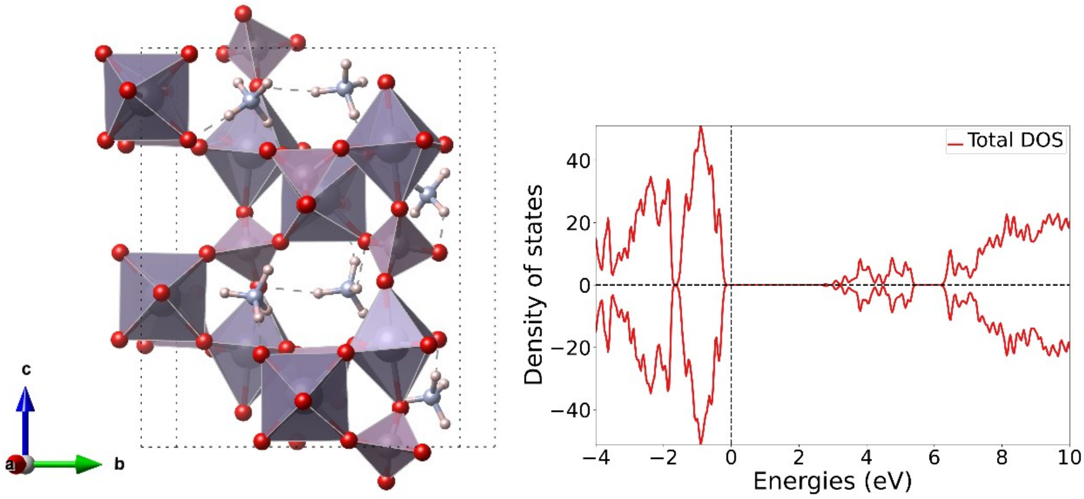

Figure S13. Structure diagram and density of states of $NH_4SnO(PO_4)$.

Table S19. Cell parameters of $NH_4SnO(PO_4)$, ICSD is the ICSD Collection Code, STRU is the chemical formula. The a, b, c (Å) and α, β, γ (°) are unit cell parameters. V (Å^3) is the cell volume and Structure parameters of $NH_4SnO(PO_4)$.

ICSD	STRU	a	b	c	α	β	γ	V
79650	$NH_4(SnO)(PO_4)$	6.71	10.92	13.43	90.00	90.00	89.87	983.87

NO.	E	x	y	z	NO.	E	x	y	z
1	Sn1	0.99399	0.01314	0.87463	49	N1	0.71884	0.31458	0.86823
2	Sn2	0.99915	0.51249	0.12764	50	N2	0.28400	0.83347	0.12447
3	Sn3	0.49395	0.51315	0.62550	51	N3	0.21889	0.81456	0.63193
4	Sn4	0.49910	0.01245	0.37249	52	N4	0.78393	0.33333	0.37559
5	Sn5	0.23932	0.26033	0.74302	53	N5	0.82711	0.06546	0.60128
6	Sn6	0.75672	0.75717	0.25808	54	N6	0.18570	0.56221	0.39507
7	Sn7	0.73934	0.76033	0.75709	55	N7	0.32702	0.56545	0.89883
8	Sn8	0.25672	0.25718	0.24204	56	N8	0.68570	0.06225	0.10507
9	P1	0.15174	0.26465	0.99755	57	O1	0.00949	0.15263	0.98520
10	P2	0.84545	0.76233	0.00246	58	O2	0.99201	0.65152	0.01617
11	P3	0.65179	0.76461	0.50257	59	O3	0.50949	0.65260	0.51491
12	P4	0.34545	0.26230	0.49767	60	O4	0.49198	0.15147	0.48396
13	P5	0.98924	0.51356	0.67620	61	O5	0.01891	0.38158	0.01338
14	P6	0.00451	0.01443	0.32009	62	O6	0.97358	0.87985	0.98594
15	P7	0.48926	0.01353	0.82398	63	O7	0.51901	0.88156	0.48677
16	P8	0.50453	0.51446	0.18002	64	O8	0.47360	0.37982	0.51420
17	H1	0.62625	0.35699	0.81349	65	O9	0.27915	0.28700	0.90279
18	H2	0.62826	0.26343	0.91313	66	O10	0.71837	0.78299	0.09749
19	H3	0.80114	0.37389	0.91124	67	O11	0.77923	0.78689	0.59733
20	H4	0.81729	0.25659	0.83099	68	O12	0.21837	0.28296	0.40265
21	H5	0.29605	0.87666	0.05703	69	O13	0.29495	0.24297	0.08597
22	H6	0.14943	0.85669	0.15752	70	O14	0.70481	0.73720	0.91327

23	H7	0.39690	0.85817	0.17727	71	O15	0.79500	0.74294	0.41416
24	H8	0.28826	0.74010	0.11401	72	O16	0.20478	0.23718	0.58685
25	H9	0.12829	0.76342	0.58703	73	O17	0.17271	0.54074	0.60818
26	H10	0.12630	0.85697	0.68666	74	O18	0.81923	0.04406	0.38598
27	H11	0.30120	0.87387	0.58892	75	O19	0.67275	0.04070	0.89197
28	H12	0.31733	0.75656	0.66916	76	O20	0.31927	0.54408	0.11411
29	H13	0.89688	0.35811	0.32284	77	O21	0.80697	0.49283	0.60649
30	H14	0.64940	0.35651	0.34248	78	O22	0.18537	0.99518	0.39047
31	H15	0.79581	0.37652	0.44304	79	O23	0.30700	0.99278	0.89370
32	H16	0.78831	0.23996	0.38604	80	O24	0.68541	0.49523	0.10967
33	H17	0.91754	0.07886	0.54063	81	O25	0.95386	0.62227	0.74882
34	H18	0.69086	0.10523	0.58516	82	O26	0.03950	0.12063	0.24521
35	H19	0.89365	0.10204	0.66678	83	O27	0.45383	0.12228	0.75139
36	H20	0.80639	0.97232	0.60988	84	O28	0.53948	0.62065	0.25491
37	H21	0.09762	0.59825	0.33548	85	O29	0.02632	0.40036	0.74266
38	H22	0.12285	0.57449	0.46436	86	O30	0.96925	0.89914	0.25463
39	H23	0.32445	0.60230	0.39642	87	O31	0.52631	0.90037	0.75748
40	H24	0.20491	0.46923	0.38497	88	O32	0.46925	0.39916	0.24549
41	H25	0.39368	0.60204	0.83338	89	O33	0.00568	0.14850	0.77002
42	H26	0.19074	0.60523	0.91488	90	O34	0.98150	0.64080	0.23581
43	H27	0.41728	0.57883	0.95956	91	O35	0.50567	0.64852	0.73009
44	H28	0.30632	0.47231	0.89022	92	O36	0.48148	0.14079	0.26431
45	H29	0.62278	0.07448	0.03579	93	O37	0.45893	0.38482	0.73138
46	H30	0.59763	0.09828	0.16467	94	O38	0.53802	0.88136	0.26876
47	H31	0.82441	0.10239	0.10367	95	O39	0.95896	0.88481	0.76875
48	H32	0.70501	0.96929	0.11519	96	O40	0.03804	0.38138	0.23137

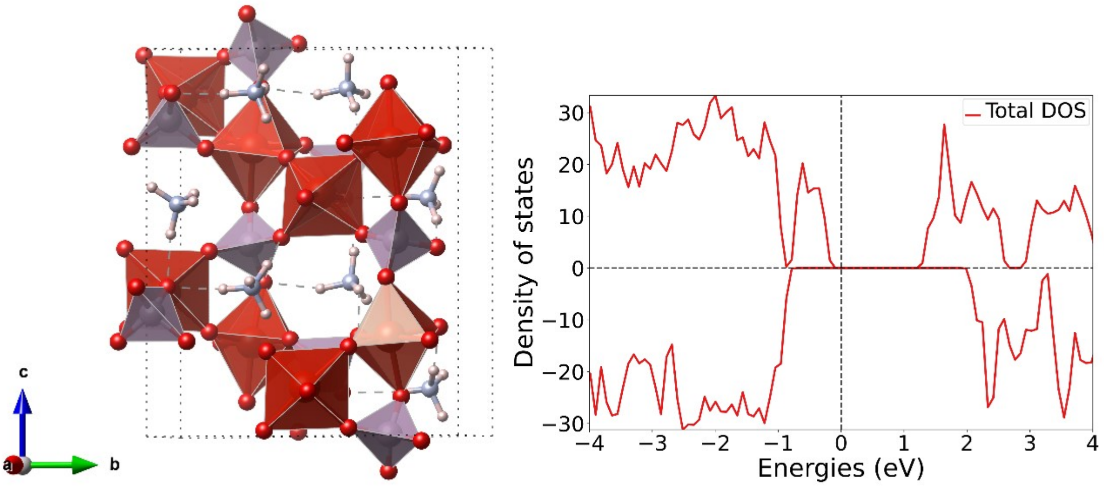

Figure S14. Structure diagram and density of states of $NH_4(VO)(PO_4)$.

Table S20. Cell parameters of $NH_4(VO)(PO_4)$, ICSD is the ICSD Collection Code, STRU is the chemical formula. The a, b, c (Å) and α, β, γ (°) are unit cell parameters. V (Å^3) is the cell volume and Structure parameters of $NH_4(VO)(PO_4)$.

ICSD	STRU	a	b	c	α	β	γ	V
79651	$NH_4VO(PO_4)$	6.55	10.64	13.04	89.70	89.64	90.11	909.35

NO.	E	x	y	z	NO.	E	x	y	z
1	V1	0.00837	0.00406	0.87349	49	N1	0.71145	0.31848	0.88720
2	V2	0.99299	0.50643	0.12684	50	N2	0.27960	0.83058	0.12188
3	V3	0.51204	0.50394	0.62720	51	N3	0.20761	0.81668	0.61404
4	V4	0.48479	0.00289	0.37193	52	N4	0.78897	0.31621	0.38428
5	V5	0.23505	0.25701	0.74588	53	N5	0.82818	0.08107	0.59910
6	V6	0.76474	0.75473	0.25518	54	N6	0.19294	0.56657	0.39783
7	V7	0.73392	0.75678	0.75536	55	N7	0.30886	0.56771	0.89725
8	V8	0.26483	0.25716	0.24327	56	N8	0.68709	0.06291	0.10044
9	P1	0.16133	0.26274	0.99769	57	O1	0.02640	0.14542	0.98106
10	P2	0.83823	0.76219	0.00304	58	O2	0.97653	0.64575	0.02033
11	P3	0.66181	0.76266	0.50293	59	O3	0.52790	0.64427	0.52048
12	P4	0.33694	0.26104	0.49683	60	O4	0.46881	0.14274	0.47844
13	P5	0.00239	0.51819	0.68248	61	O5	0.02165	0.37970	0.01406
14	P6	0.99438	0.01761	0.31651	62	O6	0.97595	0.87972	0.98580
15	P7	0.50149	0.01744	0.81786	63	O7	0.51809	0.87808	0.48602
16	P8	0.50000	0.51877	0.18203	64	O8	0.48018	0.37621	0.51390
17	H1	0.76147	0.35844	0.81971	65	O9	0.29399	0.28945	0.89968
18	H2	0.55576	0.32969	0.89798	66	O10	0.70690	0.78537	0.10147
19	H3	0.80016	0.35444	0.94643	67	O11	0.79280	0.78785	0.60006
20	H4	0.73502	0.22227	0.88286	68	O12	0.20618	0.28881	0.39927
21	H5	0.28562	0.87197	0.05061	69	O13	0.30378	0.24609	0.09084
22	H6	0.14548	0.85823	0.15863	70	O14	0.69845	0.74398	0.90892
23	H7	0.40042	0.85585	0.16833	71	O15	0.80284	0.74756	0.40831
24	H8	0.28130	0.73367	0.11413	72	O16	0.19319	0.24387	0.59086

25	H9	0.30397	0.85475	0.55772	73	O17	0.19333	0.54714	0.61381
26	H10	0.05292	0.82391	0.59777	74	O18	0.80301	0.04453	0.38506
27	H11	0.23999	0.85711	0.68321	75	O19	0.69064	0.04797	0.88604
28	H12	0.23647	0.72113	0.61985	76	O20	0.31249	0.55183	0.11381
29	H13	0.75213	0.35644	0.31523	77	O21	0.81499	0.48913	0.61407
30	H14	0.69468	0.35371	0.44134	78	O22	0.18135	0.98715	0.38463
31	H15	0.94386	0.32502	0.39874	79	O23	0.31307	0.98873	0.88742
32	H16	0.76095	0.22053	0.37955	80	O24	0.68999	0.49127	0.11437
33	H17	0.81166	0.05697	0.52214	81	O25	0.96390	0.63235	0.75422
34	H18	0.69317	0.11857	0.62632	82	O26	0.03322	0.13253	0.24497
35	H19	0.94814	0.14416	0.60561	83	O27	0.46395	0.13091	0.74448
36	H20	0.85812	0.00243	0.64277	84	O28	0.53809	0.62953	0.25760
37	H21	0.07797	0.60538	0.35381	85	O29	0.04444	0.40300	0.75246
38	H22	0.16797	0.57476	0.47636	86	O30	0.94968	0.90403	0.24537
39	H23	0.33063	0.60782	0.37840	87	O31	0.54531	0.90209	0.74931
40	H24	0.20652	0.47181	0.38185	88	O32	0.45470	0.40174	0.24990
41	H25	0.42031	0.60428	0.84869	89	O33	0.05413	0.14435	0.77530
42	H26	0.17118	0.61116	0.88363	90	O34	0.95025	0.64284	0.22915
43	H27	0.34369	0.57640	0.97449	91	O35	0.55347	0.64122	0.72842
44	H28	0.28828	0.47317	0.88240	92	O36	0.44395	0.14065	0.27081
45	H29	0.65466	0.07234	0.02282	93	O37	0.45276	0.39013	0.71776
46	H30	0.57726	0.10163	0.14821	94	O38	0.54342	0.88954	0.28065
47	H31	0.82670	0.10418	0.11411	95	O39	0.95128	0.89403	0.78018
48	H32	0.70242	0.96765	0.11541	96	O40	0.05061	0.39189	0.21731

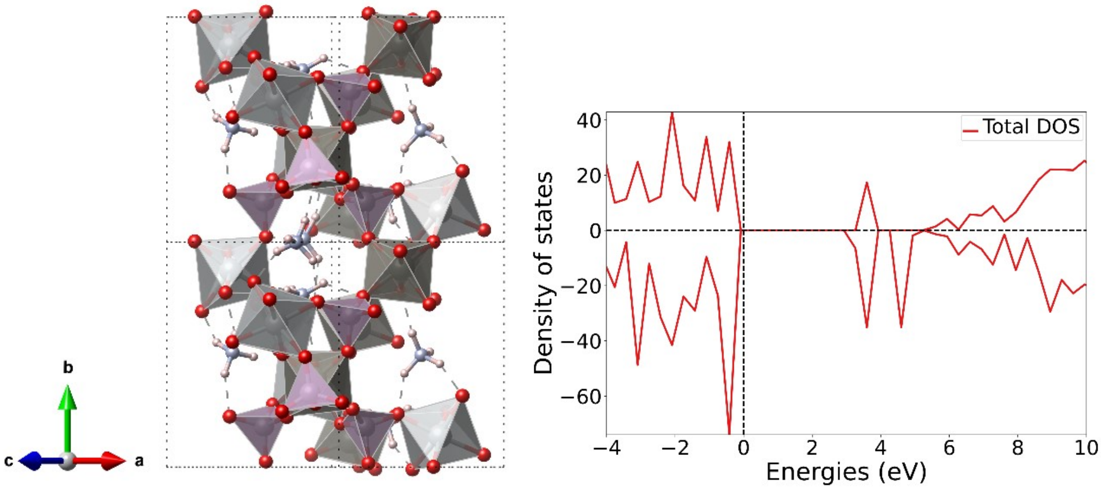

Figure S15. Structure diagram and density of states of $(NH_4)_2Ni(WO_2(PO_4)_2)$.

Table S21. Cell parameters of $(NH_4)_2Ni(WO_2(PO_4)_2)$, ICSD is the ICSD Collection Code, STRU is the chemical formula. The a, b, c (Å) and α, β, γ (°) are unit cell parameters. V (Å^3) is the cell volume and Structure parameters of $(NH_4)_2Ni(WO_2(PO_4)_2)$.

ICSD	STRU	a	b	c	α	β	γ	V
79702	$(NH_4)_2Ni(WO_2(PO_4)_2)$	9.49	9.43	10.77	90.14	89.77	90.07	964.34

NO.	E	x	y	z	NO.	E	x	y	z
1	Ni1	0.13117	0.87555	0.74826	49	N1	0.49077	0.76439	0.69027
2	Ni2	0.87161	0.13672	0.25383	50	N2	0.50846	0.19959	0.19468
3	Ni3	0.37746	0.37690	0.50108	51	N3	0.26211	0.01377	0.44397
4	Ni4	0.63371	0.63712	0.99874	52	N4	0.70162	0.99835	0.94817
5	P1	0.82554	0.67570	0.74723	53	N5	0.19361	0.50897	0.80309
6	P2	0.18479	0.33090	0.24926	54	N6	0.80867	0.49756	0.30206
7	P3	0.17581	0.67974	0.49627	55	N7	0.01054	0.26693	0.56271
8	P4	0.83107	0.32846	-0.00103	56	N8	-0.00134	0.70428	0.05439
9	P5	0.32699	0.18144	0.75148	57	O1	0.72053	0.25683	0.62596
10	P6	0.67555	0.82927	0.25146	58	O2	0.28324	0.74729	0.12072
11	P7	0.67450	0.17738	0.50348	59	O3	0.75792	0.78095	0.37080
12	P8	0.32432	0.83124	0.00019	60	O4	0.24383	0.22405	0.87261
13	H1	0.39214	0.78919	0.65230	61	O5	0.74691	0.28093	0.88014
14	H2	0.50586	0.65677	0.67505	62	O6	0.25901	0.72558	0.37582
15	H3	0.49090	0.78561	0.78450	63	O7	0.78080	0.75625	0.62570
16	H4	0.57275	0.81745	0.64853	64	O8	0.22270	0.24704	0.12780
17	H5	0.41720	0.19813	0.14278	65	O9	0.78643	0.51441	0.72237
18	H6	0.55408	0.09951	0.20743	66	O10	0.22421	0.49090	0.22015
19	H7	0.48360	0.23926	0.28127	67	O11	0.01591	0.72303	0.46998
20	H8	0.58821	0.25828	0.15340	68	O12	0.98962	0.28568	0.97076
21	H9	0.15465	0.00441	0.43006	69	O13	0.48656	0.22230	0.78053

22	H10	0.30785	0.92606	0.40327	70	O14	0.51574	0.79075	0.27818
23	H11	0.28420	0.01444	0.53822	71	O15	0.71794	0.01584	0.52731
24	H12	0.29307	0.10986	0.40405	72	O16	0.28171	0.99068	0.02943
25	H13	0.59901	0.95834	0.95644	73	O17	0.27652	0.27702	0.64488
26	H14	0.75546	0.91708	0.90341	74	O18	0.73052	0.74497	0.13953
27	H15	0.74006	0.01295	0.03649	75	O19	0.76362	0.23437	0.39505
28	H16	0.70754	0.09387	0.90120	76	O20	0.23703	0.77545	0.89094
29	H17	0.09275	0.55278	0.79339	77	O21	0.73907	0.73327	0.85668
30	H18	0.19624	0.41477	0.85199	78	O22	0.27886	0.27811	0.35506
31	H19	0.24994	0.58950	0.84606	79	O23	0.22705	0.77217	0.60501
32	H20	0.23137	0.48998	0.71472	80	O24	0.77604	0.24588	0.11150
33	H21	0.75361	0.41627	0.34592	81	O25	0.30995	0.02434	0.72208
34	H22	0.91138	0.45824	0.29613	82	O26	0.69209	0.98892	0.23223
35	H23	0.80139	0.59331	0.34880	83	O27	0.51706	0.19243	0.48240
36	H24	0.77275	0.51128	0.21280	84	O28	0.48232	0.82104	0.97348
37	H25	0.92929	0.32253	0.60321	85	O29	0.98382	0.69210	0.76825
38	H26	0.99289	0.16042	0.58020	86	O30	0.02789	0.31603	0.28089
39	H27	0.10999	0.29171	0.59940	87	O31	0.19144	0.52173	0.51978
40	H28	0.01065	0.28535	0.46798	88	O32	0.82034	0.48843	0.01828
41	H29	0.91892	0.76051	0.09824	89	O33	0.50818	0.46160	0.63219
42	H30	0.95469	0.60314	0.04130	90	O34	0.50516	0.55587	0.12833
43	H31	0.09189	0.70427	0.10390	91	O35	0.95503	0.00076	0.38034
44	H32	0.01935	0.74603	0.96802	92	O36	0.04833	0.00322	0.88039
45	W1	0.61771	0.38593	0.75271	93	O37	0.55492	0.50123	0.87443
46	W2	0.39045	0.62012	0.24978	94	O38	0.45840	0.50938	0.37112
47	W3	0.88344	0.88621	0.50030	95	O39	0.99335	0.95921	0.62213
48	W4	0.11488	0.11731	0.00086	96	O40	0.00154	0.05360	0.12306

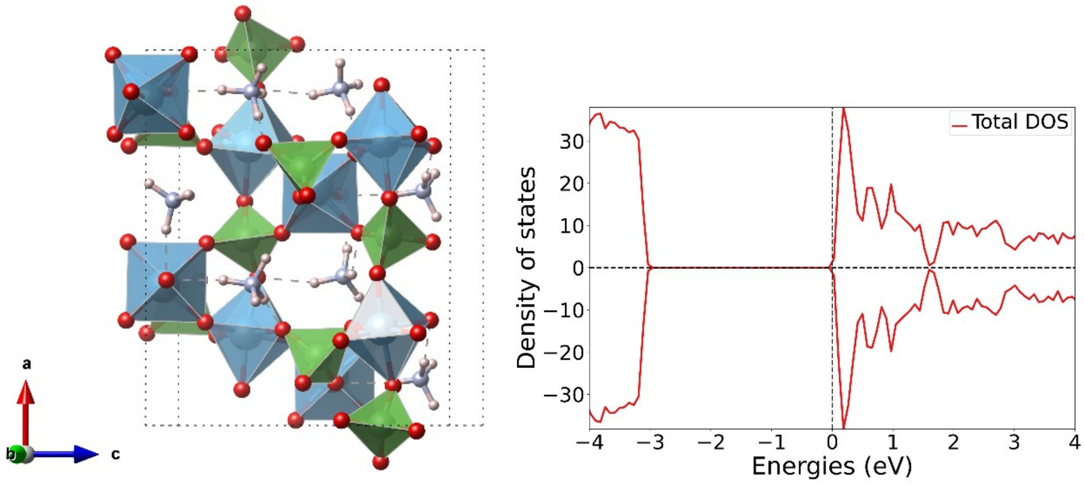

Figure S16. Structure diagram and density of states of $NH_4(TiO)((P_{0.56}As_{0.44})O_4)$.

Table S22. Cell parameters of $NH_4(TiO)((P_{0.56}As_{0.44})O_4)$, ICSD is the ICSD Collection Code, STRU is the chemical formula. The a, b, c (Å) and α, β, γ (°) are unit cell parameters. V (Å^3) is the cell volume and Structure parameters of $NH_4(TiO)((P_{0.56}As_{0.44})O_4)$.

ICSD	STRU	a	b	c	α	β	γ	V
80023	$NH_4(TiO)((P_{0.56}As_{0.44})O_4)$	13.20	6.71	10.85	90.14	89.78	89.61	960.44

NO.	E	x	y	z	NO.	E	x	y	z
1	N1	0.88774	0.71587	0.31546	53	O21	0.71821	0.43178	0.39075
2	N2	0.12346	0.28389	0.82584	54	O22	0.27981	0.55891	0.88802
3	N3	0.62426	0.21658	0.82579	55	O23	0.77540	0.94065	0.89126
4	N4	0.37497	0.78214	0.32681	56	O24	0.22084	0.05981	0.38851
5	N5	0.59852	0.82738	0.07115	57	O25	0.61167	0.18220	0.54991
6	N6	0.39689	0.18524	0.56125	58	O26	0.38689	0.81103	0.04752
7	N7	0.89556	0.31096	0.56422	59	O27	0.88694	0.68920	0.04685
8	N8	0.10268	0.68907	0.05905	60	O28	0.11323	0.31289	0.54924
9	Ti1	0.86875	-0.00025	0.00305	61	O29	0.60917	0.79118	0.48616
10	Ti2	0.13092	0.00234	0.50492	62	O30	0.38882	0.20174	0.98325
11	Ti3	0.62768	0.49295	0.50486	63	O31	0.88806	0.29826	0.98338
12	Ti4	0.37057	0.49945	0.00309	64	O32	0.11282	0.70468	0.48539
13	Ti5	0.74324	0.23277	0.25377	65	O33	0.75938	0.94550	0.62877
14	Ti6	0.25742	0.76595	0.75109	66	O34	0.24027	0.04923	0.13004
15	Ti7	0.75506	0.72955	0.75043	67	O35	0.74120	0.45480	0.13327
16	Ti8	0.24320	0.26806	0.25080	68	O36	0.26008	0.54725	0.63077
17	As1	0.68109	0.98581	0.51282	69	O37	0.75077	0.02464	0.38913
18	As2	0.31739	0.00773	0.01256	70	O38	0.24541	0.96395	0.89208
19	As3	0.81670	0.49380	0.01394	71	O39	0.74405	0.53816	0.89397
20	As4	0.18284	0.50745	0.51388	72	O40	0.25491	0.46239	0.39251
21	As5	0.99837	0.16050	0.26160	73	H1	0.82437	0.79432	0.34604
22	As6	0.00149	0.84099	0.76034	74	H2	0.88324	0.56447	0.33188

23	As7	0.50282	0.65846	0.76122	75	H3	0.95016	0.78192	0.35600
24	As8	0.49657	0.33849	0.25963	76	H4	0.89247	0.73279	0.22047
25	P1	0.68109	0.98581	0.51282	77	H5	0.05424	0.28082	0.86945
26	P2	0.31739	0.00773	0.01256	78	H6	0.16388	0.15487	0.84935
27	P3	0.81670	0.49380	0.01394	79	H7	0.16698	0.40583	0.85102
28	P4	0.18284	0.50745	0.51388	80	H8	0.11304	0.28741	0.73120
29	P5	0.99837	0.16050	0.26160	81	H9	0.55511	0.21600	0.86941
30	P6	0.00149	0.84099	0.76034	82	H10	0.67023	0.09637	0.85081
31	P7	0.50282	0.65846	0.76122	83	H11	0.66226	0.34701	0.85004
32	P8	0.49657	0.33849	0.25963	84	H12	0.61373	0.21258	0.73118
33	O1	0.98732	0.01272	0.14360	85	H13	0.32993	0.90404	0.35157
34	O2	0.01323	0.99197	0.64383	86	H14	0.33553	0.65289	0.35050
35	O3	0.51267	0.50313	0.64618	87	H15	0.44371	0.78013	0.37086
36	O4	0.48456	0.49107	0.14451	88	H16	0.38632	0.78459	0.23234
37	O5	0.01397	0.02819	0.38850	89	H17	0.52169	0.83141	0.04857
38	O6	0.98532	0.97080	0.88829	90	H18	0.61192	0.68956	0.11013
39	O7	0.48785	0.53410	0.89110	91	H19	0.61432	0.94125	0.13237
40	O8	0.51189	0.46308	0.38944	92	H20	0.64360	0.84125	0.99371
41	O9	0.89524	0.29688	0.28772	93	H21	0.34194	0.09578	0.60175
42	O10	0.10512	0.70624	0.78228	94	H22	0.46916	0.12514	0.56629
43	O11	0.60463	0.79589	0.77823	95	H23	0.40182	0.32388	0.60318
44	O12	0.39424	0.20210	0.28072	96	H24	0.37982	0.20715	0.46928
45	O13	0.09551	0.31194	0.24228	97	H25	0.83651	0.39294	0.60425
46	O14	0.90372	0.69100	0.74045	98	H26	0.90770	0.17312	0.60538
47	O15	0.40377	0.80807	0.74043	99	H27	0.96388	0.38379	0.56988
48	O16	0.59528	0.18733	0.23669	100	H28	0.88010	0.28701	0.47197
49	O17	0.72350	0.53758	0.63920	101	H29	0.03125	0.62734	0.06413
50	O18	0.27470	0.45669	0.13693	102	H30	0.15838	0.60072	0.09981
51	O19	0.77965	0.04905	0.13924	103	H31	0.09577	0.82824	0.10032
52	O20	0.22504	0.95636	0.63737	104	H32	0.11976	0.71011	0.96675

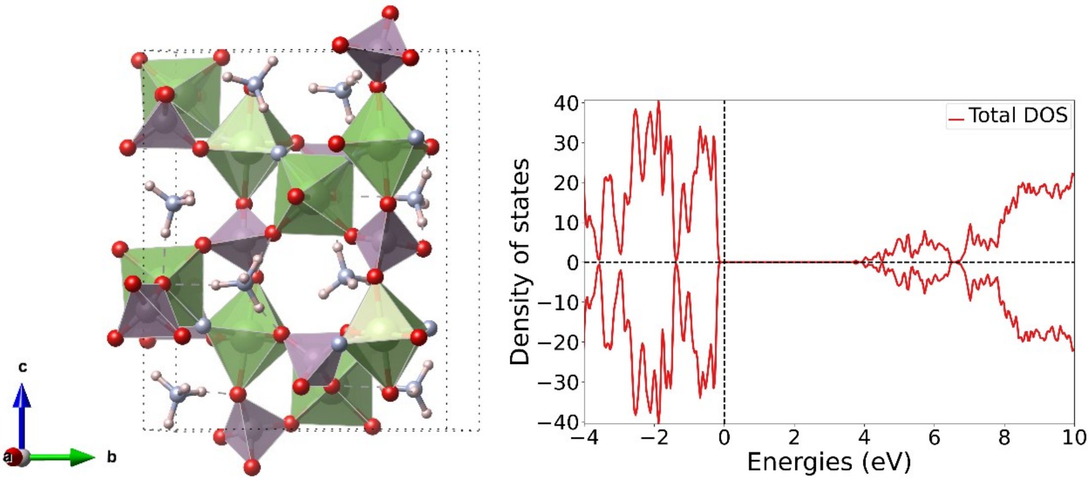

Figure S17. Structure diagram and density of states of $NH_4GaF(PO_4)$.

Table S23. Cell parameters of $NH_4GaF(PO_4)$, ICSD is the ICSD Collection Code, STRU is the chemical formula. The a, b, c (Å) and α, β, γ (°) are unit cell parameters. V (Å^3) is the cell volume and Structure parameters of $NH_4GaF(PO_4)$.

ICSD	STRU	a	b	c	α	β	γ	V
80893	$NH_4GaF(PO_4)$	6.47	10.60	13.06	89.86	89.39	89.86	895.06

NO.	E	x	y	z	NO.	E	x	y	z
1	Ga1	0.00600	0.01571	0.88451	49	N1	0.71348	0.33701	0.90938
2	Ga2	0.00191	0.51444	0.11601	50	N2	0.28640	0.81662	0.10800
3	Ga3	0.51849	0.50809	0.61302	51	N3	0.21488	0.81280	0.60945
4	Ga4	0.48590	0.00978	0.38561	52	N4	0.78493	0.31127	0.38758
5	Ga5	0.25001	0.26378	0.74773	53	N5	0.83881	0.08201	0.59625
6	Ga6	0.75571	0.76299	0.25119	54	N6	0.16369	0.57912	0.40612
7	Ga7	0.74592	0.76389	0.75145	55	N7	0.31961	0.58536	0.89603
8	Ga8	0.25564	0.26250	0.24848	56	N8	0.65673	0.07633	0.09363
9	P1	0.17098	0.26289	0.00219	57	O1	0.01547	0.15268	0.98550
10	P2	0.83461	0.76143	-0.00317	58	O2	0.98310	0.64673	0.01208
11	P3	0.67007	0.75845	0.49614	59	O3	0.53273	0.63990	0.51056
12	P4	0.33207	0.25880	0.50255	60	O4	0.46946	0.14004	0.48861
13	P5	0.00055	0.51355	0.68484	61	O5	0.04134	0.38342	0.01989
14	P6	0.00001	0.01457	0.31435	62	O6	0.97665	0.87779	0.97811
15	P7	0.49830	0.01563	0.81556	63	O7	0.51929	0.87111	0.47727
16	P8	0.50679	0.51526	0.18403	64	O8	0.48114	0.37215	0.51956
17	H1	0.79729	0.37426	0.84860	65	O9	0.30336	0.28333	0.90318
18	H2	0.56035	0.31691	0.89239	66	O10	0.70526	0.78249	0.09581
19	H3	0.71985	0.39632	0.97177	67	O11	0.79672	0.78471	0.59395
20	H4	0.78760	0.25433	0.92981	68	O12	0.20411	0.28423	0.40470
21	H5	0.20294	0.84845	0.04539	69	O13	0.31112	0.23785	0.09595
22	H6	0.21806	0.85549	0.17227	70	O14	0.69207	0.73964	0.90401
23	H7	0.44310	0.83426	0.10162	71	O15	0.81581	0.74389	0.40156
24	H8	0.27576	0.71885	0.11355	72	O16	0.18760	0.24499	0.59766

25	H9	0.30619	0.84509	0.54847	73	O17	0.19747	0.54130	0.61726
26	H10	0.05756	0.82568	0.59840	74	O18	0.80510	0.04147	0.38358
27	H11	0.26775	0.85500	0.67488	75	O19	0.68971	0.04165	0.88438
28	H12	0.23506	0.71611	0.61673	76	O20	0.31503	0.54817	0.11791
29	H13	0.73012	0.35203	0.32170	77	O21	0.81747	0.48243	0.61433
30	H14	0.69356	0.34409	0.44823	78	O22	0.18629	0.98389	0.38331
31	H15	0.94159	0.32551	0.39827	79	O23	0.30730	0.99006	0.88503
32	H16	0.76739	0.21429	0.38153	80	O24	0.69702	0.48741	0.11339
33	H17	0.82904	0.05390	0.52000	81	O25	0.95422	0.63152	0.75243
34	H18	0.69740	0.11769	0.61952	82	O26	0.04369	0.13230	0.24553
35	H19	0.95586	0.14893	0.60243	83	O27	0.45844	0.13025	0.74297
36	H20	0.87229	0.00671	0.64284	84	O28	0.55346	0.62769	0.25726
37	H21	0.04482	0.64469	0.39716	85	O29	0.04667	0.40198	0.75927
38	H22	0.16941	0.55228	0.48307	86	O30	0.95434	0.90231	0.24126
39	H23	0.30563	0.61613	0.38579	87	O31	0.54241	0.89933	0.74537
40	H24	0.13755	0.50244	0.36030	88	O32	0.46028	0.39802	0.25283
41	H25	0.30555	0.60881	0.82030	89	F1	0.02463	0.14425	0.77558
42	H26	0.19006	0.61781	0.93736	90	F2	0.98193	0.64364	0.22396
43	H27	0.45552	0.62611	0.92082	91	F3	0.52614	0.64124	0.72151
44	H28	0.32249	0.48851	0.90226	92	F4	0.47563	0.14044	0.27604
45	H29	0.65991	0.04635	0.01732	93	F5	0.47263	0.39063	0.72987
46	H30	0.53659	0.14148	0.10180	94	F6	0.53417	0.89254	0.26729
47	H31	0.79830	0.11586	0.10914	95	F7	0.96434	0.89516	0.76703
48	H32	0.63423	0.00259	0.14353	96	F8	0.03874	0.39453	0.23233

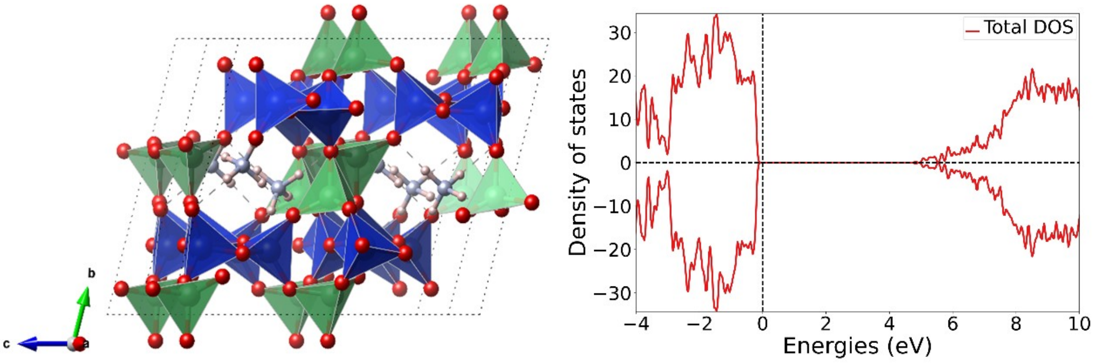

Figure S18. Structure diagram and density of states of $(NH_4)_2(Be_2Si_3O_9)$.

Table S24. Cell parameters of $(NH_4)_2(Be_2Si_3O_9)$, ICSD is the ICSD Collection Code, STRU is the chemical formula. The a, b, c (Å) and α, β, γ (°) are unit cell parameters. V ($Å^3$) is the cell volume and Structure parameters of $(NH_4)_2(Be_2Si_3O_9)$.

ICSD	STRU	a	b	c	α	β	γ	V
82398	$(NH_4)_2(Be_2Si_3O_9)$	7.03	10.50	12.23	105.11	90.00	90.00	871.74

NO.	E	x	y	z	NO.	E	x	y	z
1	Be1	0.08635	0.57474	0.96509	49	H29	0.29600	0.01026	0.85071
2	Be2	0.58633	0.42511	0.53498	50	H30	0.52926	0.00601	0.88938
3	Be3	0.91337	0.42506	0.03495	51	H31	0.44564	0.89569	0.77210
4	Be4	0.41345	0.57472	0.46500	52	H32	0.37027	0.89337	0.90875
5	Be5	0.06534	0.92851	0.05270	53	N1	0.56953	0.54141	0.84243
6	Be6	0.56529	0.07136	0.44725	54	N2	0.06963	0.45842	0.65767
7	Be7	0.93439	0.07134	0.94721	55	N3	0.42990	0.45853	0.15753
8	Be8	0.43446	0.92849	0.55280	56	N4	0.92980	0.54136	0.34248
9	Si1	0.81019	0.73263	0.12379	57	N5	0.08976	0.95006	0.35374
10	Si2	0.31011	0.26725	0.37623	58	N6	0.58946	0.05018	0.14590
11	Si3	0.18958	0.26722	0.87622	59	N7	0.91050	0.04993	0.64586
12	Si4	0.68964	0.73254	0.62377	60	N8	0.41041	0.94946	0.85398
13	Si5	0.61712	0.24311	0.91578	61	O1	0.92085	0.59324	0.06973
14	Si6	0.11722	0.75661	0.58418	62	O2	0.42096	0.40658	0.43024
15	Si7	0.38263	0.75671	0.08425	63	O3	0.07889	0.40660	0.93029
16	Si8	0.88256	0.24326	0.41582	64	O4	0.57881	0.59322	0.56973
17	Si9	0.47075	0.23274	0.67800	65	O5	0.97046	0.90591	0.92092
18	Si10	0.97075	0.76704	0.82201	66	O6	0.47044	0.09392	0.57905
19	Si11	0.52913	0.76713	0.32205	67	O7	0.02922	0.09391	0.07902
20	Si12	0.02899	0.23274	0.17798	68	O8	0.52934	0.90593	0.42102
21	H1	0.44885	0.57131	0.89241	69	O9	0.62666	0.22259	0.77700
22	H2	0.64092	0.62233	0.82962	70	O10	0.12665	0.77711	0.72297
23	H3	0.53863	0.47784	0.76258	71	O11	0.37321	0.77726	0.22304
24	H4	0.65313	0.49246	0.88901	72	O12	0.87308	0.22268	0.27702
25	H5	0.15315	0.50726	0.61096	73	O13	0.76080	0.73848	0.75567

26	H6	0.14093	0.37744	0.67040	74	O14	0.26081	0.26127	0.74434
27	H7	0.94876	0.42861	0.60782	75	O15	0.23893	0.26122	0.24432
28	H8	0.03901	0.52203	0.73754	76	O16	0.73909	0.73873	0.25572
29	H9	0.46066	0.52209	0.23740	77	O17	0.61381	0.74839	0.05341
30	H10	0.55074	0.42855	0.10771	78	O18	0.11374	0.25163	0.44665
31	H11	0.34664	0.50753	0.11080	79	O19	0.38594	0.25142	0.94661
32	H12	0.35831	0.37765	0.17023	80	O20	0.88601	0.74824	0.55337
33	H13	0.85855	0.62233	0.32968	81	O21	0.94235	0.86093	0.13280
34	H14	0.05058	0.57119	0.39240	82	O22	0.44210	0.13888	0.36718
35	H15	0.96057	0.47774	0.26265	83	O23	0.05737	0.13894	0.86714
36	H16	0.84619	0.49252	0.38915	84	O24	0.55758	0.86088	0.63284
37	H17	0.20430	0.01088	0.35070	85	O25	0.70720	0.38233	0.98465
38	H18	0.05415	0.89671	0.27176	86	O26	0.20724	0.61738	0.51527
39	H19	0.97106	0.00650	0.38950	87	O27	0.29255	0.61747	0.01541
40	H20	0.12989	0.89342	0.40803	88	O28	0.79253	0.38248	0.48470
41	H21	0.62970	0.10605	0.09095	89	O29	0.71011	0.11601	0.94926
42	H22	0.70368	0.98922	0.14917	90	O30	0.21025	0.88370	0.55072
43	H23	0.47038	0.99379	0.11068	91	O31	0.28958	0.88376	0.05073
44	H24	0.55457	0.10418	0.22773	92	O32	0.78953	0.11617	0.44929
45	H25	0.94558	0.10364	0.72778	93	O33	0.51726	0.36025	0.63275
46	H26	0.02949	0.99355	0.61042	94	O34	0.01733	0.63960	0.86733
47	H27	0.87024	0.10613	0.59121	95	O35	0.48266	0.63958	0.36725
48	H28	0.79621	0.98896	0.64898	96	O36	0.98250	0.36024	0.13272

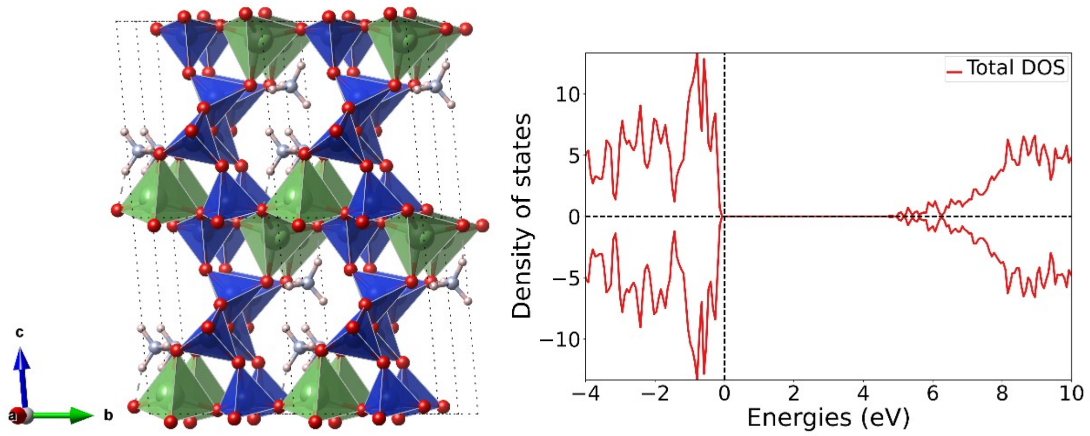

Figure S19. Structure diagram and density of states of $NH_4Li(Si_2O_5)$.

Table S25. Cell parameters of $NH_4Li(Si_2O_5)$, ICSD is the ICSD Collection Code, STRU is the chemical formula. The a, b, c (Å) and α, β, γ (°) are unit cell parameters. V (Å^3) is the cell volume and Structure parameters of $NH_4Li(Si_2O_5)$.

ICSD	STRU	a	b	c	α	β	γ	V
82457	$NH_4Li(Si_2O_5)$	4.82	6.30	8.45	94.93	89.99	90.03	255.75

NO.	E	x	y	z	NO.	E	x	y	z
1	Li1	0.77635	0.12186	0.09068	14	H8	0.87577	0.10042	0.77343
2	Li2	0.27668	0.87720	0.90775	15	N1	0.33145	0.98911	0.33823
3	Si1	0.87991	0.44651	0.39070	16	N2	0.83025	0.01133	0.66690
4	Si2	0.37981	0.55250	0.60553	17	O1	0.70752	0.51471	0.55571
5	Si3	0.74136	0.62449	0.07097	18	O2	0.20758	0.48353	0.44079
6	Si4	0.24139	0.37552	0.92589	19	O3	0.82754	0.20253	0.32371
7	H1	0.14159	0.07177	0.32893	20	O4	0.32712	0.79680	0.67212
8	H2	0.50366	0.09386	0.34759	21	O5	0.80751	0.61983	0.26190
9	H3	0.37110	0.89146	0.23556	22	O6	0.30733	0.37980	0.73489
10	H4	0.32552	0.90131	0.43591	23	O7	0.59960	0.84517	0.03734
11	H5	0.82352	0.10795	0.57466	24	O8	0.09880	0.15470	0.95929
12	H6	0.00019	0.90574	0.65110	25	O9	0.54391	0.41105	0.02033
13	H7	0.63969	0.92870	0.67339	26	O10	0.04408	0.58878	0.97654

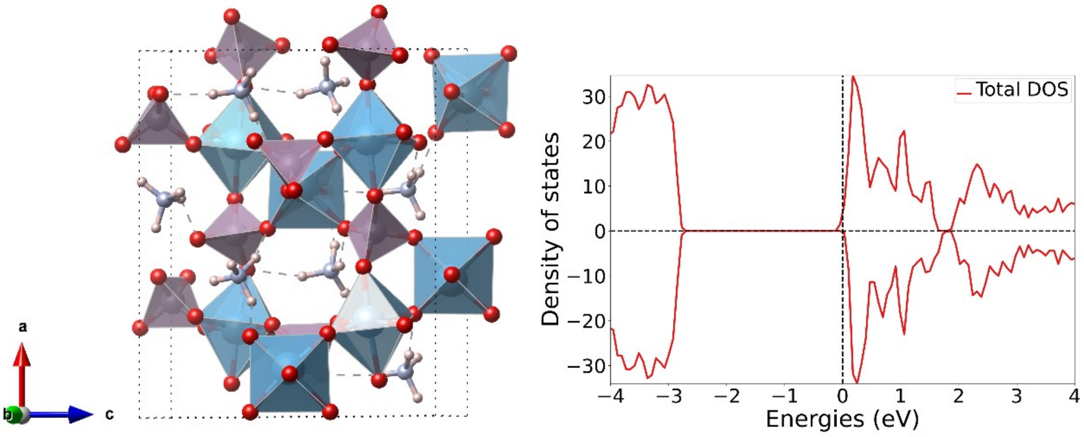

Figure S20. Structure diagram and density of states of $NH_4(Ti_{0.936}Sn_{0.064})O(PO_4)$.

Table S26. Cell parameters of $NH_4(Ti_{0.936}Sn_{0.064})O(PO_4)$, ICSD is the ICSD Collection Code, STRU is the chemical formula. The a, b, c (Å) and α, β, γ (°) are unit cell parameters. V ($Å^3$) is the cell volume and Structure parameters of $NH_4(Ti_{0.936}Sn_{0.064})O(PO_4)$.

ICSD	STRU	a	b	c	α	β	γ	V
91534	$NH_4(Ti_{0.936}Sn_{0.064})O(PO_4)$	13.02	6.56	10.69	89.91	89.56	89.83	913.43

NO.	E	x	y	z	NO.	E	x	y	z
1	N1	0.59392	0.82811	0.06769	53	O21	0.61624	0.80518	0.48394
2	N2	0.39850	0.18572	0.56030	54	O22	0.38524	0.19341	0.97822
3	N3	0.89906	0.31129	0.56097	55	O23	0.88348	0.30652	0.98050
4	N4	0.10004	0.68286	0.05771	56	O24	0.11561	0.69487	0.48305
5	N5	0.88942	0.70126	0.31464	57	O25	0.75523	0.95182	0.62364
6	N6	0.12072	0.28310	0.82117	58	O26	0.24693	0.04883	0.12274
7	N7	0.61815	0.21718	0.82333	59	O27	0.74554	0.45308	0.12544
8	N8	0.38500	0.79684	0.31519	60	O28	0.25486	0.54686	0.62205
9	Ti1	0.86866	0.00119	0.99776	61	O29	0.75137	0.03932	0.39394
10	Ti2	0.13087	0.99510	0.50324	62	O30	0.24724	0.96212	0.89261
11	Ti3	0.62895	0.50187	0.49992	63	O31	0.74532	0.53974	0.89524
12	Ti4	0.36836	0.49436	0.99943	64	O32	0.25027	0.45998	0.39209
13	Ti5	0.74619	0.23247	0.25092	65	O33	0.72140	0.44888	0.38638
14	Ti6	0.25806	0.76101	0.74935	66	O34	0.27811	0.55155	0.88005
15	Ti7	0.75363	0.73226	0.74757	67	O35	0.77252	0.94800	0.88898
16	Ti8	0.24238	0.26319	0.25218	68	O36	0.21891	0.05361	0.38123
17	Sn1	0.86866	0.00119	0.99776	69	O37	0.72474	0.53878	0.63643
18	Sn2	0.13087	0.99510	0.50324	70	O38	0.27591	0.45996	0.13226
19	Sn3	0.62895	0.50187	0.49992	71	O39	0.77850	0.04379	0.13837
20	Sn4	0.36836	0.49436	0.99943	72	O40	0.22533	0.95811	0.63185
21	Sn5	0.74619	0.23247	0.25092	73	H1	0.51741	0.84599	0.04064
22	Sn6	0.25806	0.76101	0.74935	74	H2	0.59744	0.68383	0.10659

23	Sn7	0.75363	0.73226	0.74757	75	H3	0.61330	0.93904	0.13140
24	Sn8	0.24238	0.26319	0.25218	76	H4	0.64317	0.83522	0.99189
25	P1	-0.00059	0.15465	0.25685	77	H5	0.33772	0.10305	0.59908
26	P2	-0.00008	0.84099	0.75706	78	H6	0.46708	0.10755	0.56486
27	P3	0.50169	0.65882	0.75727	79	H7	0.41127	0.32407	0.60470
28	P4	0.49965	0.34175	0.25601	80	H8	0.38366	0.21591	0.46730
29	P5	0.68331	0.99526	0.51044	81	H9	0.84022	0.39585	0.60226
30	P6	0.31667	0.00515	0.00726	82	H10	0.91231	0.17221	0.60495
31	P7	0.81542	0.49583	0.00934	83	H11	0.96872	0.38569	0.56199
32	P8	0.18287	0.50407	0.50932	84	H12	0.88089	0.28133	0.46904
33	O1	0.98666	0.01592	0.14245	85	H13	0.03241	0.60321	0.06629
34	O2	0.01219	0.98293	0.64316	86	H14	0.16193	0.60342	0.09588
35	O3	0.51285	0.51448	0.64429	87	H15	0.08651	0.82294	0.10024
36	O4	0.48682	0.48093	0.14198	88	H16	0.11287	0.70874	0.96324
37	O5	0.01322	0.02645	0.37813	89	H17	0.81814	0.69978	0.35644
38	O6	0.98190	0.97038	0.87795	90	H18	0.91591	0.55171	0.31294
39	O7	0.48564	0.53603	0.88001	91	H19	0.93853	0.80431	0.35787
40	O8	0.51431	0.47194	0.37722	92	H20	0.88049	0.74499	0.22245
41	O9	0.90117	0.28925	0.27891	93	H21	0.04970	0.28479	0.86343
42	O10	0.09843	0.71238	0.77925	94	H22	0.15936	0.15011	0.84674
43	O11	0.60038	0.78911	0.77365	95	H23	0.16664	0.40499	0.84738
44	O12	0.40265	0.20759	0.27903	96	H24	0.11251	0.28713	0.72487
45	O13	0.09287	0.30017	0.23712	97	H25	0.54689	0.21349	0.86506
46	O14	0.90421	0.70084	0.73864	98	H26	0.66451	0.09470	0.84855
47	O15	0.40661	0.80220	0.73645	99	H27	0.65561	0.34964	0.85070
48	O16	0.59516	0.19865	0.23823	100	H28	0.61024	0.21672	0.72705
49	O17	0.61577	0.18400	0.54294	101	H29	0.31509	0.81220	0.35976
50	O18	0.38371	0.81490	0.03582	102	H30	0.43344	0.69502	0.36058
51	O19	0.88214	0.68568	0.03822	103	H31	0.41448	0.94355	0.30597
52	O20	0.11542	0.31628	0.54264	104	H32	0.37203	0.74248	0.22626

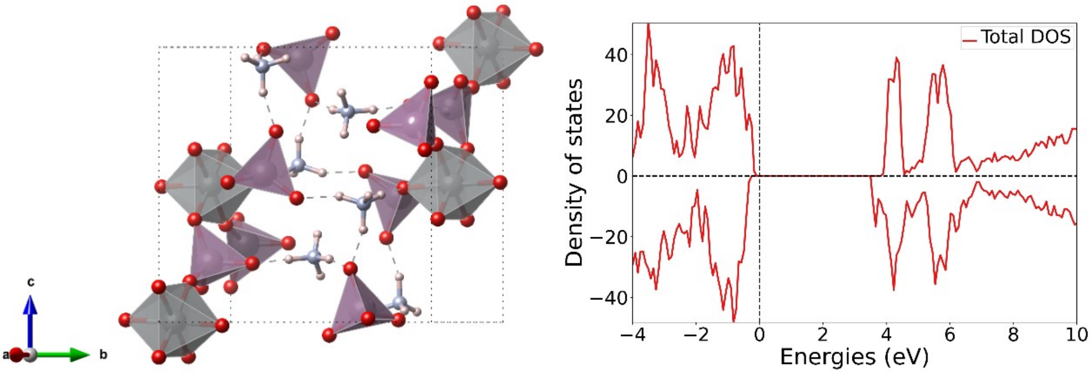

Figure S21. Structure diagram and density of states of $(NH_4)_2Ni(MoO_4)_2$.

Table S27. Cell parameters of $(NH_4)_2Ni(MoO_4)_2$, ICSD is the ICSD Collection Code, STRU is the chemical formula. The a, b, c (Å) and α, β, γ (°) are unit cell parameters. V (Å^3) is the cell volume and Structure parameters of $(NH_4)_2Ni(MoO_4)_2$.

ICSD	STRU	a	b	c	α	β	γ	V
200310	$(NH_4)_2Ni(MoO_4)_2$	8.49	11.01	11.10	90.00	90.00	113.02	955.38

NO.	E	x	y	z	NO.	E	x	y	z
1	Ni1	0.32546	0.01119	0.49938	43	H31	0.68901	0.38612	0.95730
2	Ni2	0.82546	0.01119	0.00062	44	H32	0.65659	0.29192	0.83517
3	Ni3	0.67457	0.98897	0.50069	45	N1	0.02700	0.56866	0.73548
4	Ni4	0.17457	0.98897	0.99931	46	N2	0.47486	0.43163	0.23651
5	Mo1	0.47384	0.93781	0.78364	47	N3	0.97486	0.43163	0.26349
6	Mo2	0.52603	0.06215	0.21623	48	N4	0.52700	0.56866	0.76452
7	Mo3	0.02603	0.06215	0.28377	49	N5	0.14901	0.28781	0.57034
8	Mo4	0.97384	0.93781	0.71636	50	N6	0.85014	0.71226	0.43069
9	Mo5	0.17226	0.29796	0.95889	51	N7	0.35014	0.71226	0.06931
10	Mo6	0.82807	0.70254	0.04173	52	N8	0.64901	0.28781	0.92966
11	Mo7	0.32807	0.70254	0.45827	53	O1	0.49723	0.99949	0.62786
12	Mo8	0.67226	0.29796	0.54111	54	O2	0.50311	0.00061	0.37206
13	H1	0.91366	0.48439	0.72830	55	O3	0.00311	0.00061	0.12794
14	H2	0.08494	0.57082	0.81652	56	O4	0.99723	0.99949	0.87214
15	H3	0.99593	0.65274	0.72838	57	O5	0.41746	0.76676	0.78676
16	H4	0.11189	0.56831	0.66688	58	O6	0.58314	0.23328	0.21303
17	H5	0.38888	0.43195	0.16875	59	O7	0.08314	0.23328	0.28697
18	H6	0.50625	0.34773	0.22844	60	O8	0.91746	0.76676	0.71324
19	H7	0.41824	0.42877	0.31822	61	O9	0.65522	0.01767	0.87441
20	H8	0.58780	0.51618	0.22904	62	O10	0.69336	0.03280	0.14644
21	H9	0.88888	0.43195	0.33125	63	O11	0.19336	0.03280	0.35356
22	H10	0.00625	0.34773	0.27156	64	O12	0.15522	0.01767	0.62559
23	H11	0.08780	0.51618	0.27096	65	O13	0.34452	0.98268	0.12538
24	H12	0.91824	0.42877	0.18178	66	O14	0.30625	0.96685	0.85350
25	H13	0.41366	0.48439	0.77170	67	O15	0.80625	0.96685	0.64650

26	H14	0.58494	0.57082	0.68348	68	O16	0.84452	0.98268	0.37462
27	H15	0.61189	0.56831	0.83312	69	O17	0.25793	0.44206	0.04936
28	H16	0.49593	0.65274	0.77162	70	O18	0.74233	0.55770	0.95221
29	H17	0.02348	0.23691	0.54245	71	O19	0.24233	0.55770	0.54779
30	H18	0.23053	0.24551	0.53883	72	O20	0.75793	0.44206	0.45064
31	H19	0.15659	0.29192	0.66483	73	O21	0.21034	0.35387	0.80740
32	H20	0.18901	0.38612	0.54270	74	O22	0.79026	0.64788	0.19353
33	H21	0.76809	0.75403	0.46242	75	O23	0.29026	0.64788	0.30647
34	H22	0.81041	0.61371	0.45749	76	O24	0.71034	0.35387	0.69260
35	H23	0.97545	0.76310	0.45897	77	O25	0.94884	0.21888	0.98757
36	H24	0.84313	0.70900	0.33616	78	O26	0.71281	0.80332	0.00772
37	H25	0.26809	0.75403	0.03758	79	O27	0.21281	0.80332	0.49228
38	H26	0.31041	0.61371	0.04251	80	O28	0.44884	0.21888	0.51243
39	H27	0.34313	0.70900	0.16384	81	O29	0.05144	0.78164	0.01260
40	H28	0.47545	0.76310	0.04103	82	O30	0.28705	0.19655	0.99189
41	H29	0.52348	0.23691	0.95755	83	O31	0.78705	0.19655	0.50811
42	H30	0.73053	0.24551	0.96117	84	O32	0.55144	0.78164	0.48740

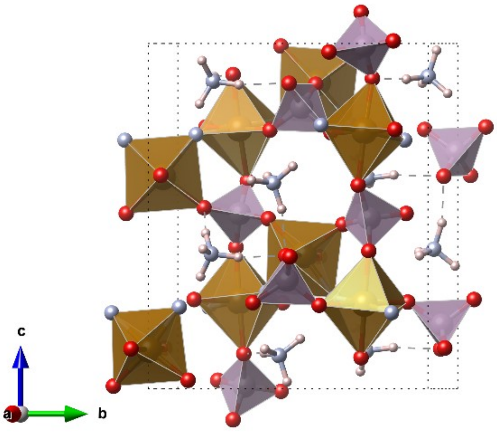

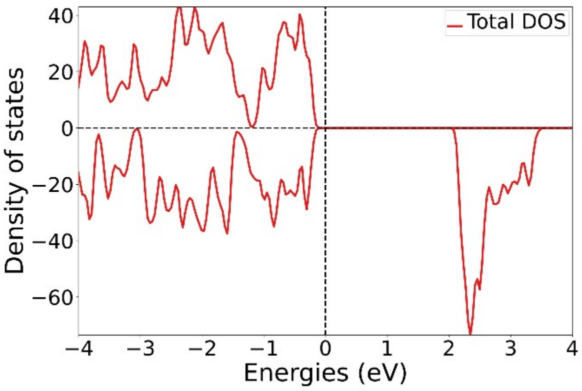

Figure S22. Structure diagram and density of states of $NH_4Fe(PO_4)F$.

Table S28. Cell parameters of $NH_4Fe(PO_4)F$, ICSD is the ICSD Collection Code, STRU is the chemical formula. The a, b, c (Å) and α, β, γ (°) are unit cell parameters. V (Å^3) is the cell volume and Structure parameters of $NH_4Fe(PO_4)F$.

ICSD	STRU	a	b	c	α	β	γ	V
200613	$NH_4Fe(PO_4)F$	6.52	10.81	13.13	90.00	90.00	90.00	925.15

NO.	E	x	y	z	NO.	E	x	y	z
1	Fe1	0.51605	0.00218	0.61345	49	N1	0.84558	0.43157	0.59594
2	Fe2	0.48375	0.50218	0.38667	50	N2	0.15402	0.93166	0.40428
3	Fe3	0.01625	0.50218	0.88667	51	N3	0.34598	0.93166	0.90428
4	Fe4	0.98395	0.00218	0.11345	52	N4	0.65442	0.43157	0.09594
5	Fe5	0.25087	0.24666	0.75355	53	N5	0.21798	0.70306	0.60950
6	Fe6	0.74916	0.74666	0.24656	54	N6	0.78147	0.20303	0.39055
7	Fe7	0.75084	0.74666	0.74656	55	N7	0.71853	0.20303	0.89055
8	Fe8	0.24913	0.24666	0.25355	56	N8	0.28202	0.70306	0.10950
9	P1	0.00519	0.99650	0.68546	57	O1	0.79832	0.72956	0.59012
10	P2	0.99460	0.49645	0.31464	58	O2	0.20155	0.22949	0.40997
11	P3	0.50540	0.49645	0.81464	59	O3	0.29845	0.22949	0.90997
12	P4	0.49481	0.99650	0.18546	60	O4	0.70168	0.72956	0.09012
13	P5	0.33062	0.25548	0.50701	61	O5	0.55036	0.60625	0.74173
14	P6	0.66913	0.75547	0.49311	62	O6	0.44993	0.10632	0.25837
15	P7	0.83087	0.75547	0.99311	63	O7	0.05007	0.10632	0.75837
16	P8	0.16938	0.25548	0.00701	64	O8	0.94964	0.60625	0.24173
17	H1	0.96192	0.36585	0.60360	65	O9	0.96442	0.87883	0.75092
18	H2	0.83953	0.46070	0.52010	66	O10	0.03542	0.37879	0.24917
19	H3	0.87483	0.50419	0.64435	67	O11	0.46458	0.37879	0.74916
20	H4	0.70403	0.39561	0.61484	68	O12	0.53558	0.87883	0.25092
21	H5	0.03777	0.86587	0.39662	69	O13	0.81672	0.02655	0.61801
22	H6	0.12489	0.00413	0.35571	70	O14	0.18310	0.52654	0.38206
23	H7	0.29569	0.89568	0.38562	71	O15	0.31690	0.52654	0.88206
24	H8	0.15979	0.96094	0.48008	72	O16	0.68328	0.02655	0.11801
25	H9	0.46223	0.86587	0.89662	73	O17	0.18966	0.26884	0.60147

26	H10	0.37511	0.00413	0.85571	74	O18	0.81011	0.76880	0.39865
27	H11	0.34021	0.96094	0.98008	75	O19	0.68989	0.76880	0.89865
28	H12	0.20431	0.89568	0.88562	76	O20	0.31034	0.26884	0.10147
29	H13	0.53808	0.36585	0.10360	77	O21	0.48086	0.14504	0.52452
30	H14	0.66047	0.46070	0.02010	78	O22	0.51898	0.64498	0.47569
31	H15	0.79597	0.39561	0.11484	79	O23	0.98102	0.64498	0.97569
32	H16	0.62517	0.50419	0.14435	80	O24	0.01914	0.14504	0.02452
33	H17	0.26448	0.65919	0.67495	81	O25	0.19606	0.97154	0.61543
34	H18	0.31176	0.67257	0.55000	82	O26	0.80377	0.47145	0.38470
35	H19	0.23792	0.79744	0.61926	83	O27	0.69623	0.47145	0.88470
36	H20	0.06263	0.69090	0.59513	84	O28	0.30394	0.97154	0.11543
37	H21	0.68826	0.17277	0.45033	85	O29	0.46441	0.37281	0.49246
38	H22	0.73345	0.15930	0.32528	86	O30	0.53529	0.87279	0.50763
39	H23	0.93683	0.19042	0.40444	87	O31	0.96471	0.87279	0.00763
40	H24	0.76213	0.29749	0.38089	88	O32	0.03559	0.37281	0.99246
41	H25	0.81174	0.17277	0.95033	89	F1	0.52893	0.86730	0.72000
42	H26	0.76655	0.15930	0.82528	90	F2	0.47099	0.36730	0.28011
43	H27	0.73787	0.29749	0.88089	91	F3	0.02901	0.36730	0.78011
44	H28	0.56317	0.19042	0.90444	92	F4	0.97107	0.86730	0.22000
45	H29	0.23552	0.65919	0.17495	93	F5	0.46778	0.11516	0.73622
46	H30	0.18824	0.67257	0.05000	94	F6	0.53203	0.61526	0.26398
47	H31	0.43737	0.69090	0.09513	95	F7	0.96797	0.61526	0.76398
48	H32	0.26208	0.79744	0.11926	96	F8	0.03222	0.11516	0.23622

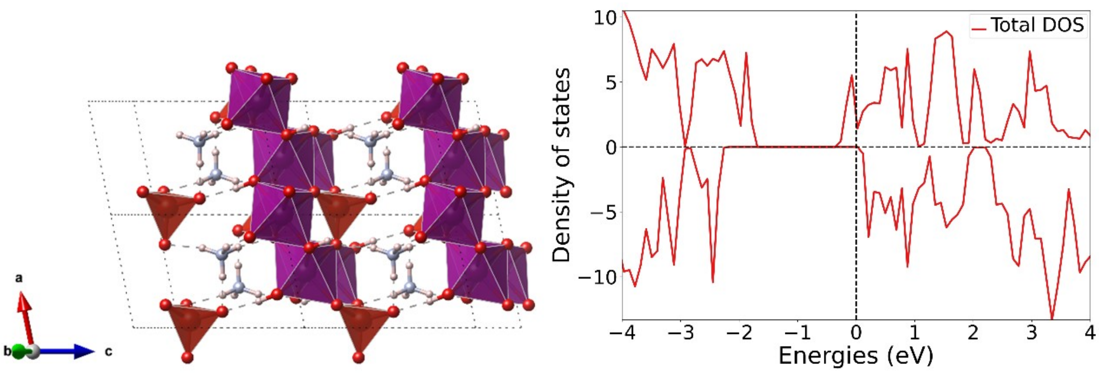

Figure S23. Structure diagram and density of states of $(NH_4)_2(Mn_3(OH)_2(VO_4)_2)$.

Table S29. Cell parameters of $(NH_4)_2(Mn_3(OH)_2(VO_4)_2)$, ICSD is the ICSD Collection Code, STRU is the chemical formula. The a, b, c (Å) and α, β, γ (°) are unit cell parameters. V (Å^3) is the cell volume and Structure parameters of $(NH_4)_2(Mn_3(OH)_2(VO_4)_2)$.

ICSD	STRU	a	b	c	α	β	γ	V
203214	$(NH_4)_2(Mn_3(OH)_2(VO_4)_2)$	5.54	6.37	8.21	112.86	101.09	90.00	261.09

NO.	E	x	y	z	NO.	E	x	y	z
1	N1	0.64562	0.77014	0.54138	15	H8	0.54857	0.24144	0.48333
2	N2	0.35428	0.22914	0.45842	16	H9	0.76626	0.13603	0.27194
3	Mn1	0.99997	0.99994	0.99990	17	H10	0.23367	0.86398	0.72786
4	Mn2	0.49998	0.73637	0.99989	18	O1	0.70518	0.07015	0.14009
5	Mn3	0.49996	0.26354	0.99990	19	O2	0.29475	0.92977	0.85970
6	V1	0.93495	0.38088	0.76237	20	O3	0.79948	0.18973	0.83565
7	V2	0.06499	0.61893	0.23744	21	O4	0.79955	0.64545	0.83562
8	H1	0.71210	0.68044	0.62087	22	O5	0.20043	0.81004	0.16410
9	H2	0.71211	0.93944	0.62080	23	O6	0.20044	0.35439	0.16425
10	H3	0.45132	0.75778	0.51646	24	O7	0.85855	0.26571	0.53241
11	H4	0.70365	0.70918	0.41956	25	O8	0.14138	0.73420	0.46740
12	H5	0.29624	0.28996	0.58022	26	O9	0.26407	0.41438	0.82918
13	H6	0.28790	0.31905	0.37909	27	O10	0.73588	0.58546	0.17062
14	H7	0.28769	0.05987	0.37881					

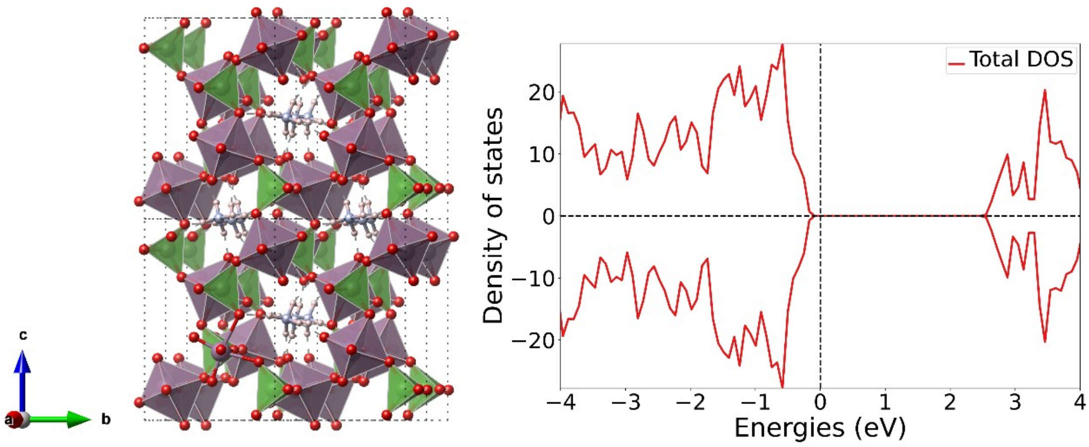

Figure S24. Structure diagram and density of states of $NH_4(MoO_2)(AsO_4)$.

Table S30. Cell parameters of $NH_4(MoO_2)(AsO_4)$, ICSD is the ICSD Collection Code, STRU is the chemical formula. The a, b, c (Å) and α, β, γ (°) are unit cell parameters. V (Å^3) is the cell volume and Structure parameters of $NH_4(MoO_2)(AsO_4)$.

ICSD	STRU	a	b	c	α	β	γ	V
203218	$NH_4(MoO_2)(AsO_4)$	6.72	7.29	11.07	90.00	90.00	90.00	542.13

NO.	E	x	y	z	NO.	E	x	y	z
1	Mo1	0.24723	0.45595	0.65966	27	N3	0.50462	0.00882	0.50351
2	Mo2	0.24723	0.04405	0.15966	28	N4	0.00465	0.50878	0.99667
3	Mo3	0.74725	0.54409	0.34047	29	O1	0.95296	0.40682	0.65246
4	Mo4	0.74726	0.95591	0.84047	30	O2	0.54112	0.09231	0.15015
5	As1	0.74737	0.55095	0.65333	31	O3	0.45299	0.59316	0.34766
6	As2	0.74737	0.94905	0.15333	32	O4	0.04115	0.90765	0.84994
7	As3	0.24737	0.44908	0.34679	33	O5	0.04115	0.59236	0.34994
8	As4	0.24738	0.05093	0.84679	34	O6	0.45300	0.90683	0.84766
9	H1	0.92523	0.95617	0.42007	35	O7	0.54112	0.40769	0.65015
10	H2	0.91000	0.01296	0.56958	36	O8	0.95295	0.09318	0.15247
11	H3	0.08814	0.10940	0.48120	37	O9	0.25051	0.16368	0.71041
12	H4	0.09420	0.88041	0.51716	38	O10	0.25050	0.33632	0.21041
13	H5	0.40999	0.51292	0.93058	39	O11	0.75051	0.83634	0.28973
14	H6	0.42519	0.45615	0.08010	40	O12	0.75052	0.66365	0.78973
15	H7	0.58813	0.60936	0.01897	41	O13	0.24438	0.31513	0.47446
16	H8	0.59418	0.38037	0.98303	42	O14	0.24439	0.18487	0.97446
17	H9	0.40999	0.98708	0.43058	43	O15	0.74442	0.68492	0.52567
18	H10	0.42519	0.04386	0.58010	44	O16	0.74442	0.81508	0.02567
19	H11	0.59418	0.11963	0.48303	45	O17	0.24268	0.66356	0.58765
20	H12	0.58814	0.89064	0.51897	46	O18	0.24267	0.83644	0.08765
21	H13	0.92523	0.54383	0.92007	47	O19	0.74275	0.33649	0.41249
22	H14	0.91000	0.48704	0.06958	48	O20	0.74275	0.16351	0.91249
23	H15	0.09420	0.61959	0.01716	49	O21	0.25299	0.52232	0.80715
24	H16	0.08814	0.39060	0.98120	50	O22	0.25299	0.97768	0.30715

25	N1	0.00465	0.99122	0.49667	51	O23	0.75299	0.47771	0.19297
26	N2	0.50462	0.49118	0.00351	52	O24	0.75299	0.02229	0.69297

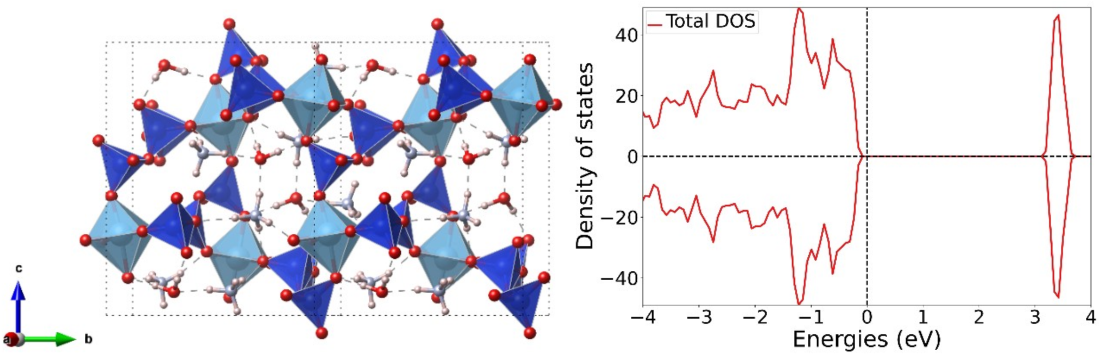

Figure S25. Structure diagram and density of states of $(NH_4)_2(TiSi_3O_9)(H_2O)$.

Table S31. Cell parameters of $(NH_4)_2(TiSi_3O_9)(H_2O)$, ICSD is the ICSD Collection Code, STRU is the chemical formula. The a, b, c (Å) and α, β, γ (°) are unit cell parameters. V ($Å^3$) is the cell volume and Structure parameters of $(NH_4)_2(TiSi_3O_9)(H_2O)$.

ICSD	STRU	a	b	c	α	β	γ	V
280327	$(NH_4)_2(TiSi_3O_9)(H_2O)$	7.34	10.20	13.18	90.00	90.00	90.00	985.68

NO.	E	x	y	z	NO.	E	x	y	z
1	Ti1	0.74258	0.96301	0.79217	53	H37	0.54408	0.66621	0.61780
2	Ti2	0.73581	0.03638	0.29660	54	H38	0.94383	0.33374	0.12911
3	Ti3	0.24253	0.53715	0.20783	55	H39	0.04406	0.83396	0.38222
4	Ti4	0.23585	0.46378	0.70339	56	H40	0.44389	0.16641	0.87088
5	Si1	0.73503	0.95483	0.05133	57	N1	0.75434	0.43505	0.59293
6	Si2	0.75093	0.04395	0.55231	58	N2	0.72618	0.56566	0.08908
7	Si3	0.23504	0.54532	0.94866	59	N3	0.25429	0.06511	0.40705
8	Si4	0.25087	0.45624	0.44770	60	N4	0.22626	0.93448	0.91091
9	Si5	0.52383	0.67390	0.82284	61	N5	0.87882	0.31393	0.88241
10	Si6	0.96445	0.32380	0.32456	62	N6	0.68499	0.67747	0.36470
11	Si7	0.02382	0.82624	0.17716	63	N7	0.37876	0.18620	0.11760
12	Si8	0.46453	0.17639	0.67545	64	N8	0.18503	0.82268	0.63531
13	Si9	0.93541	0.65990	0.82449	65	O1	0.55002	0.83147	0.80862
14	Si10	0.55168	0.33993	0.32717	66	O2	0.93673	0.16720	0.30728
15	Si11	0.43541	0.84025	0.17551	67	O3	0.04998	0.66866	0.19134
16	Si12	0.05175	0.16023	0.67284	68	O4	0.43680	0.33298	0.69271
17	H1	0.63673	0.40304	0.63076	69	O5	0.72516	0.60314	0.84139
18	H2	0.87170	0.39903	0.62843	70	O6	0.76310	0.39626	0.34351
19	H3	0.75425	0.53723	0.59143	71	O7	0.22515	0.89699	0.15861
20	H4	0.74872	0.40023	0.51996	72	O8	0.26319	0.10393	0.65651
21	H5	0.61161	0.60260	0.12690	73	O9	0.92993	0.81755	0.80316
22	H6	0.73026	0.46351	0.09346	74	O10	0.56155	0.18155	0.31218
23	H7	0.84587	0.60226	0.12136	75	O11	0.42998	0.68260	0.19684
24	H8	0.72130	0.59378	0.01428	76	O12	0.06163	0.31861	0.68783
25	H9	0.13669	0.09712	0.36922	77	O13	0.90685	0.85375	0.07291

26	H10	0.25419	0.96293	0.40856	78	O14	0.58415	0.15065	0.57177
27	H11	0.24867	0.09993	0.48003	79	O15	0.40685	0.64642	0.92709
28	H12	0.37167	0.10111	0.37156	80	O16	0.08408	0.34954	0.42824
29	H13	0.22145	0.90632	0.98570	81	O17	0.94994	0.08681	0.76684
30	H14	0.34592	0.89788	0.87858	82	O18	0.53492	0.91942	0.26714
31	H15	0.23035	0.03663	0.90654	83	O19	0.44986	0.41331	0.23316
32	H16	0.11166	0.89756	0.87311	84	O20	0.03494	0.58075	0.73285
33	H17	0.77775	0.37140	0.85238	85	O21	0.73838	0.93009	0.63908
34	H18	0.88107	0.22219	0.84704	86	O22	0.73801	0.07101	0.13743
35	H19	0.86241	0.30495	0.96217	87	O23	0.23833	0.57010	0.36092
36	H20	0.00441	0.35844	0.86774	88	O24	0.23803	0.42913	0.86257
37	H21	0.56869	0.67800	0.31935	89	O25	0.54249	0.87202	0.06789
38	H22	0.74761	0.58733	0.35608	90	O26	0.94657	0.12305	0.56618
39	H23	0.65231	0.69197	0.44181	91	O27	0.04247	0.62811	0.93211
40	H24	0.76914	0.75238	0.33918	92	O28	0.44652	0.37716	0.43384
41	H25	0.27767	0.12873	0.14762	93	O29	0.56584	0.10517	0.77114
42	H26	0.50434	0.14168	0.13228	94	O30	0.91801	0.89051	0.27431
43	H27	0.38102	0.27794	0.15297	95	O31	0.06570	0.39509	0.22889
44	H28	0.36237	0.19519	0.03784	96	O32	0.41802	0.60961	0.72570
45	H29	0.06874	0.82215	0.68067	97	O33	0.74862	0.00378	0.93450
46	H30	0.26919	0.74777	0.66082	98	O34	0.73768	0.99200	0.43624
47	H31	0.24766	0.91282	0.64393	99	O35	0.24864	0.49637	0.06549
48	H32	0.15234	0.80818	0.55821	100	O36	0.23763	0.50824	0.56376
49	H33	0.68202	0.78545	0.59886	101	O37	0.64295	0.69667	0.57196
50	H34	0.80617	0.21248	0.10784	102	O38	0.84068	0.30456	0.08497
51	H35	0.18198	0.71472	0.40114	103	O39	0.14293	0.80349	0.42806
52	H36	0.30620	0.28765	0.89214	104	O40	0.34071	0.19557	0.91501

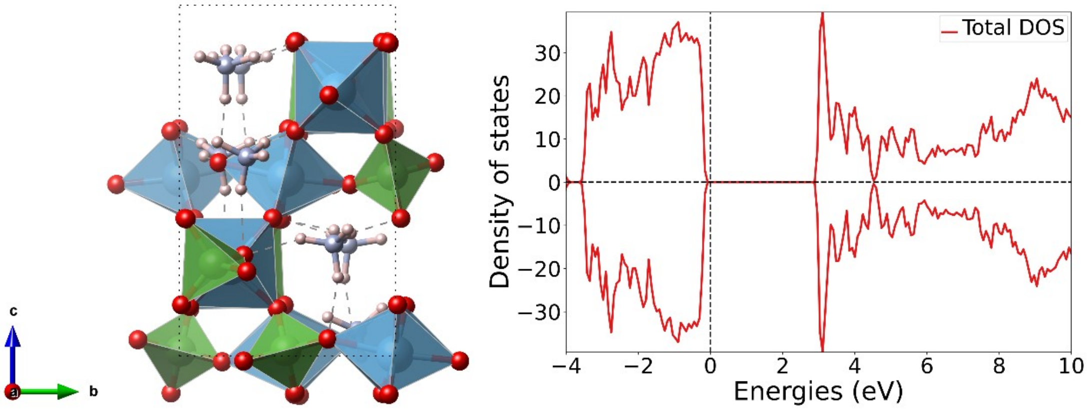

Figure S26. Structure diagram and density of states of $(NH_4)_2(TiO)_2(As_{0.43}P_{0.57}O_4)_2$.

Table S32. Cell parameters of $(NH_4)_2(TiO)_2(As_{0.43}P_{0.57}O_4)_2$, ICSD is the ICSD Collection Code, STRU is the chemical formula. The a, b, c (Å) and α, β, γ (°) are unit cell parameters. V (Å^3) is the cell volume and Structure parameters of $(NH_4)_2(TiO)_2(As_{0.43}P_{0.57}O_4)_2$.

ICSD	STRU	a	b	c	α	β	γ	V
400850	$(NH_4)_2(TiO)_2(As_{.43}P_{.57}O_4)_2$	13.22	6.69	10.85	89.96	89.70	89.70	959.36

NO.	E	x	y	z	NO.	E	x	y	z
1	N1	0.88805	0.71508	0.31900	53	O21	0.61080	0.79061	0.48881
2	N2	0.12294	0.28401	0.82803	54	O22	0.38841	0.19875	0.98410
3	N3	0.62292	0.21576	0.82775	55	O23	0.88690	0.29755	0.98607
4	N4	0.38591	0.78199	0.31712	56	O24	0.10990	0.70935	0.48900
5	N5	0.59856	0.82594	0.07277	57	O25	0.75906	0.94698	0.63175
6	N6	0.39565	0.18683	0.56517	58	O26	0.24193	0.04726	0.13370
7	N7	0.89551	0.31244	0.56687	59	O27	0.73944	0.45316	0.13477
8	N8	0.10217	0.68753	0.06105	60	O28	0.25877	0.55100	0.63049
9	Ti1	0.86781	0.99796	0.00557	61	O29	0.75176	0.02529	0.39240
10	Ti2	0.12943	0.00747	0.50737	62	O30	0.24400	0.96181	0.89533
11	Ti3	0.62893	0.49267	0.50753	63	O31	0.74330	0.53686	0.89612
12	Ti4	0.36940	0.49622	0.00309	64	O32	0.25078	0.47360	0.39083
13	Ti5	0.74314	0.23142	0.25610	65	O33	0.77880	0.04690	0.14202
14	Ti6	0.25652	0.76708	0.75198	66	O34	0.22387	0.96048	0.64033
15	Ti7	0.75446	0.72988	0.75310	67	O35	0.72351	0.53698	0.64159
16	Ti8	0.24113	0.26904	0.25409	68	O36	0.27498	0.45504	0.13774
17	As1	0.68152	0.98637	0.51547	69	O37	0.71875	0.43178	0.39279
18	As2	0.31668	0.00522	0.01460	70	O38	0.27887	0.55588	0.88830
19	As3	0.81544	0.49269	0.01614	71	O39	0.77406	0.93984	0.89412
20	As4	0.18099	0.51259	0.51443	72	O40	0.21800	0.06792	0.39118
21	As5	0.99728	0.16049	0.26277	73	H1	0.82500	0.79393	0.34980
22	As6	0.00062	0.84197	0.76295	74	H2	0.88275	0.56284	0.33413
23	As7	0.50194	0.65850	0.76227	75	H3	0.95055	0.77918	0.36017

24	As8	0.49672	0.33614	0.26217	76	H4	0.89325	0.73442	0.22430
25	P1	0.68152	0.98637	0.51547	77	H5	0.05382	0.28191	0.87172
26	P2	0.31668	0.00522	0.01460	78	H6	0.16261	0.15384	0.85139
27	P3	0.81544	0.49269	0.01614	79	H7	0.16729	0.40521	0.85330
28	P4	0.18099	0.51259	0.51443	80	H8	0.11250	0.28870	0.73350
29	P5	0.99728	0.16049	0.26277	81	H9	0.55377	0.21481	0.87129
30	P6	0.00062	0.84197	0.76295	82	H10	0.66873	0.09516	0.85266
31	P7	0.50194	0.65850	0.76227	83	H11	0.66092	0.34636	0.85212
32	P8	0.49672	0.33614	0.26217	84	H12	0.61257	0.21232	0.73321
33	O1	0.98617	0.01012	0.14638	85	H13	0.32277	0.70407	0.34737
34	O2	0.01194	0.99556	0.64750	86	H14	0.44822	0.71574	0.35843
35	O3	0.51205	0.50370	0.64722	87	H15	0.38041	0.93407	0.33289
36	O4	0.48437	0.48476	0.14520	88	H16	0.39157	0.76325	0.22243
37	O5	0.01252	0.03169	0.39091	89	H17	0.52251	0.82853	0.04743
38	O6	0.98424	0.97013	0.89115	90	H18	0.61210	0.68695	0.11087
39	O7	0.48706	0.53246	0.89154	91	H19	0.61200	0.93873	0.13565
40	O8	0.51301	0.46728	0.38926	92	H20	0.64561	0.84390	0.99730
41	O9	0.89453	0.29760	0.28729	93	H21	0.33794	0.10310	0.60542
42	O10	0.10398	0.70722	0.78409	94	H22	0.46499	0.11566	0.56943
43	O11	0.60378	0.79543	0.77917	95	H23	0.40638	0.32440	0.60744
44	O12	0.39398	0.20166	0.28746	96	H24	0.37911	0.21228	0.47343
45	O13	0.09417	0.31187	0.24087	97	H25	0.83637	0.39326	0.60731
46	O14	0.90291	0.69198	0.74310	98	H26	0.90988	0.17558	0.60929
47	O15	0.40315	0.80828	0.74181	99	H27	0.96306	0.38845	0.57016
48	O16	0.59451	0.18332	0.24130	100	H28	0.87886	0.28523	0.47552
49	O17	0.61172	0.18246	0.55125	101	H29	0.03206	0.62075	0.06645
50	O18	0.38669	0.80789	0.04684	102	H30	0.15906	0.60138	0.10116
51	O19	0.88547	0.68814	0.04905	103	H31	0.09315	0.82610	0.10289
52	O20	0.11190	0.31722	0.55102	104	H32	0.11863	0.71109	0.96877

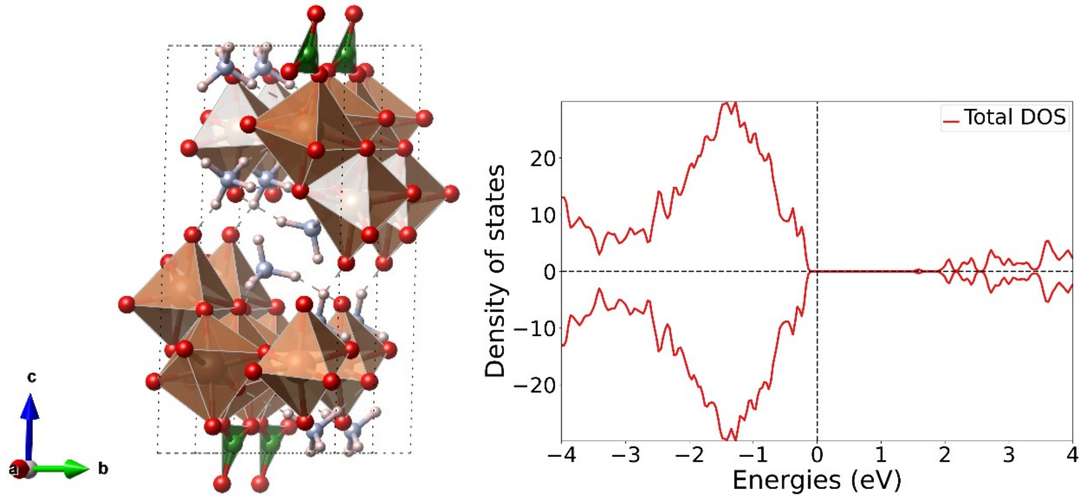

Figure S27. Structure diagram and density of states of $(NH_4)_3(BSb_4O_{13})$.

Table S33. Cell parameters of $(NH_4)_3(BSb_4O_{13})$, ICSD is the ICSD Collection Code, STRU is the chemical formula. The a, b, c (Å) and α, β, γ (°) are unit cell parameters. V ($Å^3$) is the cell volume and Structure parameters of $(NH_4)_3(BSb_4O_{13})$.

ICSD	STRU	a	b	c	α	β	γ	V
411501	$(NH_4)_3(BSb_4O_{13})$	7.24	7.38	13.70	83.06	80.94	62.83	642.27

NO.	E	x	y	z	NO.	E	x	y	z
1	B1	0.16135	0.24573	0.02283	34	H24	0.42171	0.61006	0.09562
2	B2	0.83949	0.74950	0.97858	35	N1	0.66454	0.29009	0.67143
3	Sb1	0.41319	0.95272	0.60756	36	N2	0.32549	0.73995	0.32059
4	Sb2	0.59032	0.04067	0.39459	37	N3	0.94382	0.35153	0.44966
5	Sb3	0.84661	0.25323	0.20757	38	N4	0.06257	0.63965	0.55142
6	Sb4	0.15374	0.74186	0.79333	39	N5	0.66174	0.25329	0.94641
7	Sb5	0.84989	0.75302	0.21292	40	N6	0.33748	0.74286	0.05438
8	Sb6	0.15153	0.24155	0.78871	41	O1	0.59853	0.08083	0.53511
9	Sb7	0.33256	0.26819	0.21150	42	O2	0.40716	0.91043	0.46735
10	Sb8	0.67074	0.72676	0.79045	43	O3	0.77748	0.78090	0.07735
11	H1	0.51600	0.33995	0.71386	44	O4	0.22486	0.20515	0.92492
12	H2	0.68718	0.41944	0.65246	45	O5	0.95408	0.96219	0.17577
13	H3	0.65764	0.22075	0.61080	46	O6	0.04128	0.03604	0.82109
14	H4	0.78051	0.18427	0.71169	47	O7	0.66601	0.69630	0.64426
15	H5	0.25075	0.65012	0.32170	48	O8	0.33673	0.29344	0.35771
16	H6	0.24328	0.88107	0.28435	49	O9	0.74420	0.54329	0.24544
17	H7	0.47890	0.66358	0.28506	50	O10	0.26066	0.44842	0.75854
18	H8	0.33953	0.77293	0.39003	51	O11	0.75651	0.19315	0.34998
19	H9	0.87031	0.27712	0.42146	52	O12	0.24868	0.79820	0.65105
20	H10	0.86159	0.50724	0.43389	53	O13	0.56509	0.34484	0.16146
21	H11	0.09494	0.30640	0.41103	54	O14	0.43486	0.65322	0.84009
22	H12	0.96208	0.31611	0.52372	55	O15	0.12327	0.16392	0.25576

23	H13	0.91175	0.68726	0.58999	56	O16	0.88258	0.82411	0.74289
24	H14	0.13676	0.71392	0.57924	57	O17	0.55297	0.97921	0.25301
25	H15	0.04583	0.67295	0.47719	58	O18	0.45002	0.01494	0.74798
26	H16	0.14287	0.48392	0.56810	59	O19	0.14716	0.56130	0.20054
27	H17	0.55949	0.19963	0.98550	60	O20	0.85548	0.43463	0.80643
28	H18	0.57798	0.38666	0.90539	61	O21	0.32901	0.21590	0.07266
29	H19	0.78077	0.15149	0.89878	62	O22	0.67040	0.78595	0.92837
30	H20	0.73619	0.28535	0.99572	63	O23	0.04331	0.69585	0.93585
31	H21	0.21622	0.84430	0.10148	64	O24	0.95730	0.30107	0.06560
32	H22	0.43880	0.79808	0.01575	65	O25	0.84029	0.77181	0.36485
33	H23	0.26534	0.70951	0.00439	66	O26	0.16328	0.22287	0.63629
